# Expanded phylogeny elucidates *Deinosuchus* relationships, crocodylian osmoregulation and body-size evolution

**DOI:** 10.1038/s42003-025-07653-4

**Published:** 2025-04-23

**Authors:** Jules D. Walter, Tobias Massonne, Ana Laura S. Paiva, Jeremy E. Martin, Massimo Delfino, Márton Rabi

**Affiliations:** 1 Dipartimento di Scienze della Terra, Università di Torino, Via Valperga Caluso 35, I-10125 Torino, Italy; 2 Department of Geosciences, Eberhard-Karls-Universität Tübingen, Hölderlinstraße 12, D-72076 Tübingen, Germany; 3 Laboratório de Paleontologia, Faculdade de Filosofia Ciências e Letras de Ribeirão Preto, Universidade de São Paulo, Ribeirão Preto, São Paulo, Brazil; 4 Université de Lyon, Université Claude Bernard Lyon 1, ENS de Lyon, CNRS, UMR 5276 Laboratoire de Géologie de Lyon: Terre, Planètes, Environnement, F-69622, 2 rue Dubois, Lyon, Villeurbanne France; 5 Institut Català de Paleontologia Miquel Crusafont, Universitat Autònoma de Barcelona, Edifici ICTA/ICP, c/ Columnes s/n, Campus de la UAB, E-08193 Cerdanyola del Vallès, Barcelona, Spain

**Keywords:** Palaeontology, Palaeoecology

## Abstract

Transmarine distribution and gigantism in the Late Cretaceous North American crocodyliform *Deinosuchus* has been difficult to reconcile with consistently inferred phylogenetic relationships to alligatorids, an otherwise freshwater and smaller-bodied group. We present an expanded phylogeny with increased spatiotemporally coherence that reinterprets species of *Deinosuchus* as stem-group crocodylians together with further putative alligatoroids, *Leidyosuchus canadensis* and the European *Diplocynodon* spp. (closely related to North American *Borealosuchus*). The novel topology elucidates the evolution of osmoregulation in Crocodylia and its close relatives by inferring plesiomorphic saltwater tolerance for *Deinosuchus* and the crown-group as well as secondary loss already in stem-group alligatorids. Divergence of Alligatoroidea coincided with extreme mid-Cretaceous sea level highs and the distribution of *Deinosuchus* across the Western Interior Seaway can be best explained by marine dispersal. Phylogenetic body-length analysis using a head-width proxy reveals phyletic dwarfism early in alligatoroid evolution and a reasonable total length estimate for the most complete specimen of *Deinosuchus riograndensis*. We find that gigantism in crocodyliforms is correlated with high-productive extensive aquatic ecosystems in the present and the past.

## Abstract (web summary)

An expanded phylogeny elucidates the evolutionary relationships of the “terror crocodile” *Deinosuchus*, shedding light on osmoregulation in crocodyliforms and body-size evolution patterns in the group.

## Introduction

The history of Alligatoroidea, the total (stem + crown) group of extant alligators and caimans (Alligatoridae), can be traced back to the Late Cretaceous of North America. Previous phylogenies of extinct taxa implicitly suggest that the early evolution of the group was already characterised by high morphological disparity and complex biogeographic histories, implying rapid rates of evolution^1–4^. Several early alligatoroids from the Late Cretaceous (e.g., *Brachychampsa* spp., *Albertochampsa langstoni*, *Stangerochampsa mccabei*) overall fit an expected ancestral body-plan for the group and were characterised by a relatively small size, short and blunt snout, overbite dental occlusion, enlarged 4th maxillary tooth, molariform posterior dentition, and an initial distribution restricted to Laramidia, the western part of North America once bisected by the extensive epicontinental Western Interior Seaway (WIS)^1,5^. A putative alligatoroid has been reported from the Atlantic coast but its age postdates the existence of the WIS^6^. The absence of unambiguous alligatoroids in Appalachia, together with depositional environments^7–9^, imply a shared lack of saltwater tolerance with extant alligatorids^10^. In contrast, other species recovered as early diverging members of the group, like *Deinosuchus* spp. and *Diplocynodon* spp. resembled crocodyloids or stem-group crocodylians in having partly interfingering dental occlusion, an occlusal notch between the premaxilla and maxilla for the 4th dentary tooth, enlarged 4th and 5th maxillary teeth, narrower and longer snout, larger or even gigantic body-size, and transmarine distribution^4,11^. In addition to its extremely large body-size, the ‘terror-crocodile’ *Deinosuchus*^12^ furthermore possessed highly derived morphological specializations^3^ and, together with *Diplocynodon*, have also utilised coastal marine habitats^13–15^. Moreover, the earliest alligatoroid record (~82 Ma) already includes both of these highly divergent morphotypes (*Deinosuchus* and *Brachychampsa sealyei*^16,17^) potentially implying a significantly earlier origin of the group. Most molecular divergence age estimates, however, do not suggest an earlier age than ∼90 Ma^18,19^. *Diplocynodon* further complicates the picture with its early branching position within the group that is in turn conflicting with an endemic European distribution and comparatively late first appearance date (late Paleocene)^1,4,20^. Morphology, body size, stratigraphic age, biogeography, and physiology are therefore markedly difficult to reconcile with alligatoroid phylogeny.

We present an expanded molecular-informed morphological phylogeny and find that character states previously diagnosing Alligatoroidea have a broader taxonomic distribution, thereby recovering both *Deinosuchus* and *Diplocynodon* as stem-crocodylians. The novel topology explains the geographic distribution of *Deinosuchus* with inferred salt tolerance and resolves the biogeographic history of *Diplocynodon*. In light of the resulting simpler biogeographic pattern, we hypothesise that the basal split of crown-group crocodylians, involving caiman and alligator ancestors, was triggered by extreme mid-Cretaceous sea level rise. Finally, body-size analysis combined with the new phylogeny elucidates the body size evolution of *Deinosuchus*, alligatoroids, and other crocodyliforms.

## Results and discussion

### Phylogenetic analysis

**Fig. 1:**
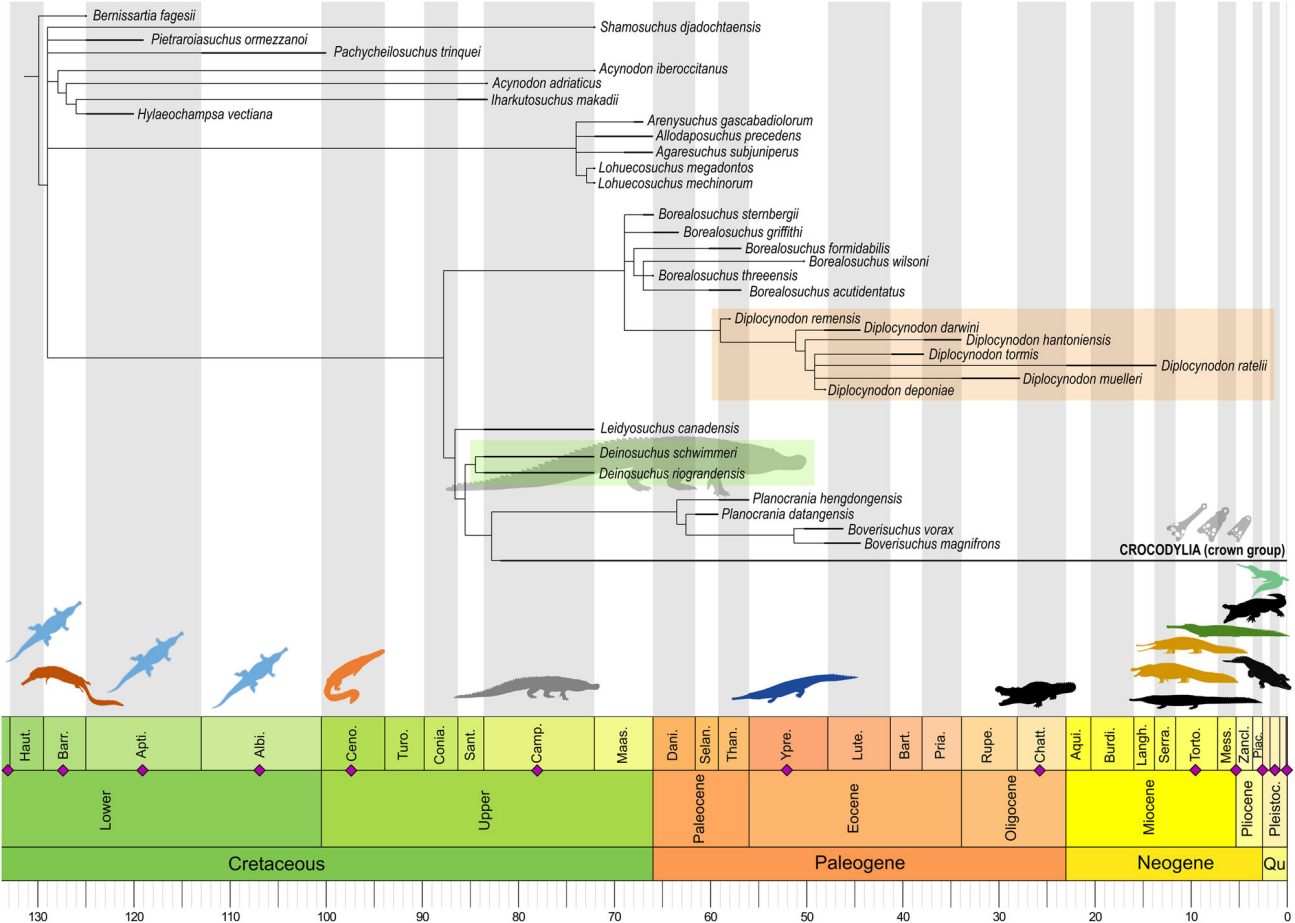
Reduced time calibrated strict consensus tree of the maximum parsimony analysis showing the position of *Deinosuchus* spp., *Leidyosuchus canadensis*, and *Diplocynodon* spp. as stem-crocodylians. *Borealosuchus griffithi* has two alternative positions, either as sister to *Diplocynodon* spp. or an early diverging placement within *Borealosuchus* spp. (Supplementary Data 1 “Walter et al_[TNT]”). Purple diamonds and silhouettes correspond to known occurrences of very large to giant (≥7 m) crocodyliforms. Each of the illustrated taxa is associated with high-productivity wetland or marine habitats (see Supplementary information Table S1 for a list of taxa and sources). Ages are in Ma.

**Fig. 2:**
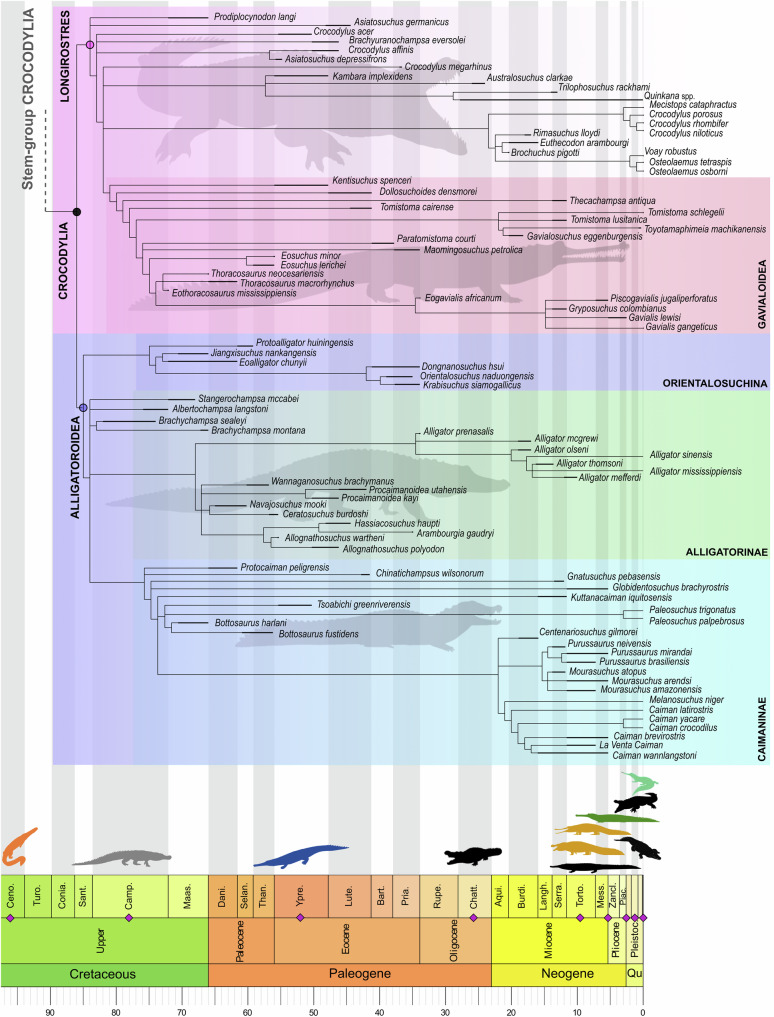
Reduced time calibrated strict consensus tree of maximum parsimony analysis showing the phylogeny of Crocodylia (i.e. crown-group) including Alligatoroidea. Purple diamonds and silhouettes correspond to known occurrences of very large to giant (≥7 m) crocodyliforms. Each of the illustrated taxa is associated with high-productivity wetland or marine habitats (see Supplementary information Table S1 for a list of taxa and sources). Ages are in Ma.

Expansions and modifications implemented in the present morphological taxon-character matrix included the merging of published data subsets, addition of characters and character states, rigorous redefinitions of select characters, homology reassessments, update of select character state scores, addition of taxa, and the inclusion of a molecular scaffold in the phylogenetic analysis (see Supplementary information and Supplementary Data 1). All the datasets combined here are expansions of Brochu [1999]^1^. Our maximum parsimony analysis recovered 506 most parsimonious trees (strict consensus tree reported in Figs. 1, 2 and Supplementary Fig. 1). The most unstable taxa (*Eocaiman* spp. and *Necrosuchus ionensis*) were pruned from the strict consensus tree to increase resolution (see Supplementary Fig. 1 for their respective placement). Alligatoroidea is formed by Orientalosuchina and its sister-group Alligatoridae (total group of caimans and alligators). Globidonta, the stem-based lineage comprising *Alligator mississippiensis* and all crocodylians more closely related to it than to *Diplocynodon ratelii*^1^, is here redundant with Alligatoroidea because *Diplocynodon* is recovered as a non-alligatoroid stem-crocodylian. In contrast to previous phylogenies (Brochu [1999]^1^ and all subsequent expansions), *Leidyosuchus canadensis*, *Deinosuchus* spp., and *Diplocynodon* spp. form the stem-lineage of crown-group crocodyliforms instead of Alligatoridae (Fig. 1). This is in part due to the addition of two key Paleocene taxa to the dataset, *Diplocynodon remensis* and *Borealosuchus griffithi*. *Diplocynodon* spp. is recovered as a monophyletic clade, nested in a polytomic *Borealosuchus* from North America. This polytomy is caused by the occasional recovery of *Borealosuchus griffithi* as the sister taxon to *Diplocynodon* spp. in some trees, sharing a hypapophyseal keel present up to the 12th vertebra and a greatly reduced quadratojugal spine.

The strict consensus tree recovers a basal polytomy within Alligatoridae formed by Alligatorinae, Caimaninae (including *Bottosaurus harlani*), and the North American Late Cretaceous taxa *Brachychampsa* spp., *Stangerochampsa mccabei*, and *Albertochampsa langstoni*. The latter have two alternative positions either in Alligatorinae or along stem-Alligatoridae.

### Phylogenetic body-size estimation

**Fig. 3:**
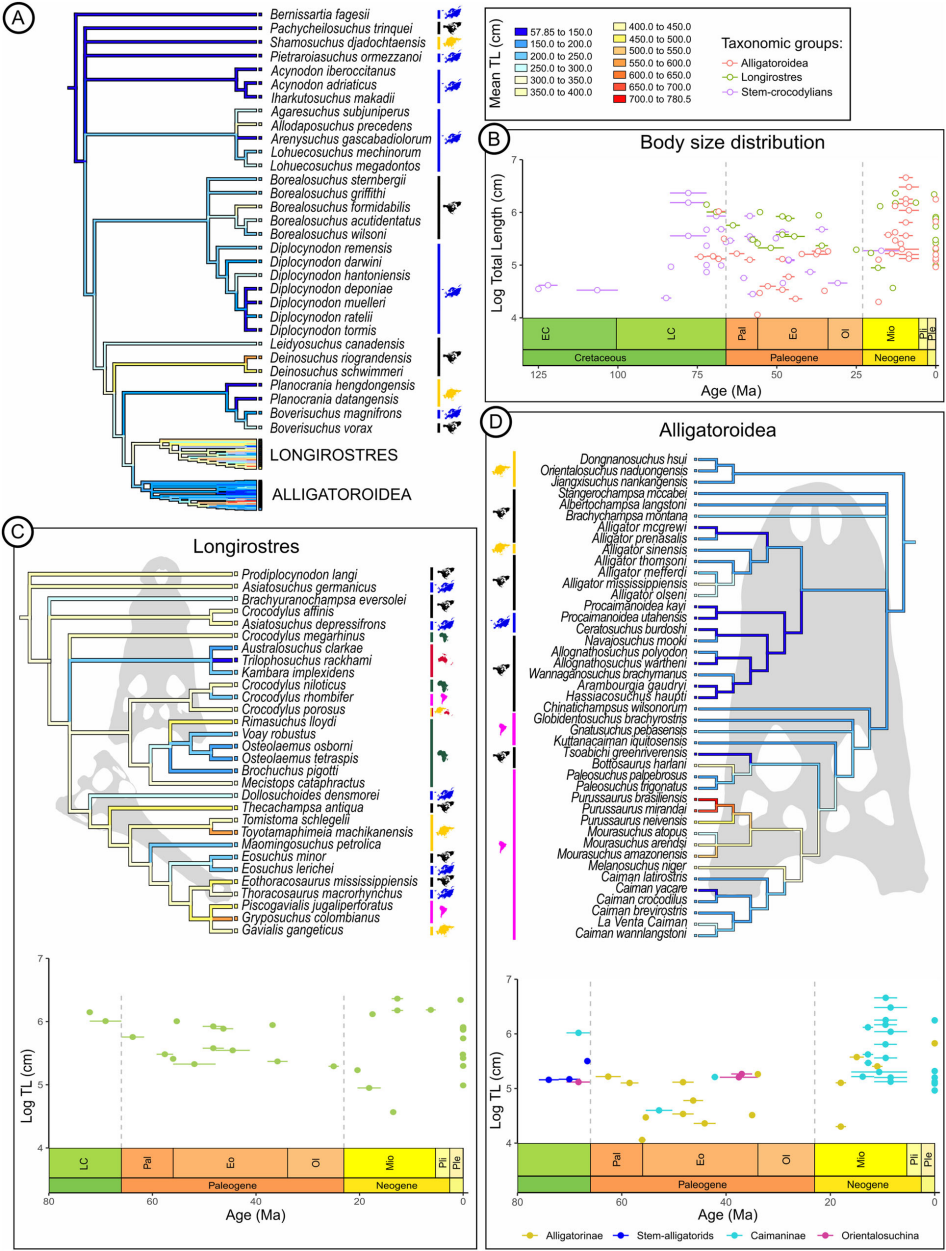
Parsimony ancestral state reconstruction of the phylogenetic mean total length estimations (Supplementary Data 2.3, Table S1) plotted on the strict consensus tree using equal branch length. (**A**) stem- and crown-group Crocodylia; (**B**) body size distribution of all taxa against geological time; (**C**) Longirostres and body size distribution of the group through time; (**D**) Alligatoroidea and body size distribution of the group through time. Ages are in Ma.

In light of the novel topology, with *Deinosuchus* spp., *Leidyosuchus canadensis*, and *Diplocynodon* spp. removed from Alligatoroidea, all early representatives of this clade were relatively small-sized and we therefore wanted to test whether the origin of alligatoroids was characterised by phyletic dwarfing. In addition, we wanted to test the impact of the phylogenetic correction and the current topology on body-size estimates of *Deinosuchus* spp. relative to previous studies. Previous works addressing *Deinosuchus* body-length did not employ phylogenetic correction and instead included it in a regression of distantly related extant taxa (*Crocodylus porosus*, *Alligator mississippiensis*^12^) with likely different body proportions^5^. Phylogenetic body-size estimates provide results that take into account the known or reconstructed proportions of close extant relatives^21^. We here used the same individual of *D. riograndensis* as previous non-phylogenetic work (TMM 43620-1^11,12^ see Supplementary Data 2.3, and Supplementary information for the list of sources) but a skull width proxy^21^ instead of skull/mandible length, making the comparison only partly appropriate. However, skull width has been argued to be more reliable as it is less influenced by differences in body proportions caused by a long snout, a trait present in species of *Deinosuchus*^3,21^. Mean values of estimated body-length together with lowest and highest quantiles are provided in Supplementary Data 2.3, and a parsimony ancestral state reconstruction of size bins is shown in Fig. 3. We here divide body-sizes according to the following categories based on extant species: total length (TL) estimations of ca. 1.5 m and lower are considered small size, representing the general body-size of some fossil species and exceptionally small individuals of extant species. Medium size category includes TL estimations between 1.5 and 4.0 m, and comprises all extant species. Large size category includes TL estimations between 4.0 and 7 m and includes large to maximal body length of extant species^22^ (e.g. *Crocodylus porosus*, *Gavialis gangeticus*). TL estimations above 7 m are considered gigantic and are only known in extinct species^23,24^. The divergence of Alligatoroidea was coupled with size reduction and an ancestral body-length of 150–200 cm compared to 250–300 cm of the outgroup. Most Paleogene alligatoroids of North America retained a medium to small size or went through further shrinking including some taxa that are inferred to be relatively more terrestrial^25^. Larger size independently evolved in the lineage containing extant *Alligator mississippiensis* and its extinct Miocene relatives, as well as extant *Melanosuchus niger* and a clade of South American Miocene caimanines, involving independent gigantism in *Purussaurus* spp. and *Mourasuchus amazonensis* according to the present topology (Fig. 3). Species of the stem-crocodylian *Deinosuchus* acquired giant sizes although our estimates give shorter, and possibly more realistic, total body length compared to previous work^12^. The detailed results of the analysis are available in Supplementary Data 2.3.

### Stem-crocodylian affinities of *Deinosuchus* can explain transmarine distribution through saltwater tolerance

Our maximum parsimony analysis resulted in a topology where several taxa previously considered to represent stem-alligatorids (i.e., all studies descending from that of Brochu^1^: e.g. refs. ^3,4,20–23,26,27^), such as *Deinosuchus* spp., *Leidyosuchus canadensis*, and *Diplocynodon* spp., are reinterpreted as stem-group crocodylians, regardless of the addition of the molecular scaffold (Fig. 1). The placement of these taxa along stem-crocodylians is more consistent with their plesiomorphic morphology relative to other alligatoroids^1^, their stratigraphic and geographic distribution, and the fact that *Diplocynodon* shares a number of remarkable derived traits with the stem-crocodylian *Borealosuchus*. Some of our results are congruent with recent published analyses using different datasets: some but not all analyses of Groh et al.^28^ employing quantitative characters recovered *Diplocynodon* spp. and *Leidyosuchus canadensis* as stem-crocodylians^28^ and Rio and Mannion^4^ recovered a paraphyletic *Diplocynodon* sister to the lineage of Longirostres also using quantitative characters in some of their analyses. Muscioni et al.^29^, based on a more similar dataset to that of the present study, found *Diplocynodon*, *Leidyosuchus canadensis*, as well as *Deinosuchus riograndensis* in a polytomy with Crocodylia.

Species of *Deinosuchus* from the Late Cretaceous (Campanian) coastlines of the North American Western Interior Seaway (WIS) and Atlantic have been considered among the largest crocodyliforms of all time with a body length previously estimated around 10 m^3,12,30,31^. Bite mark evidence suggests their diet even included large dinosaurs^11,32,33^. The first phylogeny including *Deinosuchus*^1^ found this taxon as an early diverging member of total-group Alligatoridae. All subsequent works, including a recent comprehensive revision of *Deinosuchus*^3^, confirmed this placement despite marked morphological contrast relative to contemporaneous early alligatoroids, such as *Brachychampsa*^16,17^.

The phylogeny herein, on the other hand, finds *Deinosuchus schwimmeri* and *D. riograndensis* outside Alligatoroidea, along the stem-lineage of crown-group crocodyliforms (Crocodylia). In other words, *Deinosuchus* was neither a ‘greater alligator’^34^ or a ‘terror crocodile’^12^. Our expanded dataset is overlapping in taxon and character sample with previous studies including *D. schwimmeri* and *D. riograndensis*^1,23,26^ and our character state scorings follow the latest work updating this taxon^3^. The more stemward position in our study is largely due to the addition of two key Paleocene taxa to the dataset, *Diplocynodon remensis* and *Borealosuchus griffithi*, which resulted in the placement of *Diplocynodon* spp., *Deinosuchus* spp. and *Leidyosuchus canadensis* as stem-crocodylians in our analysis. These three taxa share the above listed differences from true early alligatoroids (except large body size) and their stem-crocodylian placement is retained even with the removal of the molecular scaffold from our analysis. *Deinosuchus* is excluded from Crocodylia by lacking the following traits among others: an incisive foramen that abuts the toothrow, a single largest maxillary alveolus that is the 5th, and a frontoparietal suture that makes a modest entry into the supratemporal fenestrae. Some previous alligatoroid synapomorphies are now optimised as crocodylian plesiomorphies (Supplementary information, 2.1). This novel stem-crocodylian position of *Deinosuchus* spp. is consistent with its early stratigraphic age (early Campanian), plesiomorphic morphology^3^, and would also imply less homoplasies^3^ (e.g. character 71:0 was convergent with *Borealosuchus* but here optimised as a plesiomorphy for Crocodylia). Species of *Deinosuchus* nevertheless still share convergent characters with long-snouted taxa^3^ even in the current topology. Scoring *Deinosuchus riograndensis* in a different dataset (Rio & Mannion^4^) resulted in a relatively deeply nested position within Alligatoroidea, but we nevertheless find this highly doubtful due to the particularly poor stratigraphic fit of this topology and the ambiguous synapomorphies uniting Alligatoroidea, some of which are present only in a few of the ingroup taxa whereas others are present in several of the outgroup taxa as well (Supplementary information, 2.2).

**Fig. 4:**
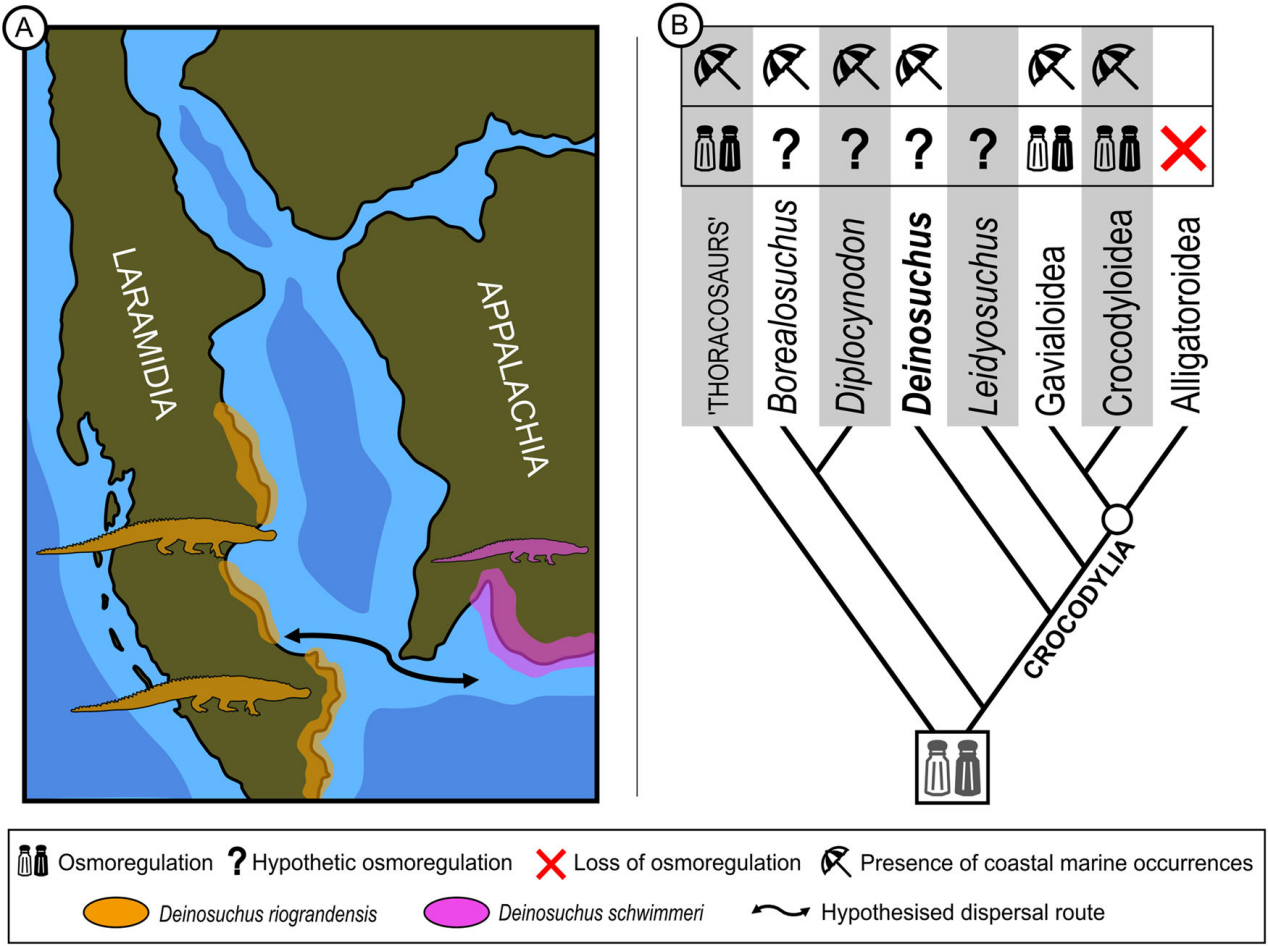
Palaeobiogeography of *Deinosuchus* spp. (**A**) Distribution of *Deinosuchus riograndensis* and *D. schwimmeri*^3^ during the Campanian around the Western Interior Seaway (WIS). (**B**) parsimony ancestral state reconstruction (equal branch length) of osmoregulation in Crocodylia and close relatives using presence/absence of salt glands, stable isotopes, and coastal marine occurrences as proxies^11,13–15,17,35–40,42,44,120^. The topology is from the present study except for ‘thoracosaurs’ for which we follow a more appropriate tip-dated work^41^. The analysis suggests potential plesiomorphic saltwater-tolerance for *Deinosuchus* and Crocodylia with early loss in Alligatoroidea. The distribution of *Deinosuchus* may be explained by dispersal across the WIS. Map is redrawn from^118^, early to late Campanian. Distribution of *Deinosuchus* spp. follows^3,11,17^ and references therein.

*Deinosuchus* as a stem-crocodylian is furthermore more consistent with its biogeographic distribution on both sides of the Western Interior Seaway (WIS) in contrast to early members of true early alligatoroids restricted to the West until the retreat of the seaway^6^. Cossette & Brochu^3^ recently proposed that species of *Deinosuchus* were allopatric, with *D. riograndensis* distributed along the western coast of the WIS (Laramidia) and *D. schwimmeri* along the eastern and Atlantic coasts (Appalachia). The authors suggested that speciation in the clade occurred through vicariance, during the opening phase of the WIS, separating *Deinosuchus* ancestral populations. The main rationale behind this was due to the supposed alligatoroid affinity of *Deinosuchus* with extant relatives lacking lingual salt glands, which would render them incapable of osmoregulation and prolonged saltwater exposure required for crossing the extensive WIS^11,35–39^. The herein proposed stem-crocodylian position, however, no longer infers lack of osmoregulation and may explain the distribution of *Deinosuchus* through dispersal across the WIS. Saltwater tolerance is inferred to be plesiomorphic for Longirostres^4,40^ and may well have been plesiomorphic for Crocodylia as many stem-group taxa close to the crown appear to be euryhaline^13^. These include marine thoracosaurs (recovered as stem-crocodylians in tip-dated phylogenies)^41^, potentially *Diplocynodon*, occasionally recovered from marginal marine settings^14,15^, and *Deinosuchus* itself, which is mostly recorded from estuarine or nearshore habitats such as coastal plains, deltas or platform contexts^11^. Moreover, stable isotope analysis of carbon and oxygen from eastern *Deinosuchus* tooth enamel samples suggest consumption of seawater or marine prey^13^, the latter also supported by bite mark evidence of predation on nearshore marine turtles^11^. The simultaneous disappearance of *Deinosuchus* from the fossil record (supposed extinction) with the draining of megawetlands along the WIS and Atlantic coasts (including complete retreat of the former) later during the Cretaceous is furthermore consistent with a lifestyle linked to coastal habitats^42,43^. *Borealosuchus* may serve as an additional example for salt-tolerant stem-crocodylians as it is known to co-occur with *Deinosuchus* in the Moorville Chalk of Alabama, a marginal marine setting^44^. Taking this data together, our parsimony ancestral state reconstruction, including data from this study, implies that the presence of saltwater tolerance (osmoregulation) may have been plesiomorphic for Crocodylia (Fig. 4) and simply retained in species of *Deinosuchus*. Nevertheless, this does not mean that osmoregulation was necessarily achieved through the presence of lingual salt glands. Saltwater tolerance, possibly including lingual salt glands, were subsequently lost in alligatoroids and *Gavialis*^45^. Previous phylogenies left it ambiguous whether salt glands (with no known osteological correlates) were already lost in stem-group alligatoroids only^1^ and recent work proposed that salt tolerance may have been only lost in the crown-group ^46^. The topology of the present study, however, implies the loss of effective osmoregulation (possibly including lingual salt glands) in the stem-lineage as all early true alligatoroids in the new phylogeny come from freshwater deposits^7–9^ (Fig. 4).

Morphological differences in western *Deinosuchus riograndensis* and eastern *D. schwimmeri* are relatively minor except for body size, with known specimens of the western taxon being considerably larger^3^. If speciation took place, dispersal is more consistent with the novel phylogeny than vicariance. Isolation would have been maintained through the episodic nature of the dispersal due to the significant width of the seaway. A literal reading of the fossil record would imply an east to west dispersal as eastern records are so far somewhat older^11^, but this simply may be a sampling bias in the fossil record of *Deinosuchus*.

### Body-size estimation of *Deinosuchus* and evolution of gigantism in crocodyliforms

Previous work estimated the total body-length of *Deinosuchus* spp. between ca. 8 and 12 m (up to 12 m^11^; 7.67 and 10.640 m^31^; and 7.73 to 8.13 m^47^). Total body-length has been shown to more strongly correlate with head-width than with cranial length given the variability of rostral proportions among crocodylians^48^. Because *Deinosuchus* has a relatively long snout^3^, previous approaches^11,12^ may have overestimated the total length of this taxon, as they based their regression on shorter-snouted taxa, *Alligator mississippiensis* and *Crocodylus* spp. Our method of estimation differs from that of previous studies in employing a skull width proxy^21^ instead of femur^31^, mandible^11,12^ or vertebra^11,47^ and furthermore includes a phylogenetic correction to bypass the use of a unique extant proxy (e.g., *Alligator mississippiensis*, *Crocodylus porosus*) as body size proportions show strong phylogenetic structuring in crocodylians^21^. The phylogenetic approach, however, is still only sampling living crocodylians, a fraction of past morphological diversity, and body proportions of extinct taxa, particularly those (but not only) outside the clade may have significantly differed (including *Deinosuchus*). This implies that, following O’Brien et al.^21^, outer quartile estimates should be considered for taxa showing “sufficient biological evidence to presume that body size should be meaningfully greater or lesser than the mean estimate (e.g., terrestrial versus fully aquatic denizens, tail, or head size atypically large or small in a given taxon)”. Concerning the present estimate, we consider the 97.5 percentile estimate (7.66 m total length for *Deinosuchus riograndensis* and 6.37 m for *Deinosuchus schwimmeri*; see Supplementary Data 2.3) more realistic than the mean (5.80 m for *Deinosuchus riograndensis* and 4.83 m for *Deinosuchus schwimmeri*). Our reasoning is that the 97.5 percentile estimates lie between the conservative mean of our estimates and previous non-phylogenetic estimations using cranial length. Our estimate of *D. riograndensis* is based on the skull of the same individual as in Schwimmer^11^ (9.8 m) and Erickson and Brochu^12^ (8.43 to 9.10 m) who, however, both used the length of the lower jaw of the specimen. Furthermore, previous applications of phylogenetic body-size estimations systematically found lower mean estimates compared to non-phylogenetic methods^21,48,49^. The maximal size of *D. riograndensis* was, nevertheless, likely larger than even our 97.5 percentile estimates as evidenced by the larger size of the holotype specimen (AMNH 3073) compared to the specimen used in our study (TMM 43620-1).

Very large to gigantic body size (here defined as ≥ 7 m total length) has repeatedly evolved during the history of crocodyliforms and represents a wide range of taxa across the phylogenetic tree known from the Cretaceous to the present^50–54^ (Figs. 1 and 2). Previous work has underlined the importance of aquatic to semi-aquatic lifestyle^52^ and temperature^50,55,56^ in driving large body-size in crocodyliforms, but the triggers of extreme sizes across clades have not been explicitly addressed. We propose that *Deinosuchus* exemplifies an ecological pattern that has been universally characteristic of giant crocodyliforms and their ecosystems. Species of *Deinosuchus* were inhabitants of a marginal mega-wetland along the WIS and the Atlantic east coast, sustaining other extremely large megafauna species during highly favourable mean annual temperatures for growth^11,42^. Other species of giant crocodyliforms (e.g. *Rhamphosuchus*, *Phosphatosaurus*, *Sarcosuchus*, *Crocodylus porosus*; for a complete list, see Supplementary information Table S1) are/were likewise associated with extraordinarily productive and spatially extensive warm marine or wetland ecosystems including other megafauna. A proposed relationship of extreme body-size and ecosystem productivity is well in accordance with a global analysis of phanerozoic animals, excluding crocodyliforms, which found that the critical factor for gigantism is an unusually highly structured ecosystem in which productivity imposes only exceptionally low limits to sustain extraordinary body-size^57^. Favourably constant warm temperatures^42^, an evolutionary shift to rapid growth rates early in ontogeny ^51^, and elevated long-term ecosystem productivity can be therefore considered key for the evolution of gigantism in crocodyliforms. The existence of very large, ~ 7 m long crocodylians in the present and Pleistocene icehouse conditions (*Crocodylus porosus*^51^, *C. thorbjarnarsoni*^23^, *Crocodylus* sp.^58^) suggests that, contrary to what the literal reading of the fossil record implies, a world with enormous crocodyliforms may have been rather the norm than the exception in the last ~ 130 million years.

### Systematics of *Diplocynodon* and implications for Euramerican paleobiogeography

Another novel aspect of the phylogeny presented in our study is the placement of the European Cenozoic *Diplocynodon* outside Crocodylia in a monophyletic group with species of North American *Borealosuchus* (Fig. 1; *B. griffithi* has two alternative positions within the clade). This novel result is largely the impact of the addition of the geologically earliest known species of *Diplocynodon*, *D. remensis* (late Paleocene) as well as the early Paleocene *Borealosuchs griffithi* to our dataset. Pre-cladistic work has long acknowledged the high morphological similarities between *Diplocynodon* and *Borealosuchus*^59–62^ but this signal was never recovered in phylogenetic analyses (e.g. refs. ^1,4,20,23,26,63–66^). Several plesiomorphies of *Diplocynodon* are shared with *Borealosuchus* and *Deinosuchus* but are absent in typical alligatoroids (e.g., long snout, confluent 3rd and 4th dentary alveoli, 4th and 5th maxillary alveoli equal in size, notch between premaxilla and maxilla in adults). A key character previously placing *Diplocynodon* in Alligatoroidea is the presence of a premaxillary-maxillary pit (instead of a notch) for the reception of the dentary fang early in ontogeny. The notch seen in adult *Diplocynodon* (the inferred plesiomorphic condition for Crocodylia) is secondary, developed later in ontogeny due to abrading occlusion^1^. However, the early ontogenetic pit is not confirmed for all species of *Diplocynodon* and more importantly, the condition remains unknown for *Borealosuchus* spp. and other stem-crocodylians^1,64^. The taxonomic distribution of the early ontogenetic premaxillary-maxillary pit is therefore ambiguous and might diagnose a more inclusive clade. On the other hand, some of the shared traits between *Diplocynodon* and *Borealosuchus* are derived and include the presence of ventral armour made of bipartite osteoderms (otherwise only known in *Tsoabichi greenriverensis* and extant caimanines), the exclusion of the nasals from the external naris, unequal anterior processes of the surangular, and the presence of occlusion pits between the 7^th^ and 8^th^ maxillary alveoli. Indeed, our phylogeny optimises these three character states as synapomorphies uniting the clade *Diplocynodon* + *Borealosuchus*.

This topology has far better stratigraphic fit for species of *Diplocynodon* and *Borealosuchus* compared to previous phylogenies: for the first time, we recover the oldest species (i.e. the late Paleocene *D*. *remensis* and the Late Cretaceous *B*. *sternbergii*) of each clade as also the earliest branching taxa. Although the early Paleocene *Borealosuchus griffithi* has an unresolved position in our phylogeny, several of our most parsimonious trees place this species as the sister taxon of *Diplocynodon* spp. Under this particular topology, *Borealosuchus* is paraphyletic and the ghost lineage of nearly 20 Myrs inferred by previous phylogenies (with *Diplocynodon* spp. as early branching alligatoroids) are reduced to ca. 6 Myrs. Notably, *Diplocynodon remensis* and *Borealosuchus griffithi* both share the derived trait of a shallow recess on the medial wall of the premaxillary-maxillary notch^20^, a character yet to be included in a phylogeny and explored for other species of *Diplocynodon* and *Borealosuchus*. A clade of (*Borealosuchus* spp. (*B. griffithi* + *Diplocynodon* spp.)) is implying a single dispersal from North America to Europe during the Paleocene. The earliest known occurrence of *Diplocynodon* in the late Paleocene of Europe (*Diplocynodon remensis*^20^) may underestimate the timing of the dispersal since a high number of North American species immigrated via Greenland and Scandinavia to Europe already during the early and middle Paleocene using the De Geer route^67–75^. In light of the herein recovered stem-crocodylian status of *Diplocynodon* (Fig. 4), a dispersal through a marine route cannot be excluded. A comprehensive revision of Paleogene *Borealosuchus* may contribute to testing or further refining these hypotheses.

### Implications for crocodyliform extinction across the Eocene/Oligocene cooling

Our topology has implications for phylogenetic patterning of high crocodyliform extinction rates across the cooling climate of the Eocene/Oligocene transition in North America and Europe^42^. Previous phylogenies implied that all crocodyliform survivors in terrestrial ecosystems were alligatoroids, including *Diplocynodon*^1,76–80^. In contrast, the topology herein suggests a survival pattern less structured by phylogeny: in Europe, the stem-crocodylian *Diplocynodon* spp., whereas in North America, the alligatorine lineage leading to *Alligator* spp. crossed the transition^63,81^. On the other hand, the herein proposed sister-taxon of *Diplocynodon*, the North American *Borealosuchus*, did not survive into the Oligocene (with the last occurrence known from the middle Eocene; *Borealosuchus wilsoni*^4,64^). This divergent survival pattern may be best explained by independent cold adaptation in *Diplocynodon* and the lineage leading to *Alligator*. It has been previously proposed that following global cooling, shrinking habitats led to increased competition between large and small-bodied crocodylians and selective extinction of small-sized taxa^52^. An alternative explanation consistent with our body-size analysis, at least for alligatoroids, is that small-sized lineages evolved large body-sizes during the Neogene without selective extinction of small taxa.

### Early alligatoroid evolution

In contrast to previous global phylogenies (refs. ^1,3,4,21,23,26,27,63,82,83^), the analysis herein advocates a less inclusive alligatoroid clade (Fig. 2). Under this topology, previously recovered synapomorphies for Alligatoroidea, including *Deinosuchus* (e.g. foramen aëreum set in from the margin of the retroarticular process, occlusion of anterior dentary teeth lingual to maxillary teeth, quadratojugal spine located between the posterior and superior angles of the infratemporal fenestra; see Cossette and Brochu^3^) are reoptimized to diagnose a more inclusive clade (Supplementary information 2.1). The earliest representatives of Alligatoroidea are herein restricted to only a few taxa from the Late Cretaceous of North America (*Brachychampsa* spp., *Stangerochampsa mccabei*, and *Albertochampsa langstoni*^8,9,16^), here recovered either as representatives of stem Alligatoridae or the early branching Alligatorinae (total group of *Alligator* spp.). Both alternatives would make the name Globidonta^1^ redundant with Alligatoroidea. This restricted taxonomic composition has a better stratigraphic fit owing to the removal of the stratigraphically old and morphologically specialised *Deinosuchus*. It also implies less homoplasy and eliminates the phenetic contrast with taxa previously inferred as early branching alligatoroids. In turn, taxa replaced as stem-crocodylians are arranged in a topology with a better stratigraphic fit, such as *Diplocynodon* and *Borealosuchus* (see above).

Almost all Cretaceous alligatoroids, under the novel topology, share a relatively reduced body-size compared to other non-alligatoroid crocodylians, suggesting phyletic dwarfism^84^ early during the evolution of the group (Fig. 3). An exception is *Brachychampsa montana*, which retains a body-size comparable to the ancestral condition of Crocodylia. *Bottosaurus harlani* is another large-sized early putative alligatoroid^6^ but its affinity with the group has been questioned^63^. Additionally, all Cretaceous alligatoroids share a short and blunt snout, full overbite dental occlusion, a caniniform 4th maxillary tooth, crushing posterior dentition, a North American Laramidian distribution, and freshwater habitat. The only exceptions in our topology are representatives of Late Cretaceous–Paleogene Orientalosuchina that are here recovered as the earliest diverging alligatoroids and are characterised by plesiomorphies including a 5th maxillary caniniform tooth, a notch between the premaxilla-maxilla for the reception of the 4th dentary tooth, as well as a strictly Asian distribution^26,83,85–87^. The global phylogenetic relationships of Orientalosuchina, however, has been unstable and studies variously placed them in stem-group Alligatoridae^26,63,83,88^, Crocodyloidea^86–89^, Caimaninae (Walter et al.^63^ under equal weighting), and Australian Mekosuchinae^90^. The alligatoroid position of Orientalosuchina in our phylogeny is not well supported since most synapomorphies uniting the two groups are unknown in most orientalosuchines and the outgroup (Supplementary information 2.1 and Supplementary Data 1). Additionally, their endemic Asian distribution is in contrast with that of all other early alligatoroids and would imply an early dispersal to Asia during the Late Cretaceous, a route otherwise poorly supported^26^.

Except for Orientalosuchina, the simplified paleobiogeographic pattern inferred by our topology is consistent with a vicariant divergence between Alligatoroidea and its sister-clade, Longirostres (Crocodylidae + Gavialidae^18^). Most early and living representatives of Longirostres have an Asian origin and/or distribution^4^, whereas all definite early alligatoroids are North American. The age of this divergence has been estimated into the early Late Cretaceous (ca. 90–100 Mya)^18,19,41,91^ coinciding with a period of extreme sea level increase culminating in the highest sea level during the entire Mesozoic and Cenozoic eras (90–94 Mya, Turonian)^92^. Exceptionally high sea level may have isolated North American and Asian ancestral stem-crocodylians by posing a wide marine barrier, even for saltwater tolerant species, across Beringia. In contrast, warm climate would have instead favoured high latitude faunal connections during the Turonian (Cretaceous thermal maximum^93^) and is therefore unlikely to have driven divergence. Based on our topology, we infer that alligatoroids, as a freshwater clade in the interior of the continent, secondarily lost osmoregulation ability (and possibly lingual salt glands) early during their evolution (Fig. 4). Our parsimony body-size analysis recovers a minimum of 20% reduction in total body length (TL) at the root of Alligatoroidea, involving a shrinkage from 200–250 cm to 150–200 cm. This reduction reaches up to 40% (from 200–250 cm to <150 cm) when early alligatorines such as *Ceratosuchus burdoshi* are considered (Fig. 3). Low body-size disparity and shrinking early in the evolution of the group is a novel finding of this study as previous body-size analyses employed different topologies (i.e. not accommodating molecular topologies in the phylogenetic framework, placing *Diplocynodon* and *Leidyosuchus* as early alligatoroids, and excluding *Deinosuchus* from the sample^52,54,94^). Small body size was broadly retained during the Paleogene and gigantic forms only evolved in the Neogene among caimanines (*Purussaurus* and *Mourasuchus* from South America). In addition, large size (3–4 m) independently evolved in the lineage of extant *Alligator mississippiensis*. Godoy et al.^52^ proposed that Cenozoic Crocodylia body-size progressively increased in response to selective extinction of smaller-bodied taxa due to global cooling-induced habitat loss and associated increased competition. However, as we demonstrate here, in alligatoroids at least, there were no large-bodied taxa before the Neogene and instead, small-bodied taxa may have simply evolved into large-bodied ones. In line with this, Brochu & Camp^95^ suggested that small-sized Paleogene specialists with crushing dentition evolved into larger-sized generalists in the Neogene, although we note that a specialised morphology may not be necessarily associated with narrow niche^96^. Under our topology, we detect a minor body-size increase in *Alligator* following the Eocene/Oligocene extinction of all other North American crocodylians^79,97^.

## Methods

### Phylogenetic analysis

We expanded and combined previous morphological taxon-character datasets^3,26,27,63,83,98–100^, that are themselves expand on previous work^1,23,76,82^. Our character/taxon dataset consists of 219 discrete morphological characters and 128 taxa, including taxa absent from other recent global datasets (e.g. *Deinosuchus* spp., Orientalosuchina, *Diplocynodon remensis*, and *Borealosuchus griffithi*). Character definitions and scorings were managed in Mesquite version 3.7^101^. Multistate characters forming a morphocline were treated as ordered. Ordering, however, does not impact the position of *Diplocynodon*, *Leidyosuchus* or *Deinosuchus*, with the exception that *Deinosuchus* is retrieved as the earliest diverging alligatoroid in few of the trees, a position inconsistent with circumstantcial evidence (see Discussion). In total, 19 new taxa were added, 20 additional characters, and over 50 character scores were updated relative to the parent dataset^26^. For details of the dataset and analysis see Supplementary information. The dataset is available in Supplementary Data 1.

The maximum parsimony analysis was performed in TNT 1.6^102^ using a manually implemented molecular scaffold^91^ based on the topology recovered by Oaks^18^ (see Supplementary information for topology; the constraints are embedded in the tnt file): the scaffold constrains extant species relationships on the basis of molecular topology and allows fossil taxa to be placed within this topology based on morphological characters. Enforcing constraints enables the recovery of Longirostres, the consensual clade uniting *Gavialis gangeticus*, *Tomistoma schlegelli* and *Crocodylus niloticus* in accordance with molecular^18,41,103^ and some recent morphological topologies^4^. The parent datasets here combined and expanded, however, are unable to recover this clade and therefore some previous studies employed a molecular scaffold^26,63,91^. It has been recently demonstrated that molecular scaffolds represent an appropriate alternative of total-evidence approaches for fossil crocodylian phylogenetic inference^91^. Nevertheless, the scaffold has apparently no impact on the stem-crocodylian placement of *Deinosuchus, Diplocynodon* or *Leidyosuchus* in our analyses, unless the key taxa, *D. remensis* and *B. griffithi*, are removed from the dataset.

A first round of New Technology Search was performed as advised for large datasets^104^, enabling all search algorithms (Sectorial search, XSS enabled; Ratchet; Drift; Tree fusing) and stabilising the consensus 5 times. A second round of New Technology Search was then conducted, but using the trees saved from RAM, disabling Sectorial searches. The consensus tree was obtained from trees recovered by the second round of calculation. Figures 1 and 2 were created using the R package *strap* developed by Bell and Lloyd^105^, using 1 Ma as minimum branch length and using taxon ages from Darlim et al. ^91^ and sources reported in Supplementary information (see Supplementary Data 1 for the complete list of ages).

### Phylogenetic body-size analysis

The estimation of body sizes of extinct species was undertaken using a Bayesian phylogenetic approach and the application of regressions based on head width (HW) and total body length (TL) measurements from extant crocodylians^21,48^. We expanded previous regression datasets^21,48^ by adding the extant *Osteolaemus osborni* and thus including a total of 25 species and 207 specimens. Head width, measured as the distance between the extremes of the quadrates, was collected using ImageJ^106^ for 91 fossil and 16 extant taxa in our phylogenetic analysis (Supplementary Data 2.3, Table S1). For topological structure, we used the consensus tree obtained in the present study (Figs. 1, 2; Fig. S1), including time calibration. This involved adding age information for all tips sourced from Darlim et al.^91^ and other references (Supplementary information). The calibration employed 5 million years as minimum branch lengths (*mbl* method^107^) in the *timePaleoPhy()* function of the *paleotree* package^108^ in R 4.3.1^109^.

Total body length was estimated through the BayesModelS method^110^ for phylogenetic predictions, which adopts a Brownian motion model and employs a Monte-Carlo Markov-Chain (MCMC^21,48,110,111^). The phylogenetic signal values utilised by BayesModelS method were extracted from the *phytools* package^112^ through *phylosig()* function. The entire protocol, data sources, along with additional details are available in Supplementary Data 2, including packages such as *car*^113^, *MASS*^114^, *caper*^115^, *evomap*^116^, and *rms*^117^. A parsimony reconstruction of ancestral states was used to plot the discretised continuous values of mean total length in Mesquite^98^ on the strict consensus tree (see Supplementary Data 2.3). Temporal and taxic distribution of body size were visualised using *ggplot2*, *deeptime* and *jpeg* R packages.

### Figures

All figures were produced using the free image editor GIMP and free vector graphics editor Inkscape (https://www.inkscape.org). The silhouette used for *Deinosuchus* in Figs. 1, 2 and 3 was created based on the artwork of Andrey Atuchin under the Creative Common BY-SA 4.0 license (https://creativecommons.org/licenses/by-sa/4.0/). Remaining silhouettes used to illustrate clades and taxa in Figs. 1 to 4 were sourced from PhyloPic (https://www.phylopic.org/) and are in the Public Domain except for *Gryposuchus* (https://www.phylopic.org/images/d4225b65-a520-42ae-b3ab-8725778a8403/gryposuchus-pachakamue); *Paleosuchus* (https://www.phylopic.org/images/9289a813-73ad-4644-b738-d9be619d8219/paleosuchus), and *Purussaurus* (https://www.phylopic.org/images/b7fedb04-759e-4f1a-b8bb-d0faefc64e75/purussaurus-neivensis) by Armin Reindl and are accessible for reuse under the Creative Commons BY-NC 3.0 license (https://creativecommons.org/licenses/by-nc/3.0/deed.en); and *Euthecodon*, by Smokeybjb (https://www.phylopic.org/images/a1e916c4-e020-4657-932b-d74ec6c08e0a/euthecodon-brumpti); *Crocodylus anthropophagus* by Nobu Tamura (vectorised by Julian Bayona, https://www.phylopic.org/images/c60b0e39-1437-4bb4-8940-f6da3d943adf/crocodylinae-anthropophagus); *Stomatosuchus* by Stanton F. Fink (vectorised by Julian Bayona, https://www.phylopic.org/images/f7d45c6d-e506-4826-8ffe-3f75d588d378/stomatosuchus-inermis); *Phosphatosaurus* by Nobu Tamura (vectorised by Julian Bayona, https://www.phylopic.org/images/13ff6eb0-a671-44d8-8a51-8b9f95d49403/dyrosaurus-phosphaticus) accessible for reuse under the Creative Commons BY-SA 3.0 Unported license (https://creativecommons.org/licenses/by-sa/3.0/). Crocodylian skull silhouettes in Figs. 1 and 3 are original creations. Map in Fig. 4 was modified after^118^. All other elements presented in Figs. 1 to 4 are original creations^119^.

### Reporting summary

Further information on research design is available in the Nature Portfolio Reporting Summary linked to this article.

## Supplementary information

Supplementary information

Description of Additional Supplementary Materials

Supplementary Data 1

Supplementary Data 2

Reporting Summary

## Data Availability

All supporting data, supplementary information and supplementary data are available in the following open access repository (Figshare): 10.6084/m9.figshare.27901317.
