# Supplementary material for: Expanded phylogeny elucidates Deinosuchus relationships, crocodylian osmoregulation and body-size evolution
Source: Commun Biol. 2025 Apr 23;8:611. doi: 10.1038/s42003-025-07653-4 (PMC12018936; doi:10.1038/s42003-025-07653-4)
Supplement: Supplementary file 1 — Supplementary information [file 42003_2025_7653_MOESM1_ESM.pdf]

SUPPLEMENTARY INFORMATION

Jules D. WALTER[1,2*], Tobias MASSONNE[2], Ana Laura S. PAIVA[3], Jérémy MARTIN[4], Massimo DELFINO[1,5], Márton RABI[2*]

** corresponding authors*

[1] Dipartimento di Scienze della Terra, Università di Torino, Via Valperga Caluso 35, I-10125 Torino, Italy; julesdenis.walter@unito.it

[2] Department of Geosciences, Eberhard-Karls-Universität Tübingen, Hölderlinstraße 12, D-72076 Tübingen, Germany; marton.rabi@uni-tuebingen.de

[3] Laboratório de Paleontologia, Faculdade de Filosofia Ciências e Letras de Ribeirão Preto, Universidade de São Paulo, Ribeirão Preto, São Paulo, Brazil

[4] Université de Lyon, Université Claude Bernard Lyon 1, ENS de Lyon, CNRS, UMR 5276 Laboratoire de Géologie de Lyon: Terre, Planètes, Environnement, F-69622, 2 rue Dubois, Lyon, Villeurbanne, France

[5] Institut Català de Paleontologia Miquel Crusafont, Universitat Autònoma de Barcelona, Edifici ICTA/ICP, c/ Columnes s/n, Campus de la UAB, E-08193 Cerdanyola del Vallès, Barcelona, Spain

TABLE OF CONTENTS

1. CHANGES APPLIED TO THE DATASET OF MASSONNE ET AL. (2019)....2
 1.1 List of taxa added to the dataset of Massonne et al. (2019) 2
 1.2 List of omitted taxa....3
 1.3 List of updates and modifications of scores....4
 1.4 Deleted characters....13
2. ADDITIONAL DATA, RESULTS AND DISCUSSION OF PHYLOGENETIC ANALYSES..15
 2.1 Phylogenetic analysis of the modified Massonne et al. (2019) dataset....15
  Figure S1....16
  List of synapomorphies recovered for Alligatoroidea 17
  List of synapomorphies recovered for Crocodylia....17
  List of synapomorphies for Alligatoroidea recovered in Cossette & Brochu (2020) 18
 2.2 Phylogenetic analyses using a modified version of the Rio & Mannion (2021) dataset 18
  Results....21
  Figure S2....22
  Figure S3....24

Discussion....................24
3. SOURCES FOR TAXA....................25
Table S1. List of extant and extinct gigantic crocodyliforms (≥ 7m in total length)....................32
4. LIST OF MORPHOLOGICAL CHARACTERS....................32
5. REFERENCES....................48

The supplementary information (present document) is the main additional material file associated with the manuscript and phylogenetic analyses.

The folder ‘Supplementary Data 1’ contains the character-taxon dataset used for the phylogenetic analyses as well as the TNT files.

The folder ‘Supplementary Data 2’ contains all the primary data, code and files used to determine the body size estimates (folders 1. and 2.), along with the raw data and results (folder 3.).

All our data is accessible under the following Figshare repository link: https://doi.org/10.6084/m9.figshare.27901317.v1

1. CHANGES APPLIED TO THE DATASET OF MASSONNE ET AL. (2019)

For our phylogenetic analysis, we modified the dataset of Massonne et al. (2019), which in turn is based on Brochu (1999, 2004), Brochu & Storrs (2012), Wang, Sullivan & Liu (2016), Cossette & Brochu (2018), and Li, Wu & Rufolo (2019). In order to update and increase the taxon-character sample of Massonne et al. (2019), we combined it with the datasets of Gismondi et al. (2015), Bona et al. (2018), Cossette (2021), Shan et al. (2021), Stocker et al. (2021), and Walter et al. (2022), including the addition of 20 characters, 14 character states, and 17 taxa. We furthermore added the taxa *Diplocynodon remensis* and *Borealosuchus griffithi* and removed four characters due to irreproducibility, intraspecific variability or redundancy with the added character states. The complete list of characters is available in part [4] of the present document. The expanded dataset now includes a total of 219 characters and 128 taxa. See Supplementary Data 1 folder for nexus and tnt files and part [2] of the present document and Methods of the main manuscript for details of the analysis.

1.1 List of taxa added to the dataset of Massonne et al. (2019)

The following 19 taxa were added to the parent dataset (see [3] for sources):

- *Albertochampsa langstoni* Erickson, 1972
- *Borealosuchus griffithi* Wu, 2001

- *Bottosaurus fustidens* Cossette, 2021
- *Caiman wannlangstoni* Salas-Gismondi, Flynn, Baby, Tejada-Lara, Wesselingh & Antoine, 2015
- *Chinatichampsus wilsonorum* Stocker, Brochu & Kirk, 2021
- *Deinosuchus schwimmeri* Cossette & Brochu, 2020
- *Diplocynodon remensis* Martin, Smith, de Lapparent de Broin, Escuillié & Delfino, 2014b
- *Dongnanosuchus hsui* Shan, Wu, Sato, Cheng & Rufolo, 2021 (replacing the incomplete taxon "Maoming alligatoroid"; Skutschas et al. 2014, Massonne et al. 2019)
- *Eocaiman itaboraiensis* Pinheiro, Fortier, Pol, Campos & Bergqvist, 2013
- *Eocaiman palaeocenicus* Bona, 2007
- *Gnatusuchus pebasensis* Salas-Gismondi, Flynn, Baby, Tejada-Lara, Wesselingh & Antoine, 2015
- *Kuttanacaiman iquitosensis* Salas-Gismondi, Flynn, Baby, Tejada-Lara, Wesselingh & Antoine, 2015
- *Caiman brevirostris* Souza Filho, 1987
- *Mourasuchus amazonensis* Price, 1964
- *Mourasuchus arendsi* Bocquentin Villanueva, 1984
- *Necrosuchus ionensis* Simpson, 1937
- *Protocaiman peligrensis* Bona, Ezcurra, Barrios & Blanco, 2018
- *Purussaurus brasiliensis* Barbosa-Rodrigues, 1892
- *Purussaurus mirandai* Aguilera, Riff & Bocquentin-Villanueva, 2006

1.2 List of omitted taxa

A total of 4 taxa were removed from the dataset:

- *Asiatosuchus nanlingensis* Young, 1964

This taxon was omitted due to its incompleteness (Shan et al., 2021).

- *Culebrasuchus mesoamericanus* Hastings et al., 2013

  This taxon was omitted due to its poor preservation and further studies are required before it can be reintroduced into the phylogeny (see Stocker et al., 2021).

- *Melanosuchus fisheri* Medina, 1976.

  The taxon was considered to be a *nomen dubium* (Bona et al., 2017).

- *Orthogenysuchus olseni*, Mook 1924

  This taxon was omitted due to its poor preservation and need for osteological revision (see Stocker et al., 2021 and Walter et al., 2022).

1.3 List of updates and modifications of scores

39. Number of contiguous dorsal osteoderm rows:

Diplocynodon hantoniensis: 1 > ?

We are unaware of a complete dorsal shield preserved for this species. The character is therefore rescored as (?).

46. Alveoli size of dentary teeth: 3rd and 4th same size and confluent (0), 4th larger than 3rd and separate (1), 3rd and 4th nearly same size and separate (2).

Diplocynodon deponiae: ? > 0

Diplocynodon deponiae, as all other *Diplocynodon* species, has confluent 3rd and 4th dentary alveoli (Delfino & Smith, 2012, Fig.4). The character is therefore rescored as (0).

47. Anterior dentary teeth strongly procumbent (0) or project anterodorsally (1).

Eocaiman cavernensis: 1 > 0

The anterior dentary teeth are procumbent in the only known specimen (AMNH FARB 3158). This is also apparent in Godoy et al. (2021 figs. 1, 8) and the condition is not different

from other taxa scored for this state in the dataset. To make the scorings consistent, we therefore rescore *E. cavernensis* as (0).

49. Dentary gently curved (0), deeply curved (1), or linear (2) between fourth and tenth alveoli.

Dongnanosuchus hsui: 0 > 1

The lower jaw of *D. hsui* is preserved in occlusion. In Shan et al. (2021 fig. 6 1), the curvature nevertheless looks deeper than in *J. nankangensis* (state 0 - Li et al., 2019 fig. 6 c), and more similar to the stronger curvature of *O. naduongensis* (state 1 - Massonne et al., 2019 fig. 8a). The character is therefore rescored as (1).

50. Largest dentary alveolus immediately caudal to fourth is (0) 13 or 14, (1) between 11 and 14 and a series behind it, (2) 11 or 12, (3) no differentiation, (4) behind 14, (5) 10.

Bottosaurus harlani: 1 > 2

Massonne et al. (2019) changed the scoring of this species from 2 to 1 but this was apparently a misinterpretation.Based on Cossette & Brochu (2018 fig. 5) and personal observation, the condition is clearly state (2).

58. Process of splenial separates angular and coronoid (0) or no splenial process between angular and coronoid (1).

Globidentosuchus brachyrostris: ? > 1

No splenial process is visible between the angular and coronoid (Scheyer & Delfino, 2016 fig. 9). The character is therefore rescored as (1).

59. Angular-surangular suture contacts external mandibular fenestra at posterior angle at maturity (0) or passes broadly along ventral margin of external mandibular fenestra late in ontogeny (1).

Globidentosuchus brachyrostris: 1 > 0

The angular-surangular suture reaches the external mandibular fenestra posteriorly (Scheyer & Delfino, 2016 fig. 9). The character is therefore rescored as (0).

60. Surangular, relative length of the anterior processes: equal (0); subequal (1).

Borealosuchus formidabilis: ? > 1

Diplocynodon deponiae: 1 > ?

Diplocynodon tormis: 1 > ?

Borealosuchus formidabilis is here scored (1) following Rio and Mannion (2021; in Appendix 2, Fig. 107). In *Diplocynodon deponiae* and *Diplocynodon tormis*, the relative extent of the surangular processes are incomplete (pers. obs.; Buscalioni et al., 1992; Delfino & Smith, 2012).

62. External mandibular fenestra absent (0) or present as narrow slit, no discrete fenestral concavity on angular dorsal margin (1) or present with discrete concavity on angular dorsal margin (2) or present and very large; most of foramen intermandibularis caudalis visible in lateral view (3).

Orientalosuchus naduongensis: 2 > 1

Orientalosuchus naduongensis has a very small external mandibular fenestra, noticeably reduced compared to closely related taxa. To account for this obvious difference, the character is rescored as (1).

Globidentosuchus brachyrostris: 1 > 3

The foramen intermandibularis caudalis is apparent in lateral view in Scheyer & Delfino (2016 fig. 9). The character is therefore rescored as (3).

65. Surangular-angular suture lingually meets articular at ventral tip (0) or dorsal to tip (1).

Bottosaurus harlani: ? > 1

We here follow the scoring of Cossette & Brochu (2018).

66. Surangular continues to dorsal tip of lateral wall of glenoid fossa (0) or truncated and not continuing dorsally (1).

Bottosaurus fustidens: 0 > ?

The limited preservation of the relevant specimen (TMM 40148-7; Cossette, 2021 fig. 5) does not allow assessment of the full extent of the surangular-articular suture. The glenoid fossa does not seem complete enough to detect a possible truncation. We here conservatively rescore the character as (?).

78. Teeth and alveoli of maxilla and/or dentary circular in cross-section (0), or posterior teeth laterally compressed (1), or all teeth compressed (2).

Dongnanosuchus hsuii: 0 > 1

There does not seem to be a difference in the posterior tooth morphology between *D. hsui* and *Orientalosuchus naduongensis* (scored (1)). In *D. hsui* (Shan et al., 2021 fig. 6.6), the posterior teeth are clearly longer than wide (laterally compressed). The character is therefore rescored as (1).

84. External naris (0) opens flush with dorsal surface of premaxillae or (1) circumscribed by thin crest.

Dongnanosuchus hsui: 1 > 0

The condition in *D. hsui* is not comparable to the thin crest around the naris as present in e.g. *Tsoabichi greenriverensis* (Brochu, 2010 fig. 3c) and appears as a thinner bulge instead, similar to *O. naduongensis* (without a notch posterolateral to the naris). The character is therefore rescored as (0).

Eocaiman cavernensis: 0 > ?

The external naris is not preserved in the only specimen unambiguously referred to this species (pers. obs.; Godoy et al., 2021 fig. 1). The character is therefore rescored as (?).

88. Incisive foramen completely situated far from premaxillary tooth row, at the level of the second or third alveolus (0) or abuts premaxillary tooth row (1) or projects between first premaxillary teeth (2).

Brachychampsa montana: ? > 0

The incisive foramen is located away from the toothrow (pers. obs.; Norell et al., 1994, fig. 1) as in other taxa scored for the corresponding state. The character is therefore rescored as (0).

Brachychampsa sealeyi: ? > 0

The incisive foramen is located away from the toothrow (Williamson, 1996, fig. 2) as in other taxa scored for the corresponding state. The character is therefore rescored as (0).

Albertochampsa langstoni: ? > 1

The incisive foramen abuts the toothrow (Erickson, 1972, fig. 2) as in other taxa scored for the corresponding state. The character is therefore rescored as (1).

We follow Stocker et al. (2021, ch. 89) who rescored this character due to what may have been a transcription error in one of their parent datasets.

90. Dentary tooth 4 occludes in notch between premaxilla and maxilla early in ontogeny (0) or occludes in a pit between premaxilla and maxilla; no notch early in ontogeny (1).

The definition of character 90 specifies early ontogenetic stage since a pit may develop into a notch during ontogeny in some taxa. Massonne et al. (2019) noticed that several taxa with a premaxillary-maxillary notch in the adult stage was scored for this character, even though the early ontogenetic stage is not actually known (in the absence of subadult specimens). In order to fix this, Massonne et al. (2019) added a new character (ch. 194 of the present study) that only considers the adult condition. To avoid double weighting of the same morphology, Massonne et al. (2019) rescored all taxa with no known subadult specimens as unknown for character 90. For few taxa, however, Massonne et al. (2019)

incorrectly assumed that the juvenile condition was known, which we here correct by updating the following taxa:

Acynodon iberoccitanus: 1 > ?

Allodaposuchus precedens: 1 > ?

92. Largest maxillary alveolus is 3 (0), 5 (1), 4 (2), 4 and 5 are same size (3), 6 (4), or maxillary teeth homodont (5), or maxillary alveoli gradually increase in diameter posteriorly toward penultimate alveolus (6).

Bottosaurus fustidens: 2 > ?

In the maxillae referred to this species (TMM 40148-7; Cossette, 2021; fig. 4 and TMM 41336-16; Cossette, 2021; fig. 7B), the size of the 5th alveolus is not fully discernible, thereby making an adequate comparison to that of the 4th impossible. The character is therefore rescored as (?).

97. Antorbital fenestra present (0) or absent (1).

Globidentosuchus brachyrostris: ? > 1

An antorbital fenestra is absent in this taxon (Hastings et al., 2016, fig. 4).

112. Anterior face of palatine process rounded or pointed anteriorly (0) or notched anteriorly (1).

Eocaiman cavernensis: ? > 0

The anterior palatine process is pointed in AMNH FARB 3158 (pers. obs.; Godoy et al., 2021, fig. 4) and instead of medially, it is laterally notched. The morphology differsfrom the notched condition of e.g. *Paleosuchus palpebrosus*. The character is therefore rescored as (0).

115. Palatine process generally broad anteriorly (0) or in form of a thin wedge (1).

Chinatichampsus wilsonorum: 1 > 0

Based on Stocker et al. (2021, fig. 5) the anterior palatine process is round and broad instead of forming a thin wedge. We assume this was a typographical error in Stocker et al (2021). The character is therefore rescored as (0).

119. Lateral edges of palatines parallel posteriorly (0) or flare posteriorly, producing shelf (1).

Diplocynodon darwini: 0 > 1
Diplocynodon deponiae: 0 > 1
Diplocynodon hantoniensis: 0 > 1
Diplocynodon muelleri: 0 > 1
Diplocynodon ratelii: 0 > 1

In Massonne et al. (2019) the definition of ch. 119 was misinterpreted and this error is corrected here.

124. Internal choana not septate (0) or with septum that remains recessed within choana (1) or with septum that projects out of choana (2).

We follow Stocker et al. (2021, ch. 125) who rescored this character due to what may have been a transcription error in one of their parent datasets.

128. Prefrontals separated by the frontals and nasals, anterior process of frontal extending far anterior to the anterior margin of the orbit (0) prefrontals separated by the frontal and nasals, anterior process of frontal around the same level or posterior to the anterior margin of the orbit (1) or prefrontals meet medially, anterior process of frontal around the same level or posterior to the anterior margin of the orbit (2).

Albertochampsa langstoni: ? > 1

The anterior process of the frontal is in line with the orbit (Erickson; 1972 fig. 1).

130. Anterior tip of frontal (0) forms simple acute point or (1) forms broad, complex sutural contact with the nasals.

Deinosuchus schwimmeri: 2 > 1

This character was previously scored with state (2) by Shan et al. (2021), but the character includes two states only. The character is therefore rescored as (1).

139. Quadratojugal spine presence: prominent (0); greatly reduced or absent (1).

Diplocynodon tormis: 1 > ?

Diplocynodon tormis does not preserve a complete lower temporal fenestra, lacking most elements posterior to the jugal in the most complete specimen (IPS-9001, pers. obs.). Other referred specimens by Buscalioni et al. (1992) consist of skull elements not pertaining to or not preserving the area of interest (see also Serrano-Martinez et al., 2019 for a description of another specimen, STUS-344). The authors also mentioned disarticulated material attributed to *Diplocynodon tormis* but without further description, figures or inventory numbers. Since the score appears to be irreproducible, we rescore this character as unknown (?).

140. Quadratojugal spine position: low, near posterior angle of fenestra (0); high, between posterior and superior angles of fenestra (1).

Diplocynodon tormis: 1 > ?

See justification under ch. 139.

148. Caudal margin of otic aperture not defined and gradually merging into the exoccipital (0) or smooth and continuous with the paraoccipital process (1) or caudal margin of otic aperture inset (2).

Diplocynodon tormis: 2 > ?

See justification under ch. 139.

150. Frontoparietal suture concavoconvex (0) or linear (1) between supratemporal fenestrae.

Globidentosuchus brachyrostris: 1 > ?

In Hastings et al. (2016, fig. 4), the frontoparietal suture cannot be clearly followed. The scores of Massonne et al. (2019) and Walter et al. (2022) are contradicting. We here therefore prefer caution over the scoring of this character and rescore it as unknown (?).

163. Prootic on external wall of braincase is extensively exposed (0); largely obscured by the quadrate and laterosphenoids externally (1).

Eoalligator chunyii: 1 > ?

Specimens of *Eoalligator chunyii* do not preserve the prootic (Wang, 2016; Wu et al. 2018). The extent of its exposure relative to the quadrate and laterosphenoid therefore cannot be assessed.

196. If largest dentary alveolus is between 11th and 14th and a series behind it, is it the (0) 11th, (1) 12th, or (2) 13th or 14th.

Bottosaurus harlani: 1 > (-)

After rescoring character 50 for this taxon (see above), character 196 is no longer applicable. The character is therefore scored as (-).

202. Ventral premaxilla-maxilla suture short and ends posteriorly before the 3rd maxillary alveoli (0) or elongated and extends or exceeds the 3rd maxillary alveoli (1). [added from Stocker et al. (2021)]

Brachychampsa montana: 0 > 1

In *B. montana* the premaxilla-maxilla suture is strongly bowed posteriorly due to the large incisive foramen. The suture reaches the level of the fourth maxillary tooth (pers. obs.; Gilmore, 1911 pl. 27; Norell et al. 1994 fig. 1). The character is therefore scored as (1).

<u>204</u>. Lateral edge of the skull table at the level of the postorbital-squamosal suture situated laterally or at the same level as (0), or medially to (1) the quadrate condyle in dorsal view at maturity. [added from Stocker et al. (2021)]

Boverisuchus vorax: 1 > 0

In *B. vorax*, the lateral edge of the skull table at the level of the postorbital-squamosal suture reaches the level of the medial quadrate condyle (Langston, 1975 fig.1; Brochu, 2012 fig. 3). The character is therefore scored as (0).

<u>207</u>. Interorbital bridge narrower to equivalent (0), or broader (1) than the width of the orbit. [added from Stocker et al. (2021)]

Boverisuchus vorax: 0 > 1

In *B. vorax*, the interorbital bridge is wide, broader than the width of the orbit (pers. obs.; Langston, 1975 fig.1; Brochu, 2012 fig. 3). The character is therefore scored as (1).

<u>215</u>. Dentary teeth series behind alveoli 12-13 are pointed to slightly blunt (0); globular, different in size among them (1); globular, at least four subequal in size (2), molariform multicusped (3) or absent (4). [added from Stocker et al. (2021)]

Bottosaurus harlani: 1 > 0

In *B. harlani*, posterior teeth behind the 12th are slightly blunt rather than being globular (Cossette & Brochu, 2018 fig. 5) and resemble the corresponding teeth of e.g. *O. naduongensis* more than the condition of e.g. *Ceratosuchus burdohsi* (state 1). In order to remain consistent with the scores and morphology of other taxa, we rescore *B. harlani* as (0).

<u>1.4 Deleted characters</u>

Character 1 of Massonne et al. (2019)

Ventral tubercle of proatlas: more than one-half (0), or no more than one-half (1) the width of the dorsal crest. [OMITTED]

The definition of this character is imprecise regarding the angle of view necessary to score the morphology of the tubercles. A few fossil taxa were scored for this character in the parent dataset, but their scoring was irreproducible. Moreover, Sookias (2020; supplementary)

evaluated the character as non-robust in his review of crocodylian morphological characters, based on a different dataset using the same version of the character (Narváez et al., 2015). The author specifies that the variation indicated by the scorings could not be observed for extant taxa. Given the uncertainty of the morphological variation and irreproducibility of the scoring distribution, we omit this character from the present analysis.

Character 194 of Massonne et al. (2019)

Nasal bone does (0) or does not (1) reach to the height of the orbita. [OMITTED]

This character was introduced in Massonne et al. (2019) and by updating ch.128 of the present dataset with an additional state (after Salas-Gismondi et al. 2015), this morphology is already included and makes ch. 194 redundant.

Character 197 of Massonne et al. (2019)

Sutural contact of the exoccipitals dorsal to the foramen magnum (0) long, at least half the height of the foramen magnum, (1) short, shorter than half the height of the foramen magnum, or (2) no sutural contact between the exoccipitals. [OMITTED]

This character was introduced in Massonne et al. (2019). In occipital view, the supraoccipital is generally triangular but its ventral extension differs among taxa. In e.g. *Acynodon iberoccitanus* Buscalioni, Ortega & Vasse 1997, the supraoccipital nearly prevents contact between the exoccipitals (Martin, 2007 fig. 2), whereas in *Leidyosuchus canadensis* Lambe, 1907, the ventral supraoccipital process is short, allowing a long contact between the exoccipitals (Wu et al. 2001, fig. 3). However, we find that in most taxa, the condition is intermediate or the variation is continuous. In addition, fossil skulls are commonly crushed. We therefore omit this character from the present analysis.

Character 199 of Massonne et al. (2019)

Intersupratemporal bar (0) as or near as broad as the supratemporal fenestra, (1) at least twice as broad as the supratemporal fenestra, (2) around half the broadness of the supratemporal fenestra or (3) constricted, less than half the broadness of the supratemporal fenestra. [OMITTED]

This character was introduced in Massonne et al. (2019). While the extreme conditions are straightforward (broad bar in caimanines with small or nearly closed supratemporal fenestrae versus a constricted bar in gavialines with large fenestrae), variation is continuous in most other taxa. Furthermore, the character is partly correlating with ch. 209.

2. ADDITIONAL DATA, RESULTS AND DISCUSSION OF PHYLOGENETIC ANALYSES

2.1 Phylogenetic analysis of the modified Massonne et al. (2019) dataset

The character-taxon dataset (nexus and tnt files) is in Supplementary Data 1. The complete list of characters is provided in section [4] of the present document. Strict consensus tree is provided in Figure S1, together with the molecular scaffold topology implemented.

Excluding the two Paleocene taxa *Borealosuchus griffithi* and *Diplocynodon remensis*, and deactivating the constraints recovers a few trees with *Deinosuchus* spp. as the earliest branching alligatoroid.

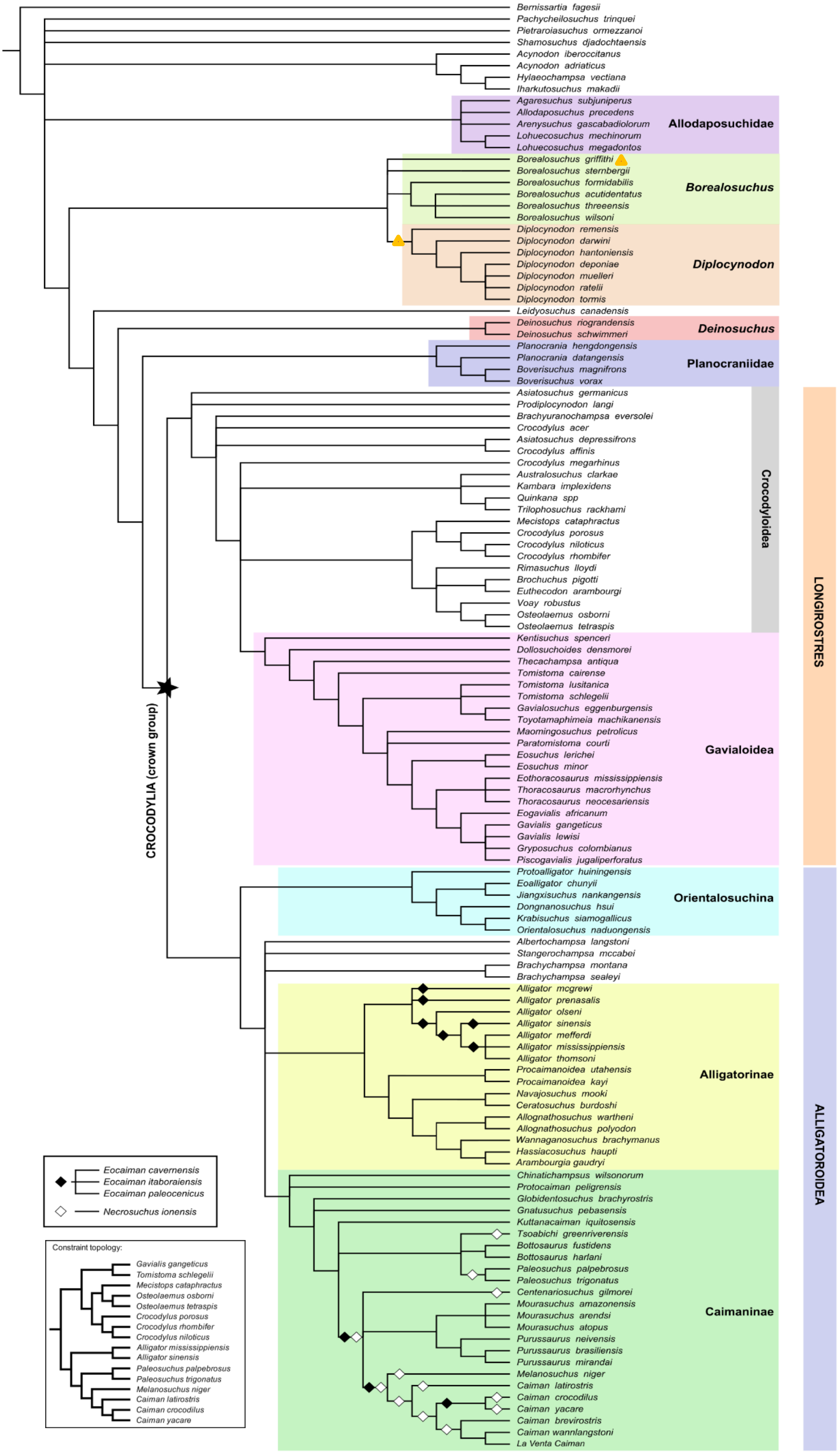

Figure S1.
Pruned strict consensus of 506 most parsimonious trees (tree length = 1185) from the parsimony analysis of the modified Massonne et al. (2019) dataset employing a molecular backbone constraint following Darlim et al. (2022) and Oaks (2011), indicated on the bottom left. *Borealosuchus griffithi* has two alternative positions, either as sister

to *Diplocynodon* spp. or an early diverging placement within *Borealosuchus* spp. (orange triangle; see Supplementary Data 1, “Walter et al_[TNT]” for the tnt file). Alternative positions of pruned taxa (*Eocaiman* spp. and *Necrosuchus ioensis*) are furthermore indicated.

List of synapomorphies recovered for Alligatoroidea

4. Atlas intercentrum shape: wedge-shaped (0); or plate-shaped (1).
0 > 1

38. Dorsal midline osteoderms shape: rectangular (0); or nearly square (1).
0 > 1

50. Largest dentary alveolus caudal to fourth: 13 or 14 (0); 13 and 14 and series behind it (1); 11 or 12 (2); no differentiation (3); behind 14 (4); 10 (5); 11 and series behind it (6); or 12 and series behind it (7).
2 > 1

58. Splenial process: separates angular and coronoid (0); or no process between angular and coronoid (1).
0 > 1

121. Choana projecting at maturity: posteroventrally (0); or anteroventrally (1).
0 > 1

131. Ectopterygoid extension at postorbital bar: extends along medial face of postorbital bar (0); or stops abruptly ventral to postorbital bar (1).
0 > 1

139. Quadratojugal spine appearance: prominent at maturity (0); or greatly reduced or absent at maturity (1).
0 > 1

List of synapomorphies recovered for Crocodylia

17. Third cervical vertebra (first postaxial) with prominent hypapophysis (0) or lacks prominent hypapophysis (1).
1 > 0

26. Proximal edge of deltopectoral crest emerges smoothly from proximal end of humerus and is not obviously concave (0) or emerges abruptly from proximal end of humerus and is obviously concave (1).
0 > 1

80. Naris projects anterodorsally (0) or dorsally (1) or posterodorsally (2). [modified by Cossette & Brochu (2020)]:
0 > 1

88. Incisive foramen completely situated far from premaxillary tooth row, at the level of the second or third alveolus (0) or abuts premaxillary tooth row (1) or projects between first premaxillary teeth (2).
0 > 1

92. Largest maxillary alveolus is 3 (0), 5 (1), 4 (2), 4 and 5 are same size (3), 6 (4), or maxillary teeth homodont (5), or maxillary alveoli gradually increase in diameter posteriorly toward penultimate alveolus (6).
3 > 1

149. Frontoparietal suture deeply within supratemporal fenestra; frontal prevents broad contact between postorbital and parietal (0) or suture makes modest entry into supratemporal fenestra at maturity; postorbital and parietal in broad contact (1) or suture on skull table entirely (2).
0 > 1

List of synapomorphies for Alligatoroidea recovered in Cossette & Brochu (2020)

60 (1): Anterior processes of the surangular are equal to subequal.

69 (1): Foramen aerum of articular set in from the margin of retroarticular process.

91 (0): All dentary teeth occlude lingual to maxillary teeth.

130 (1): Anterior tip of frontal forms broad, complex sutural contact with the nasals.

140 (1): The quadratojugal spine is high, between posterior and superior angles of infratemporal fenestra.

176 (1): Quadrate foramen aëreum on dorsal surface.

2.2 Phylogenetic analyses using a modified version of the Rio & Mannion (2021) dataset

In order to test the phylogenetic position of *Deinosuchus* and *Diplocynodon* spp., we performed a parsimony analysis on an alternative dataset using a modified version of Rio and Mannion (2021) [Supplementary Data 2, “Walter-RM” files]. Modifications are detailed below.

We added the following taxa:

Deinosuchus riograndensis Colbert & Bird, 1954

Borealosuchus griffithi Wu, 2001

We modified the scores of the following taxa:

32. Rostral ornamentation, morphology of the transverse orbital ridge (i.e. spectacle): low, lacking a posterior fossa (0); tall, with deep posterior fossa (1).

Diplocynodon remensis: 1 > 0

The ridge is low in *Diplocynodon remensis*, preserved in both the holotype MHNH F BR 4020 and specimen CE0001.

87. Supratemporal fenestra, posterior wall: quadrate forms entire ventral margin of orbitotemporal canal (no parietal-squamosal contact) (0); quadrate partially forms ventral margin of orbitotemporal canal (parietal and squamosal narrowly separated) (1); quadrate excluded from ventral margin of orbitotemporal canal (parietal and squamosal in contact) (2).

Diplocynodon hantoniensis: 1 > (?)

In the juvenile specimen NHMUK OR 25170a, the parietal and squamosal are in contact in the posterior wall of the supratemporal fenestra (2) in contrast to the (1) scoring of Rio & Mannion (2021). Moreover, the left and right squamosal-quadrate sutures in NHMUK OR30393 (Rio et al., 2020; fig. 30) appear different on each side, and might represent independent cracks. In this case, the parietal-quadrate sutures indicated on the figure may actually correspond to the squamosal-parietal contact.

Diplocynodon darwini: 1 > (?)

Direct observation of a large sample of specimens, including those cited in Rio & Mannion (2021), reveals that the material referable to this species is dorsoventrally compressed and we were unable to reproduce the previous scoring of this character. We here conservatively score this taxon as unknown (?).

142. Premaxilla, posterior extent on palate, relative to number of maxillary alveoli, in ventral view: 0 (0); 1 (1); 2 (2); 3 (3); 4 (4); 5 or more (5).

Diplocynodon darwini: ? > (0)

Based on specimen GMH XXXVI 524 1966, the premaxilla does not even extend up to the 1st maxillary alveolus.

241. Surangular, relative length of the anterior processes: unequal, ventral process <75% anteroposterior length of dorsal process (measured from surangular foramen) (0); sub-equal, ventral process >= 75% length of dorsal process (1).

Diplocynodon hantoniensis: 1 > 0

This scoring was likely erroneously entered in the previous version of the dataset, as state 1 is clearly recognisable in fig. 9D from Rio et al. (2020).

Diplocynodon muelleri: 1 > (?)

Piras & Buscalioni (2006) described this morphology as follows "In its anterior dorsal suture with the dentary, the surangular probably bears two subequal anterior processes. This feature is also seen in the holotype and in a specimen from the Tàrrega Museum". Direct observation of the holotype and paratype however did not allow assessment of this feature, as the surangular is fragmentary in NMB-Spa 4., and occlusion of the jaws obscures the morphology in NMB-Spa. 73. The alternative specimen of the Tàrrega Museum is not precisely referred, figured nor described, making the previous scoring irreproducible. We therefore adopt a more cautious scoring, here (?).

Diplocynodon ratelii: 1 > 0

The processes are unequal in the specimen MNHN SG 599, we therefore rescore the character as (0).

Diplocynodon remensis: 1 > 0

Based on Martin et al. (2014, fig.5; specimen CE0001), the processes are unequal. We therefore rescore the character as (0).

248. Articular, position of foramen aëreum: at medial margin of retroarticular process (0); inset from medial margin of retroarticular process (1).

Borealosuchus formidabilis: 0 > 1

Erickson (1976) describes the alligatoroid condition observed on the single specimen preserving this feature (SMM P74.24.6).

Results

Parsimony analyses of the modified Rio & Mannion (2021) dataset were ran in TNT 1.6. We performed a first round of Traditional Search using 1000 replicates of Wagner trees, followed by a second round of Tree Bisection Reconnection (TBR) using trees saved from the first round, with 10 saves per replication.

Equal weighting parsimony analyses in TNT are as follows:

Continuous + discrete dataset (equivalent to analysis 1.1 in Rio & Mannion, 2021)

- 8 MPTs were recovered from the analysis, with a tree length of 225,845.239 (Fig. S2).
- *Diplocynodon* and *Deinosuchus* are placed at the base of Alligatoroidea, but have a poor stratigraphic fit.
- *Diplocynodon* is monophyletic under this topology.
- *Leidyosuchus* is the earliest-branching alligatoroid.
- *Borealosuchus* is monophyletic under this topology. *Planocrania hengdongensis* is sister to the latter clade.
- Planocraniids are not recovered in Alligatoroidea under this topology, in contrast to Rio & Mannion (2021).
- 8 synapomorphies are recovered for Alligatoroidea
    - 1: 1.382-1.608 > 1.614–1.710 - Skull proportions, ratio of mediolateral rostrum width at the level of the anterior orbital margin, to mediolateral width across anterior margin of the cranial table.
    - 104: 0 > 1 - Quadratojugal, position of spina quadratojugalis: high, between posterior and dorsal angles of infratemporal fenestra.
    - 117: 0 > 1 - Quadrate, foramen aërum position on posterior quadrate ramus: on dorsal surface.
    - 181: 2 > 0 - Ectopterygoid, morphology of posterior process on the medial jugal surface: acute, extends beyond level of posterior margin of postorbital bar.
    - 195: 0 > 2 - Choanae, ornamentation of margins: elevated forming a wall which extends to the anterolateral (but not anterior) margins of the choanae.
    - 217: 1 > 0 - Dentary, alveoli 3 and 4: confluent.
    - 221: 1 > 0 - Mandibular symphysis, posterior extent, adjacent to number of full dentary alveoli: < 6.
    - 248: 0 > 1 - Articular, position of foramen aërum: inset from medial margin of retroarticular process.

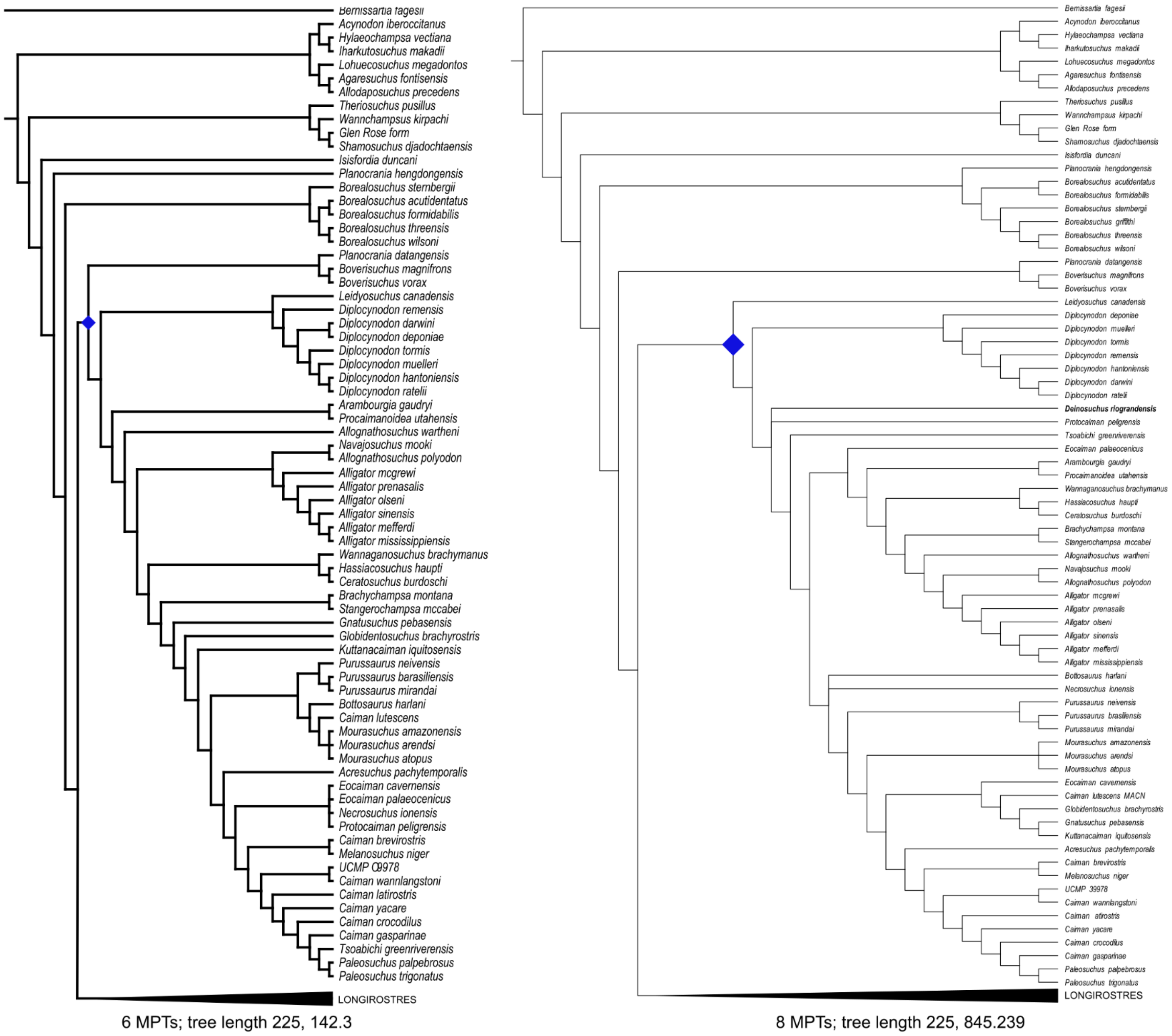

Figure S2.
Strict consensus tree of the Analysis 1.1 of Rio & Mannion (2021) continuous + discrete character dataset (left) and the modified dataset of Rio & Mannion (2021) used in the present study (right). Blue diamond denotes the node Alligatoroidea.

Re-discretised + discrete dataset (equivalent to analysis 2.1 in Rio & Mannion, 2021):

- number of MPTs > 99999, tree length of 2270 steps (Fig. S3).
- Similarly to the continuous + discrete version of the dataset, *Diplocynodon* and *Deinosuchus* are placed at the base of Alligatoroidea, contra Rio & Mannion (2021)

result which recovered *Diplocynodon*, *Leidyosuchus* and *Borealosuchus* as early-branching Longirostres.

- *Diplocynodon* is monophyletic under this topology.
- *Deinosuchus* is recovered as a basal alligatoroid, placed one step crownward to *Diplocynodon*.
- *Leidyosuchus* is recovered as a ste-crocodylian under this topology. In contrast to Rio & Mannion (2021), *Isisfordia* is not recovered in Alligatoroidea, but forms a sister clade to crown-Crocodylia together with *Leidyosuchus* and other traditional early-branching eusuchians.
- 10 synapomorphies are recovered for Alligatoroidea:
    - 94: 0 > 1 - Orbit, dorsal profile of jugal forming posteroventral margin: posteroventrally sloping, gradually descending into the lower temporal bar.
    - 103: 0 > 1 - Quadratojugal, development of spina quadratojugalis (at maturity): greatly reduced or absent.
    - 104: 0 > 1 - Quadratojugal, position of spina quadratojugalis: high, between posterior and dorsal angles of infratemporal fenestra.
    - 152: 0 > 1 - Occlusion pattern, 4th dentary tooth occludes in a pit between premaxilla and maxilla; no notch early in ontogeny.
    - 180: 0 > 1 - Ectopterygoid, dorsal extent along medial surface of postorbital bar: small, level with or ventral to level of ventral orbital margin.
    - 222: 0 > 2 - Splenial, participation in symphysis: no participation.
    - 267: 0 > 1 - Atlantal rib, dorsal margin shape: with prominent process.
    - 283: 0 > 1 - Cervical rib 8, length in proportion to cervical rib 9: short, equal to or less than half the length of cervical rib 9.
    - 294: 0 > 1 - Caudal vertebrae, articular surfaces of chevrons posterior to the first: completely fused.
    - 330: 0 > 1 - Limb armour: densely covered in well-formed osteoderms.

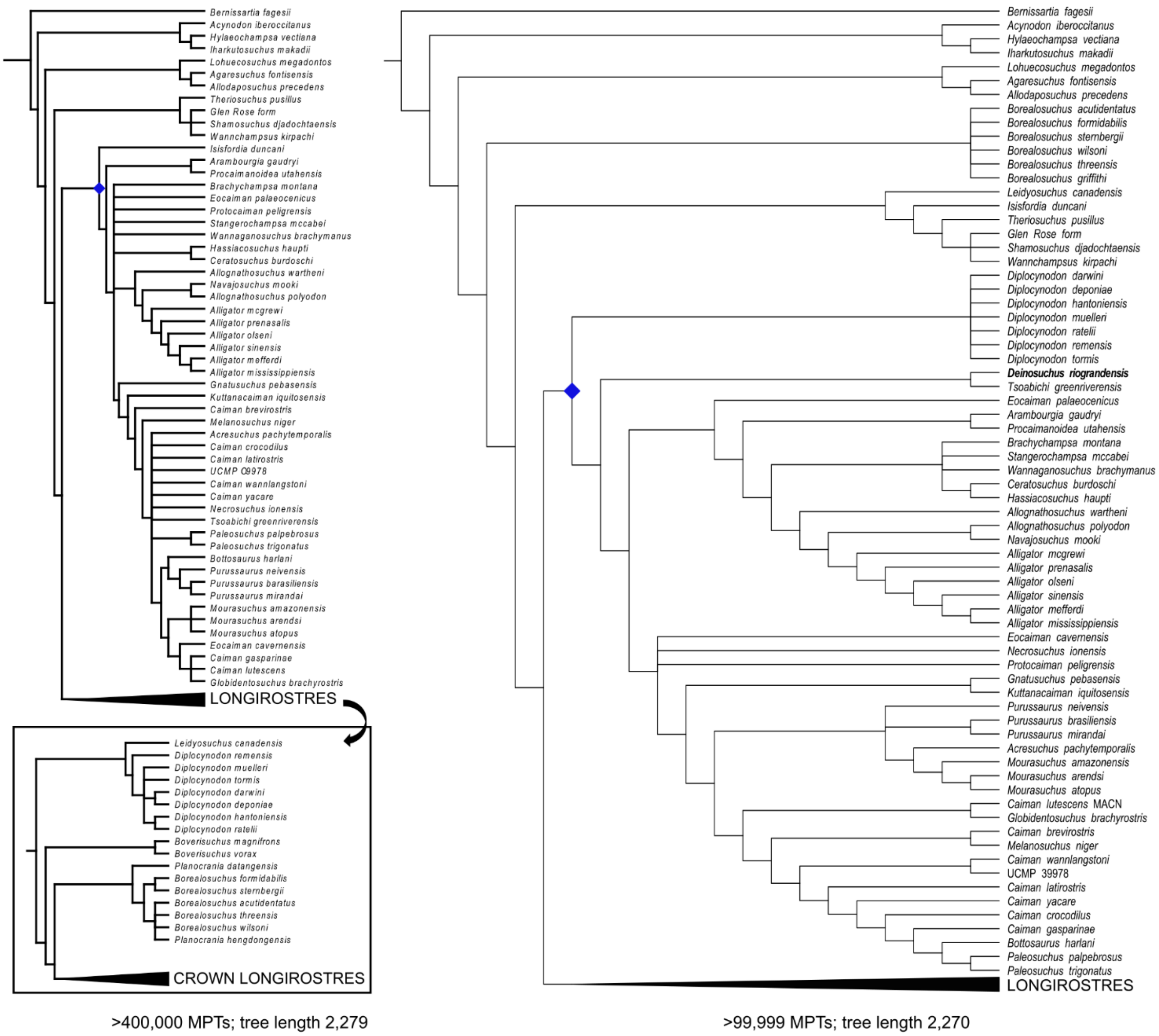

Figure S3.
Strict consensus trees of the redescritised + discrete dataset modified from Rio & Mannion (2021). Left: Analysis 2.1 of Rio & Mannion (2021) parent dataset; Right: this study, modified dataset of Rio & Mannion (2021). Blue diamond denotes the node Alligatoroidea.

Discussion

- *Deinosuchus* is consistently recovered inside Alligatoroidea in the modified Rio & Mannion (2021) dataset, crownward to *Diplocynodon*. This, however, involves a poor stratigraphic fit since representatives of *Deinosuchus* are Campanian and the earliest *Diplocynodon* species is late Paleocene.

- As in previous phylogenies, the stratigraphic fit within *Diplocynodon* is poorly resolved. The oldest species, *Diplocynodon remensis* from the late Paleocene is

recovered in a derived position and *Diplocynodon* is monophyletic. On the other hand, this stratigraphic incongruence is resolved in our preferred and alternative topology using the modified Massonne et al. (2019) dataset (Fig. S1; Figs. 1-2), which furthermore recognizes the unique shared derived traits of *Diplocynodon* and *Borealosuchus*.

- The synapomorphies of Alligatoroidea retrieved by these analyses are mostly ambiguous and are not unique to the clade.

3. SOURCES FOR TAXA

Acynodon adriaticus: Delfino et al., 2008; MCSNT 57248; MCSNT 57032

Acynodon iberoccitanus: Martin, 2007; ACAP-FX1, ACAP-FX2, an ACAP-QR1, ACAP-M1343, ACAP-M260, ACAP-QR7

Agaresuchus subjuniperus: Puértolas-Pascual et al., 2014

Albertochampsa langstoni: Erickson, 1972; Norell et al., 1994;

Alligator mcgrewi: Schmidt, 1941; Brochu, 1999; AMNH FAM

7905, AMNH FAM 8700, AMNH 17090

Alligator mefferdi: Mook, 1941; Brochu, 1999; AMNH 7016

Alligator mississippiensis: O'Brien et al., 2019; Paiva et al., 2022;

Alligator olseni: White, 1942; Brochu, 1999; *Alligator prenasalis*: Brochu, 1999; AMNH 4994, YPM PU

13799, YPM PU 16273, YPM PU 14063

Alligator sinensis: Shan et al., 2013; Iijima et al., 2016; O'Brien et al., 2019; Paiva et al., 2022

Alligator thomsoni: Mook, 1923; Brochu, 1999

Allodaposuchus precedens: Delfino et al., 2008; Martin et al., 2016; PSMUBB V 438; MAFI Ob 3131

Allognathosuchus polyodon: Brochu, 2004b; AMNH 6049

Allognathosuchus wartheni: Brochu, 2004b; YPM PU 16989

Arambourgia gaudryi: Kälin, 1939; MNHN QU17155 (holotype)

Arenysuchus gascabadiolorum: Puértolas-Pascual, 2011

Asiatosuchus germanicus: Berg, 1966; Vasse, 1992; HLMD-Me 5345; GMH-XIV 420; GMH-XIV 4757a; GMH-XIV 4757

Asiatosuchus depressifrons: Delfino & Smith, 2009; Delfino et al., 2017; MNHN G 159, IRScNB IG 9912, IRScNB R 251, IRScNB R 253, IRScNB R 254

Australosuchus clarkae: Willis & Molnar, 1991;

Bernissartia fagesii: Buscalioni & Sanz, 1990; Sookias, 2020; Martin et al., 2020; IRScNB 1538

Borealosuchus acutidentatus: Sternberg, 1932; Lucas & Sullivan, 1986; Brochu, 1997;

Borealosuchus formidabilis: Erickson, 1976; Brochu, 1997; YPM 16242, YPM 16241, YPM 6512; photos from digital collection of SMM

Borealosuchus griffithi: Wu et al., 2001; Lindblad et al., 2022

Borealosuchus sternbergii: Gilmore, 1910; Brochu, 1997; photos from digital collection of USNM

Borealosuchus threensis: Brochu, 2012

Borealosuchus wilsoni: Mook, 1959; Brochu, 1997; Hester, 2018; AMNH 6050, AMNH 7637;

photos from digital collection of USNM

Bottosaurus harlani: Cossette & Brochu, 2018;

Bottosaurus fustidens: Cossette, 2021;

Boverisuchus magnifrons: Rossmann, 1998; Brochu, 2012; all material in GMH, HLMD

Boverisuchus vorax: Langston, 1975; Rossmann, 1998; Brochu, 2012; YPM 249 (holotype),

Brachychampsa montana: Norell et al., 1994; Carpenter & Lindsey, 1980; Brochu, 1999; Sullivan & Lucas, 2003; AMNH 5032 (holotype)

Brachychampsa sealeyi: Williamson, 1996

Brachyuranochampsa eversolei: Zangerl, 1944; Mook, 1962; Brochu, 2000; AMNH FARB 6048

Brochuchus pigotti: Conrad et al., 2013

Caiman brevirostris: Fortier et al., 2014; Scheyer & Delfino, 2016

Caiman crocodilus: O'Brien et al., 2019; Paiva et al., 2022

Caiman latirostris: O'Brien et al., 2019; Paiva et al., 2022

Caiman wannlangstoni: Salas-Gismondi et al., 2015; Scheyer & Delfino, 2016

Caiman yacare: O'Brien et al., 2019; Paiva et al., 2022

Centenariosuchus gilmorei: Hastings et al., 2016; Stocker et al., 2021

Ceratosuchus burdoshi: Bartels, 1983; Brochu, 1999

Chinatichampsus wilsonorum: Stocker et al., 2021

Crocodylus acer: Mook, 1921; AMNH 7121

Crocodylus affinis: Mook, 1921; AMNH 1213, AMNH 1719, AMNH

6166, AMNH 16609, AMNH 16622, AMNH 6176, YPM 1352, YPM 265, YPM 266, YPM

246

Crocodylus megarhinus: Mook, 1927; Müller, 1927; Andrews, 2016; AMNH 5061, AMNH 5062

Crocodylus niloticus: O'Brien et al., 2019; Paiva et al., 2022

Crocodylus porosus: O'Brien et al., 2019; Paiva et al., 2022

Crocodylus rhombifer: O'Brien et al., 2019; Paiva et al., 2022

Deinosuchus riograndensis: Cossette & Brochu, 2020; AMNH 3073 (holotype)

Deinosuchus schwimmeri: Cossette & Brochu, 2020

Diplocynodon darwini: Ludwig, 1877; SMF-Me 896, SMF-Me 898, SMF-Me 900, SMF-Me 1137, SMF-Me 1289, SMF-Me 2748, SMF-Me 3780; HLMD-Me 233, HLMD-Me 236, HLMD-Me 5317, HLMD-Me 5349, HLMD-Me 5485, HLMD-Me 7492, HLMD-Me 7500, HLMD-Me 10262, HLMD-Me 10496, HLMD-Me 14600a; GMH-XXXVI 524; GMH 6077; SMNK-PAL 6517

Diplocynodon deponiae: Delfino & Smith, 2012; SMF-Me 899; SMF-Me 2609; HLMD-Me 147; HLMD-Me 8080; HLMD-Me 7496

Diplocynodon hantoniensis: Rio et al., 2020; Rio & Mannion, 2021; specimens in Rio et al. (2020)

Diplocynodon muelleri: Piras & Buscalioni, 2006; NMB-Spa 4 (holotype); NMB-Spa 73

Diplocynodon ratelii: MNHN-SG 599; MNHN 13728a; MNHN 13728b

Diplocynodon remensis: Martin et al., 2014b; MNHN-F-BR 4020 (holotype); MNHN-F-BR 13105; MNHN-F-BR 13106; MNHN-F-BR 13100; MNHN-F-BR 3501; CT-scan of CE 0001

Diplocynodon tormis: Buscalioni et al., 1992; IPS 36361 (holotype, IPS-9001 in Buscalioni et al., 1992; housed in ICP)

Dollosuchoides densmorei: Brochu, 2007

Dongnanosuchus hsui: Shan et al., 2021

Eoalligator chunyii: Wu et al., 2018; IVPP V.2716

Eocaiman cavernensis: Simpson, 1933; Godoy et al., 2021; AMNH 3158 (holotype)

Eocaiman itaboraiensis: Pinheiro, 2013

Eocaiman palaeocenicus: Bona, 2007

Eogavialis africanum: Andrews, 1901; Müller, 1927; YPM 6263;

Eosuchus lerichei: Delfino et al., 2005

Eosuchus minor: Brochu, 2006

Eothoracosaurus mississippiensis: Brochu, 2004a

Euthecodon arambourgi: Ginsburg, 1978

Gavialis gangeticus: O'Brien et al., 2019; Paiva et al., 2022;

Gavialis lewisi: Lull, 1944; YPM VP 3226

Gavialosuchus eggenbrugensis: Toula & Kail, 1885; Nicholl et al., 2020

Globidentosuchus brachyrostris: Scheyer, 2013; Scheyer & Delfino, 2016

Gnatusuchus pebasensis: Salas-Gismondi et al., 2015; Cidade et al., 2019; Rio & Mannion, 2021; photos from Rodolfo Salas-Gismondi

Gryposuchus colombianus: Salas-Gismondi et al., 2016

Hassiacosuchus haupti: HLMD-Me 4415; HLMD-Me-137; HLMD-Me 9119; HLMD-Me 6117; GMH-Ce-IV 6042

Hylaeochampsa vectiana: Clark, 1992; NHMUK R177

Iharkutosuchus makadii: Ösi, 2008; Ösi & Weishampel, 2009; material in MTM

Jiangxisuchus nankangensis: Li et al., 2019

Kambara implexidens: Salisbury, 1996

Kentisuchus spenceri: Brochu, 2007

Krabisuchus siamogallicus: Martin & Laupraset, 2010; Kr-C-006; Kr-C-007; Kr-C-010; Kr-C-012; Kr-C-015

Kuttanacaiman iquitosensis: Salas-Gismondi et al., 2015

La Venta Caiman: Langston, 1965

Leidyosuchus canadensis: Wu et al., 2001; Brochu, 1997; RTMP 86.221.01, RTMP 96.12.74

Lohuecosuchus mechinorum: Narvaez et al., 2015

Lohuecosuchus megadontos: Narvaez et al., 2015

Maomingosuchus petrolica: Shan et al., 2017

Mecistops cataphractus: O'Brien et al., 2019; Paiva et al., 2022

Melanosuchus niger: Vieira et al., 2016; O'Brien et al., 2019; Paiva et al., 2022

Mourasuchus amazonensis: Paiva et al., 2022

Mourasuchus arendsi: Paiva et al., 2022

Mourasuchus atopus: Paiva et al., 2022

Navajosuchus mooki: Lucas & Estep, 2000; Brochu, 2004b; AMNH 6780, AMNH 5186

Necrosuchus ionensis: Brochu, 2011; Cidade et al., 2019a

Orientalosuchus naduongensis: Massonne et al., 2019; GPIT/RE/09761; GPIT/RE/09730; GPIT/RE/09729; GPIT/RE/09784; GPIT/RE/09727

Osteolaemus osborni: O'Brien et al., 2019; Paiva et al., 2022

Osteolaemus tetraspis: O'Brien et al., 2019; Paiva et al., 2022

Pachycheilosuchus trinquei: Rogers, 2003

Paleosuchus palpebrosus: O'Brien et al., 2019; Paiva et al., 2022

Paleosuchus trigonatus: O'Brien et al., 2019; Paiva et al., 2022

Paratomistoma courti: Brochu, 2000

Pietraroiasuchus ormezzonai: Buscalioni et al., 2011

Piscogavialis jugaliperforatus: Kraus, 1998; Salas-Gismondi, 2016; SMNK 1282 PAL

Planocrania datangensis: Brochu, 2012; IVPP V5016

Planocrania hengdongensis: Brochu, 2012; IVPP V6074

Procaimanoidea kayi: Mook, 1941a; Wasserug & Hecht, 1967; Brochu, 1999

Procaimanoidea utahensis: Gilmore, 1946

Prodiplocynodon langi: Mook, 1941b; AMNH FARB 108

Protoalligator huiningensis: Wang et al., 2016; Massonne et al., 2019

Protocaiman peligrensis: Bona et al., 2018; Shan et al., 2021

Purussaurus brasiliensis: Aguilera, 2006; Paiva et al., 2022

Purussaurus mirandai: Aguilera, 2006; Scheyer et al., 2019; Paiva et al., 2022

Purussaurus neivensis: Paiva et al., 2022

Quinkana spp.*:* Molnar, 1981; Rossmann, 1998

Rimasuchus lloydi: Storrs, 2003

Shamosuchus djadochtaensis: Pol, 2009; Turner, 2015; AMNH FARB 6412

Stangerochampsa mccabei: Wu, 1996; digital collection of RTMP

Thecachampsa antiqua: Myrick, 2001; Weems, 2018

Thoracosaurus macrorhynchus: Koken, 1888; Voiculescu-Holvad, 2022; (Ca.)

Thoracosaurus neocesariensis: Voiculescu-Holvad, 2022

Tomistoma cairense: Müller, 1927

Tomistoma lusitanica:Antunes, 1961

Tomistoma schlegelii: O'Brien et al., 2019; Paiva et al., 2022

Toyotamaphimeia machikanensis: Kobayashi, 2006

Trilophosuchus rackhami: Willis, 1993; Ristevski et al., 2021

Tsoabichi greenriverensis: Brochu, 2010; Walter et al., 2022; SMNK-PAL 2333a; SMNK-PAL 2333b; SMNK-PAL 2334; AMNH FR 3666

Voay robustus: Brochu, 2007; Bickelmann & Klein, 2009; AMNH FR 3101; AMNH FR 3102; MNH FR 3103; AMNH FR 310; AMNH FR 3105

Wannaganosuchus brachymanus: Brochu, 1999; Erickson, 1982

Institutional abbreviatons:

ACAP: Association Culturelle, Archéologique et Paléontologique de l’Ouest Biterrois, Cruzy, France;
AMNH: American Museum of Natural History, New York, USA;
GMH: Geiseltal Museum of Martin-Luther-University Halle-Wittenberg, Halle (Saale), Germany;
GPIT: Geologisch-Paläontologisches Institut Tübingen, Tübingen, Germany;
HLMD: Hessisches Landesmuseum Darmstadt, Darmstadt, Germany;
IPS: Institut de Paleontologia Miquel Crusafont of Sabadell, Sabadell, Spain (= **ICP**; Institut Català de Paleontologia Miquel Crusafont);
IRScNB: Institut Royal des Sciences Naturelles de Belgique, Brussels, Belgium;
IVPP: Institute of Vertebrate Paleontology and Paleoanthropology, Beijing, China;
Kr-C: Sirindhorn Museum, Kalasin Province, Thailand;
MAFI: Magyar Állami Földtani Intézet, Hungarian Geological Institute, Budapest, Hungary;
MCSNT: Museo Civico di Storia naturale di Trieste, Trieste, Italy;
MNHM: Museum National d'Histoire Naturelle, Paris, France;
MTM: Magyar Természettudományi Múzeum, (Natural History Museum of Hungary), Budapest, Hungary;
NHMB: Naturhistoriches Museum Basel, Basel, Switzerland;
PSMUBB: Paleontology-Stratigraphy Museum, University Babes¸-Bolyai, Cluj-Napoca, Romania;
RTMP: Royal Tyrrell Museum of Palaeontology, Drumheller, Canada;
SMM: Science Museum of Minnesota, Saint Paul, Minnesota, USA;
SMNK: Staatliches Museum für Naturkunde Karlsruhe, Karlsruhe, Germany;
SMF: Senckenberg Museum Frankfurt, Frankfurt, Germany;
TMM: Texas Memorial Museum, Austin, Texas, USA;
USNM: United States National Museum, Smithsonian Institution, Washington DC, USA;
YPM: Yale Peabody Museum of Natural History, Yale University, New Haven, Connecticut, USA.

Table S1. List of extant and extinct gigantic crocodyliforms (≥ 7m in total length)

Age	Species	Source for body-size estimate and association with aquatic mega-ecosystems
Present	*Crocodylus porosus*	Britton et al., 2012
Pleistocene	Kali Gedeh *Crocodylus*	Delfino and De Vos, 2014; Sémah et al., 2010, 2016
Plio-Pleistocene	*Crocodylus thorbjarnarsoni*; *Euthecodon brumpti*	Brochu & Storrs, 2012; Brochu, 2020; Nutz et al., 2020
Mio-Pliocene	*Rhamphosuchus crassidens*	Head, 2001; More et al., 2016; Behrensmeyer et al., 2005; Martin, 2019
Mio-Pliocene	*Gryposuchus croizati*	Riff and Aguilera, 2008; Kern et al., 2020
Miocene	*Purussaurus brasiliensis*	Aureliano et al., 2015; Kern et al., 2020
Miocene	*Piscogavialis jugaliperforatus*	Kraus, 1998; Di Celma et al., 2016; Collareta et al., 2021
late Miocene	*Mourasuchus mirandai*	Aguilera et al. 2006; Kern et al., 2020
late Oligocene	*Astorgosuchus bugtiensis*	Martin et al., 2019
early Eocene	*Phosphatosaurus gavialoides*	Buffetaut, 1979
Campanian	*Deinosuchus riograndensis*	Erickson & Brochu, 1999; Schwimmer, 2002
Cenomanian	*Stomatosuchus inermis*	Stromer, 1925; Smith et al., 2001; Khalifa et al. 2008
Aptian–Albian	*Sarcosuchus imperator*	Sereno et al., 2001; Dridi, 2018
Early Cretaceous	*Chalawan thailandicus*	Buffetaut & Ingavat, 1980; Martin et al., 2014a

4. LIST OF MORPHOLOGICAL CHARACTERS

This list includes the characters in our analysis of the modified Massonne et al. (2019) matrix. The following eleven multistate characters form morphoclines and were therefore ordered in the phylogenetic analysis: 39, 48, 62, 81, 124, 149, 151, 154, 159, 193 and 203.

Character states were added to the following characters:

- 46, 48, 70, 79, 128, 137, and 156 (from Salas-Gismondi et al., 2015)
- 53, 80, 136, and 155 (from Cossette & Brochu, 2020)
- 151 and 159 (from Walter et al., 2022)

The added states are highlighted in bold below and the characters were rescored.
- 103 was adapted from the dataset of Rio & Mannion (2021)

(1) Fused proatlas boomerang-shaped (0), strap-shaped (1), or massive and block-shaped (2).

(2) Proatlas with prominent anterior process (0) or lacks anterior process (1).

(3) Proatlas has tall dorsal keel (0) or lacks tall dorsal keel; dorsal side smooth (1).

(4) Atlas intercentrum wedge-shaped in lateral view, with insignificant parapophyseal processes (0), or plate-shaped in lateral view, with prominent parapophyseal processes at maturity (1).

(5) Dorsal margin of atlantal rib generally smooth with modest dorsal process (0) or with prominent process (1).

(6) Atlantal ribs without (0) or with (1) very thin medial laminae at anterior end.

(7) Atlantal ribs lack (0) or possess (1) large articular facets at anterior ends for each other.

(8) Axial rib tuberculum wide, with broad dorsal tip (0) or narrow, with acute dorsal tip (1).

(9) Axial rib tuberculum contacts diapophysis late in ontogeny, if at all (0) or early in ontogeny (1).

(10) Anterior half of axis neural spine oriented horizontally (0) or slopes anteriorly (1).

(11) Axis neural spine crested (0) or not crested (1).

(12) Posterior half of axis neural spine wide (0) or narrow (1).

(13) Axis neural arch lacks (0) or possesses (1) a lateral process (diapophysis).

(14) Axial hypapophysis located toward the center of centrum (0) or toward the anterior end of centrum (1).

(15) Axial hypapophysis without (0) or with (1) deep fork.

(16) Hypapophyseal keels present on eleventh vertebra behind atlas (0), twelfth vertebra behind atlas (1), or tenth vertebra behind atlas (2).

(17) Third cervical vertebra (first postaxial) with prominent hypapophysis (0) or lacks prominent hypapophysis (1).

(18) Neural spine on third cervical long, dorsal tip at least half the length of the centrum without the cotyle (0) or short, dorsal tip acute and less than half the length of the centrum without the cotyle (1).

(19) Cervical and anterior dorsal centra lack (0) or bear (1) deep pits on the ventral surface of the centrum.

(20) Presacral centra amphicoelous (0) or procoelous (1).

(21) Anterior sacral rib capitulum projects far anteriorly of tuberculum and is broadly visible in dorsal view (0), or anterior margins of tuberculum and capitulum nearly in same plane, and capitulum largely obscured dorsally (1).

(22) Scapular blade flares dorsally at maturity (0) or sides of scapular blade subparallel; minimal dorsal flare at maturity (1).

(23) Deltoid crest of scapula very thin at maturity, with sharp margin (0) or very wide at maturity, with broad margin (1).

(24) Scapulocoracoid synchondrosis closes very late in ontogeny (0) or relatively early in ontogeny (1).

(25) Scapulocoracoid facet anterior to glenoid fossa uniformly narrow (0) or broad immediately anterior to glenoid fossa, and tapering anteriorly (1).

(26) Proximal edge of deltopectoral crest emerges smoothly from proximal end of humerus and is not obviously concave (0) or emerges abruptly from proximal end of humerus and is obviously concave (1).

(27) M. teres major and M. dorsalis scapulae insert separately on humerus; scars can be distinguished dorsal to deltopectoral crest (0) or insert with common tendon; single insertion scar (1).

(28) Olecranon process of ulna narrow and sub-angular (0) or wide and rounded (1).

(29) Distal extremity of ulna expanded transversely with respect to long axis of bone; maximum width equivalent to that of proximal extremity (0) or proximal extremity considerably wider than distal extremity (1).

(30) Interclavicle flat along length, without dorsoventral flexure (0) or with moderate dorsoventral flexure (1) or with severe dorsoventral flexure (2).

(31) Anterior end of interclavicle flat (0) or rod-like (1).

(32) Iliac anterior process prominent (0) or virtually absent (1).

(33) Dorsal margin of iliac blade rounded with smooth border (0) or rounded, with modest dorsal indentation (1) or rounded, with strong dorsal indentation (wasp-waisted; 2) or narrow, with dorsal indentation (3) or rounded with smooth border; posterior tip of blade very deep (4).

(34) Supraacetabular crest narrow (0) or broad (1).

(35) Limb bones relatively robust, and hind limb much longer than forelimb at maturity (0) or limb bones very long and slender (1).

(36) M. caudofemoralis with single head (0) or with double head (1).

(37) Dorsal osteoderms not keeled (0) or keeled (1).

(38) Dorsal midline osteoderms rectangular (0) or nearly square (1).

(39) Four (0), six (1), eight (2), or ten (3) contiguous dorsal osteoderms per row at maturity. (ORDERED)

(40) Nuchal shield grades continuously into dorsal shield (0) or differentiated from dorsal shield; four nuchal osteoderms (1) or differentiated from dorsal shield; six nuchal osteoderms with four central and two lateral (2) or differentiated from dorsal shield; eight nuchal osteoderms in two parallel rows (3).

(41) Ventral armor absent (0) or single ventral osteoderms (1) or paired ventral ossifications that suture together (2).

(42) Anterior margin of dorsal midline osteoderms with anterior process (0) or smooth, without process (1).

(43) Ventral scales have (0) or lack (1) follicle gland pores.

(44) Ventral collar scales not enlarged relative to other ventral scales (0) or in a single enlarged row (1) or in two parallel enlarged rows (2).

(45) Median pelvic keel scales form two parallel rows along most of tail length (0) or form single row along tail (1) or merge with lateral keel scales (2).

(46) Alveoli for dentary teeth 3 and 4 nearly same size and confluent (0), fourth alveolus larger than third, and alveoli are separated (1), **or 3 and 4 are nearly the same size and separated (2)**. [modified by Salas-Gismondi et al. (2015)].

(47) Anterior dentary teeth strongly procumbent (0) or project anterodorsally (1).

(48) Dentary symphysis extends to fourth or fifth alveolus (0), or sixth through eighth alveolus (1), **or eight to twelfth alveolus (2), or twelfth to sixteenth (3), beyond the sixteenth (4)**. [modified by Salas-Gismondi et al. (2015)]. (ORDERED)

(49) Dentary gently curved (0), deeply curved (1), or linear (2) between fourth and tenth alveoli.

(50) Largest dentary alveolus immediately caudal to fourth is (0) 13 or 14, (1) between 11 and 14 and a series behind it, (2) 11 or 12, (3) no differentiation, (4) behind 14, (5) 10.

(51) Splenial with anterior perforation for mandibular ramus of cranial nerve V (0) or lacks anterior perforation for mandibular ramus of cranial nerve V (1).

(52) Mandibular ramus of cranial nerve V exits splenial anteriorly only (0) or splenial has singular perforation for mandibular ramus of cranial nerve V posteriorly (1) or splenial has double perforation for mandibular ramus of cranial nerve V posteriorly (2).

(53) Splenial participates in mandibular symphysis; splenial symphysis adjacent to no more than five dentary alveoli (0) or splenial excluded from mandibular symphysis; anterior tip of splenial passes ventral to Meckelian groove (1) or splenial excluded from mandibular symphysis anterior tip of splenial passes dorsal to Meckelian groove (2) or deep splenial symphysis, longer than five dentary alveoli; splenial forms wide ‘V’ within symphysis (3) or deep splenial symphysis, longer than five dentary alveoli; splenial constricted within symphysis and forms narrow ‘V’ (4) **or splenial reaches mandibular symphysis but does not touch other splenial** (5). [modified by Cossette & Brochu (2020)].

(54) Coronoid bounds posterior half of foramen intermandibularis medius (0) or completely surrounds foramen intermandibularis medius at maturity (1) or obliterates foramen intermandibularis medius at maturity (2).

(55) Superior edge of coronoid slopes strongly anteriorly (0) or almost horizontal (1).

(56) Inferior process of coronoid laps strongly over inner surface of Meckelian fossa (0) or remains largely on medial surface of mandible (1).

(57) Coronoid imperforate (0) or with perforation posterior to foramen intermandibularis medius (1).

(58) Process of splenial separates angular and coronoid (0) or no splenial process between angular and coronoid (1).

(59) Angular-surangular suture contacts external mandibular fenestra at posterior angle at maturity (0) or passes broadly along ventral margin of external mandibular fenestra late in ontogeny (1).

(60) Anterior processes of surangular unequal (0) or sub-equal to equal (1).

(61) Surangular with spur bordering the dentary toothrow lingually for at least one alveolus length (0) or lacking such spur (1).

(62) External mandibular fenestra absent (0) or present as narrow slit, no discrete fenestral concavity on angular dorsal margin (1) or present with discrete concavity on

angular dorsal margin (2) or present and very large; most of foramen intermandibularis caudalis visible in lateral view (3). (ORDERED)

(63) Surangular-dentary suture intersects external mandibular fenestra anterior to posterodorsal corner (0) or at posterodorsal corner (1).

(64) Angular extends dorsally toward or beyond anterior end of foramen intermandibularis caudalis; anterior tip acute (0) or does not extend dorsally beyond anterior end of foramen intermandibularis caudalis; anterior tip very blunt (1).

(65) Surangular-angular suture lingually meets articular at ventral tip (0) or dorsal to tip (1).

(66) Surangular continues to dorsal tip of lateral wall of glenoid fossa (0) or truncated and not continuing dorsally (1).

(67) Articular-surangular suture simple (0) or articular bears anterior lamina dorsal to lingual foramen (1) or articular bears anterior lamina ventral to lingual foramen (2) or bears laminae above and below foramen (3).

(68) Lingual foramen for articular artery and alveolar nerve perforates surangular entirely (0) or perforates surangular/angular suture (1).

(69) Foramen aerum at extreme lingual margin of retroarticular process (0) or set in from margin of retroarticular process (1).

(70) Retroarticular process projects posteriorly (0) projects posterodorsally, **not higher than the posterior edge of the articular fossa (1), or projects posterodorsally higher than the posterior edge of the articular fossa (2)**. [modified by Salas-Gismondi et al. (2015)]

(71) Surangular extends to posterior end of retroarticular process (0) or pinched off anterior to tip of retroarticular process (1).

(72) Surangular-articular suture oriented anteroposteriorly (0) or bowed strongly laterally (1) within glenoid fossa.

(73) Sulcus between articular and surangular (0) or articular flush against surangular (1).

(74) Dorsal projection of hyoid cornu flat (0) or rod-like (1).

(75) Dorsal projection of hyoid cornu narrow, with parallel sides (0) or flared (1).

(76) Lingual osmoregulatory pores small (0) or large (1).

(77) Tongue with (0) or without (1) keratinized surface.

(78) Teeth and alveoli of maxilla and/or dentary circular in cross-section (0), or posterior teeth laterally compressed (1), or all teeth compressed (2).

(79) Maxillary and dentary teeth with smooth carinae (0) serrated (1), **or with neither carinae nor serrations (2)**. [modified by Salas-Gismondi et al. (2015)]

(80) Naris projects anterodorsally (0) or dorsally (1) **or posterodorsally (2)**. [modified by Cossette & Brochu (2020)]

(81) External naris bisected by nasals (0) or nasals contact external naris, but do not bisect it (1) or nasals excluded, at least externally, from naris; nasals and premaxillae still in contact (2) or nasals and premaxillae not in contact (3). (ORDERED)

(82) Naris circular or keyhole-shaped (0) or wider than long (1) or anteroposteriorly long and prominently teardrop-shaped (2).

(83) External naris of reproductively mature males (0) remains similar to that of females or (1) develops bony excrescence (ghara).

(84) External naris (0) opens flush with dorsal surface of premaxillae or (1) circumscribed by thin crest.

(85) Premaxillary surface lateral to naris smooth (0) or with deep notch lateral to naris (1).

(86) Premaxilla has five teeth (0) or four teeth (1) early in posthatching ontogeny.

(87) Incisive foramen small, less than half the greatest width of premaxillae (0) or large, more than half the greatest width of premaxillae (1) or large, and intersects premaxillary-maxillary suture (2).

(88) Incisive foramen completely situated far from premaxillary tooth row, at the level of the second or third alveolus (0) or abuts premaxillary tooth row (1) or projects between first premaxillary teeth (2).

(89) Dorsal premaxillary processes short, not extending beyond third maxillary alveolus (0) or long, extending beyond third maxillary alveolus (1).

(90) Dentary tooth 4 occludes in notch between premaxilla and maxilla early in ontogeny (0) or occludes in a pit between premaxilla and maxilla; no notch early in ontogeny (1).

(91) All dentary teeth occlude lingual to maxillary teeth (0) or occlusion pit between seventh and eighth maxillary teeth; all other dentary teeth occlude lingually (1) or dentary teeth occlude in line with maxillary toothrow (2).

(92) Largest maxillary alveolus is 3 (0), 5 (1), 4 (2), 4 and 5 are same size (3), 6 (4), or maxillary teeth homodont (5), or maxillary alveoli gradually increase in diameter posteriorly toward penultimate alveolus (6).

(93) Maxillary tooth row curved medially or linear (0) or curves laterally broadly (1) posterior to first six maxillary alveoli.

(94) Dorsal surface of rostrum curves smoothly (0) or bears medial dorsal boss (1).

(95) Canthi rostralii absent or very modest (0) or very prominent (1) at maturity.

(96) Preorbital ridges absent or very modest (0) or very prominent (1) at maturity.

(97) Antorbital fenestra present (0) or absent (1).

(98) Vomer entirely obscured by premaxilla and maxilla (0) or exposed on palate at premaxillary-maxillary suture (1).

(99) Vomer entirely obscured by maxillae and palatines (0) or exposed on palate between palatines (1).

(100) Surface of maxilla within narial canal imperforate (0) or with a linear array of pits (1).

(101) Medial jugal foramen small (0) or very large (1).

(102) Maxillary foramen for palatine ramus of cranial nerve V small or not present (0) or very large (1).

(103) Ectopterygoid, contact with maxillary toothrow, forming the medial wall of at least one maxillary alveolus: absent, ectopterygoid-maxilla suture anteromedially orientated and separated from toothrow margin (0); absent, ectopterygoid-maxilla suture parallel and adjacent to medial toothrow margin (1); present (2) (modified by Rio & Mannion, 2021).

(104) Maxilla terminates in palatal view anterior to lower temporal bar (0) or comprises part of the lower temporal bar (1).

(105) Penultimate maxillary alveolus less than (0) or more than (1) twice the diameter of the last maxillary alveolus.

(106) Prefrontal dorsal surface smooth adjacent to orbital rim (0) or bearing discrete knob-like processes (1).

(107) Dorsal half of prefrontal pillar narrow (0) or expanded anteroposteriorly (1).

(108) Medial process of prefrontal pillar expanded dorsoventrally (0) or anteroposteriorly (1).

(109) Prefrontal pillar solid (0) or with large pneumatic recess (1).

(110) Medial process of prefrontal pillar wide (0) or constricted (1) at base.

(111) Maxilla has linear medial margin adjacent to suborbital fenestra (0) or bears broad shelf extending into fenestra, making lateral margin concave (1).

(112) Anterior face of palatine process rounded or pointed anteriorly (0) or notched anteriorly (1).

(113) Anterior ectopterygoid process tapers to a point (0) or forked (1).

(114) Palatine process extends (0) or does not extend (1) significantly beyond anterior end of suborbital fenestra.

(115) Palatine process generally broad anteriorly (0) or in form of thin wedge (1).

(116) Lateral edges of palatines smooth anteriorly (0) or with lateral process projecting from palatines into suborbital fenestrae (1).

(117) Palatine-pterygoid suture nearly at (0) or far from (1) posterior angle of suborbital fenestra.

(118) Pterygoid ramus of ectopterygoid straight, posterolateral margin of suborbital fenestra linear (0) or ramus bowed, posterolateral margin of fenestra concave (1).

(119) Lateral edges of palatines parallel posteriorly (0) or flare posteriorly, producing shelf (1).

(120) Anterior border of the choana is comprised of the palatines (0) or choana entirely surrounded by pterygoids (1).

(121) Choana projects posteroventrally (0) or anteroventrally (1) at maturity.

(122) Pterygoid surface lateral and anterior to internal choana flush with choanal margin (0) or pushed inward anterolateral to choanal aperture (1) or pushed inward around choana to form neck surrounding aperture (2) or everted from flat surface to form neck surrounding aperture (3).

(123) Posterior rim of internal choana not deeply notched (0) or deeply notched (1).

(124) Internal choana not septate (0) or with septum that remains recessed within choana (1) or with septum that projects out of choana (2). (ORDERED)

(125) Ectopterygoid-pterygoid flexure disappears during ontogeny (0) or remains throughout ontogeny (1).

(126) Ectopterygoid extends (0) or does not extend (1) to posterior tip of lateral pterygoid flange at maturity.

(127) Lacrimal makes broad contact with nasal; no posterior process of maxilla (0) or maxilla with posterior process within lacrimal (1) or maxilla with posterior process between lacrimal and prefrontal (2).

(128) Prefrontals separated by **the** frontals and nasals, **anterior process of frontal extending far anterior to the anterior margin of the orbit (0), prefrontals separated by the frontal and nasals, anterior process of frontal around the same level or posterior to the anterior margin of the orbit (1)** or prefrontals meet medially, **anterior process of frontal around the same level or posterior to the anterior margin of the orbit** (2). [modified by Salas-Gismondi et al. (2015)]

(129) Lacrimal longer than prefrontal (0), or prefrontal longer than lacrimal (1), or lacrimal and prefrontal both elongate and nearly the same length (2).

(130) Anterior tip of frontal (0) forms simple acute point or (1) forms broad, complex sutural contact with the nasals.

(131) Ectopterygoid extends along medial face of postorbital bar (0) or stops abruptly ventral to postorbital bar (1).

(132) Postorbital bar massive (0) or slender (1).

(133) Postorbital bar bears process that is prominent, dorsoventrally broad, and divisible into two spines (0) or bears process that is short and generally not prominent (1).

(134) Ventral margin of postorbital bar flush with lateral jugal surface (0) or inset from lateral jugal surface (1).

(135) Postorbital bar continuous with anterolateral edge of skull table (0) or inset (1).

(136) Margin of orbit flush with skull surface (0) or dorsal edges of orbits upturned (1) or orbital margin telescoped (2) **or anterior margins of orbit telescoped (3)**. [modified by Cossette & Brochu (2020)]

(137) **Anterior margin of orbit not upturned**, ventral margin gently circular (0) **or anterior margin upturned, ventral margin gently circular (1)**, **or anterior margin upturned**, ventral margin with a prominent notch (1). [modified by Salas-Gismondi et al. (2015)]

(138) Palpebral forms from single ossification (0) or from multiple ossifications (1).

(139) Quadratojugal spine prominent at maturity (0) or greatly reduced or absent at maturity (1).

(140) Quadratojugal spine low, near posterior angle of infratemporal fenestra (0) or high, between posterior and superior angles of infratemporal fenestra (1).

(141) Quadratojugal forms posterior angle of infratemporal fenestra (0) or jugal forms posterior angle of infratemporal fenestra (1) or quadratojugal-jugal suture lies at posterior angle of infratemporal fenestra (2).

(142) Postorbital neither contacts quadrate nor quadratojugal medially (0) or contacts quadratojugal, but not quadrate, medially (1) or contacts quadrate and quadratojugal at dorsal angle of infratemporal fenestra (2) or contacts quadratojugal with significant descending process (3).

(143) Quadratojugal bears long anterior process along lower temporal bar (0) or bears modest process, or none at all, along lower temporal bar (1).

(144) Quadratojugal extends to superior angle of infratemporal fenestra (0) or does not extend to superior angle of infratemporal fenestra; quadrate participates in fenestra (1).

(145) Postorbital-squamosal suture oriented ventrally (0) or passes medially (1) ventral to skull table.

(146) Dorsal and ventral rims of squamosal groove for external ear valve musculature parallel (0) or squamosal groove flares anteriorly (1).

(147) Quadrate and squamosal not in contact on the external surface of the skull, posteriorly to the external auditory meatus (0) or quadratosquamosal suture extends dorsally along caudal margin of the external auditory meatus (1) or extends only to the caudoventral corner of the external auditory meatus (2).

(148) Caudal margin of otic aperture not defined and gradually merging into the exoccipital (0) or smooth and continuous with the paraoccipital process (1) or caudal margin of otic aperture inset (2).

(149) Frontoparietal suture deeply within supratemporal fenestra; frontal prevents broad contact between postorbital and parietal (0) or suture makes modest entry into supratemporal fenestra at maturity; postorbital and parietal in broad contact (1) or suture on skull table entirely (2). (ORDERED)

(150) Frontoparietal suture concavoconvex (0) or linear (1) between supratemporal fenestrae.

(151) Supratemporal fenestra with fossa; dermal bones of skull roof do not overhang rim at maturity (0), **or parietal overhangs the rim at maturity (1), or postorbital, squamosal and parietal overhang the rim at maturity (2)**, or fenestra is closed at maturity (3). [modified by Walter et al. (2022)] (ORDERED)

(152) Shallow fossa at anteromedial corner of supratemporal fenestra (0) or no such fossa; anteromedial corner of supratemporal fenestra smooth (1).

(153) Medial parietal wall of supratemporal fenestra imperforate (0) or bearing foramina (1).

(154) Parietal and squamosal widely separated by quadrate on posterior wall of supratemporal fenestra (0) or parietal and squamosal approach each other on posterior wall of supratemporal fenestra without actually making contact (1) or parietal and squamosal meet along posterior wall of supratemporal fenestra (2). (ORDERED)

(155) Skull table surface slopes ventrally from sagittal axis (0) or planar (1) **or skull table surface slopes ventrally towards sagittal axis at maturity, lateral elements planar (2)** at maturity. [modified by Cossette & Brochu (2020)]

(156) Squamosal **on skull table** is horizontal or nearly so (0), or upturned to form a **posterolateral** discrete horn (1), **or producing a high transversely oriented eminence at the posterior margin (2) late in ontogeny**. [modified by Salas-Gismondi et al. (2015)]

(157) Mature skull table with broad curvature; short posterolateral squamosal rami along paroccipital process (0) or with nearly horizontal sides; significant posterolateral squamosal rami along paroccipital process (1).

(158) Squamosal does not extend (0) or extends (1) ventrolaterally to lateral extent of paraoccipital process.

(159) Supraoccipital exposure on dorsal skull table **absent (0), small (1)**, large (2), or large such that parietal is excluded from posterior edge of table (3). [modified by Walter et al. (2021)] (ORDERED)

(160) Anterior foramen for palatine ramus of cranial nerve VII ventrolateral (0) or ventral (1) to basisphenoid rostrum.

(161) Sulcus on anterior braincase wall lateral to basisphenoid rostrum (0) or braincase wall lateral to basisphenoid rostrum smooth; no sulcus (1).

(162) Basisphenoid not exposed extensively (0) or exposed extensively (1) on braincase wall anterior to trigeminal foramen.

(163) Extensive exposure of prootic on external braincase wall (0) or prootic largely obscured by quadrate and laterosphenoid externally (1).

(164) Laterosphenoid bridge comprised entirely of laterosphenoid (0) or with ascending process or palatine (1).

(165) Capitate process of laterosphenoid oriented laterally (0) or anteroposteriorly (1) toward midline.

(166) Parietal with recess communicating with pneumatic system (0) or solid, without recess (1).

(167) Significant ventral quadrate process on lateral braincase wall (0) or quadrate-pterygoid suture linear from basisphenoid exposure to trigeminal foramen (1).

(168) Lateral carotid foramen opens lateral (0) or dorsal (1) to basisphenoid at maturity.

(169) External surface of basioccipital ventral to occipital condyle oriented posteroventrally (0) or posteriorly (1) at maturity.
(170) Posterior pterygoid processes tall and prominent (0) or small and project posteroventrally (1) or small and project posteriorly (2).

(171) Basisphenoid thin (0) or anteroposteriorly wide (1) ventral to basioccipital.

(172) Basisphenoid not broadly exposed ventral to basioccipital at maturity; pterygoid short ventral to median eustachian opening (0) or basisphenoid exposed as broad sheet ventral to basioccipital at maturity; pterygoid tall ventral to median eustachian opening (1).

(173) Exoccipital with very prominent boss on paroccipital process; process lateral to cranioquadrate opening short (0) or exoccipital with small or no boss on paroccipital process; process lateral to cranioquadrate opening long (1).

(174) Lateral eustachian canals open dorsal (0) or lateral (1) to medial eustachian canal.

(175) Exoccipitals terminate dorsal to basioccipital tubera (0) or send robust process ventrally and participate in basioccipital tubera (1) or send slender process ventrally to basioccipital tubera (2).

(176) Quadrate foramen aerum on mediodorsal angle (0) or on dorsal surface (1) of quadrate.

(177) Quadrate foramen aereum is small (0), comparatively large (1), or absent (2) at maturity.

(178) Quadrate lacks (0) or bears (1) prominent, mediolaterally thin crest on dorsal surface of ramus.

(179) Attachment scar for posterior mandibular adductor muscle on ventral surface of quadrate ramus forms modest crests (0) or prominent knob (1).

(180) Quadrate with small, ventrally-reflected medial hemicondyle (0) or with small medial hemicondyle; dorsal notch for foramen aerum (1) or with prominent dorsal projection between hemicondyles (2) or with expanded medial hemicondyle (3).

(181) Iris (0) greenish/yellowish or (1) brown.

(182) Two or more (0) or one (1) row of postoccipital osteoderms.

(183) Fewer than eight (0) or eight to 14 (1) or more than 14 (2) paired midline scale rows.

(184) Ectopterygoid maxillary ramus forms less than (0) or more than (1) two-thirds of lateral margin of suborbital fenestra.

(185) Ectopterygoid maxillary ramus terminates at lateral margin of suborbital fenestra (0) or lateral to it, with maxilla separating the ectopterygoid from fenestra for short distance.

(186) Palatine-maxillary suture intersects suborbital fenestra at its anteromedial margin (0) or nearly at its anteriormost limit (1).

(187) Frontal lacks (0) or bears (1) prominent midsagittal crest between orbits.

(188) All cervical neural spines anteroposteriorly broad (0) or posterior neural spines thin and rod-like (1).

(189) Largest premaxillary tooth is the second (0), the third (1) or the fourth (2) or the third and fourth similarly largest (3) or all similarly same in size (4) or the fourth and fifth similarly largest (5) or the first four similarly equal in size (6)

(190) Dorsal surface of the surangular is smooth (0), or bears a large sulcus next to the anterior half of the glenoid fossa (1).

(191) ‘U’-shaped depression of the frontal at the point of maximum constriction between the orbits: absent (0); present (1).

(192) Skull in lateral view relatively flat (0) or formed like a wedge **and trapezoid in shape** (1).

(193) Anterior process of jugal extends anterior (0), lies at the same level as (1), or well posterior to the anterior process of frontal (2). (ORDERED)

(194) Notch between the premaxilla and maxilla present (0) or absent (1) in adult individuals.

(195) Anterior maxillary teeth without (0) or with (1) ridges on their lateral surface

(196) If largest dentary alveolus is between 11th and 14th and a series behind it, is it the (0) 11th, (1) 12th, or (2) 13th or 14th.

(197) Surangular-angular suture lingually originates (0) near the ventral border of the external mandibular fenestra, (1) near the dorsal border of the external mandibular fenestra and straight, (2) near the dorsal border of the external mandibukar fenestra and bowed.

(198) If supraoccipital exposure on skull table is large or very large, is it (0) trapezoid, (1) triangular, or (2) block-shaped.

(199) Edge of the maxillary tooth alveoli lower or at the same level than the space between toothrow (0) or edge of maxillary tooth alveoli higher than the space (1). [added from Stocker et al. (2021)]

(200) Ventral border of exoccipital convex and ventrally projected, hiding the posterior opening of the cranioquadrate passage from the occipital view (0), or straight, sharpen or smoothly convex and does not hide the posterior opening of the cranioquadrate passage from the occipital view (1). [added from Stocker et al. (2021)]

(201) Occipital surface sloped, visible in dorsal view (0), or vertical or not visible in dorsal view (1) at maturity. [added from Stocker et al. (2021)]

(202) Ventral premaxilla-maxilla suture short and ends posteriorly before the 3rd maxillary alveoli (0) or elongated and extends or exceeds the 3rd maxillary alveoli (1). [added from Stocker et al. (2021)]

(203) Less than 18 teeth (0), 18 to 22 teeth (1), or more than 22 teeth (2) on maxilla. [added from Stocker et al. (2021)] (ORDERED)

(204) Lateral edge of the skull table at the level of the postorbital-squamosal suture situated laterally or at the same level as (0), or medially to (1) the quadrate condyle in dorsal view at maturity. [added from Stocker et al. (2021)]

(205) Frontal ends at the same level or posterior (0) or extends well anterior (1) to the anterior extension of the prefrontal. [added from Stocker et al. (2021)]

(206) Maxilla posterior process without tooth, short or absent (0), or long, longer than the distance between the three last teeth (1) in ventral view. [added from Stocker et al. (2021)]

(207) Interorbital bridge narrower to equivalent (0), or broader (1) than the width of the orbit. [added from Stocker et al. (2021)]

(208) Supratemporal fenestra longer than wide or rounded, posterior bar of supratemporal fenestra thick (0), or wider than long, posterior bar of supratemporal

fenestra thick (1), or wider than long, posterior bar thin (2) at maturity. [added from Stocker et al. (2021)]

(209) Presence (0), or absence (1) of a medial crest on the basioccipital. [added from Stocker et al. (2021)]

(210) Absence (0), or presence (1) of a posterior dentary process between splenial and angular on the ventral side. [added from Stocker et al. (2021)]

(211) Dorsal margin of the articular on the retroarticular process largely visible in lateral view (0), or slightly or not visible in lateral view (1). [added from Stocker et al. (2021)]

(212) Posterior margin of the orbit anterior to the posterior margin of the suborbital fenestra (0), or posterior or at the same level than the posterior margin of the suborbital fenestra (1) measured at the level of the postorbital-frontal suture in the orbital margin. [added from Stocker et al. (2021)]

(213) Basioccipital-exoccipital process ventral to occipital condyle (basioccipital plate) with parallel or ventrally convergent sides (0) or ventrally divergent sides (1) in posterior view. [added from Stocker et al. (2021)]

(214) Absence (0) or presence (1) of a smooth medial depression ventral to the basioccipital and posterior to the medial Eustachian foramen. [added from Stocker et al. (2021)]

(215) Dentary teeth series behind to alveoli 12-13 are pointed to slightly blunt (0); globular, different in size among them (1); globular, at least four subequal in size (2), molariform multicusped (3) or absent (4). [added from Stocker et al. (2021)]

(216) First four alveoli in the dentary are the same size or smaller than other dentary alveoli (0) or are the largest within the dentary (1). [added from Stocker et al. (2021)]

(217) Orbits longer than wide (0) or wider than long to rounded (1) late in ontogeny. [added from Stocker et al. (2021)]

(218) The series composed by the last three premaxillary teeth diverge posteriorly to paraxial (0) or tend to converge posteriorly, straight among them (1). [added from Stocker et al. (2021)]

(219) Dentary, level of the first and fourth alveoli: lower than the level of the eleventh and twelfth alveoli (0); equal to higher than the level of the eleventh and twelfth alveoli (1). [added from Stocker et al. (2021)].

5. REFERENCES

Adams, A.J. (2016). A reassessment of the late Eocene-early Oligocene crocodylids *Crocodylus megarhinus* Andrews 1905 and *Crocodylus articeps* Andrews 1905 from the Fayúm Province, Egypt. (Master of Science dissertation, The University of Iowa).

Andrews C.W. (1901). Preliminary note on some recently discovered extinct vertebrates from Egypt. *Geological Magazine*, 4: 436–444.

Aguilera, O.A., Riff, D., & Bocquentin-Villanueva, J. 2006. A new giant *Purussaurus* (Crocodyliformes, Alligatoridae) from the upper Miocene Urumaco formation, Venezuela. *Journal of Systematic Palaeontology*, 4(3), 221–232.

Antunes, M.T. (1961). *Tomistoma lusitanica*, crocodilien du Miocène du Portugal. Revista da Faculdade de Ciencias de Lisboa.

Aureliano T., Ghilardi A.M., Guilherme E., Souza-Filho J.P., Cavalcanti M., & Riff D. (2015). Morphometry, bite-force, and paleobiology of the Late Miocene Caiman *Purussaurus brasiliensis*. *PloS one*, 10:e0117944.

Bartels W.S. (1984). Osteology and systematic affinities of the horned alligator *Ceratosuchus* (Reptilia, Crocodilia). *Journal of Paleontology*, 58, 1347–1353.

Berg D.E. (1966). Die Krokodile, insbesondere ”*Asiatosuchus*” und aff. ”*Sebecus*?”, aus dem Eozaen von Messel bei Darmstadt/Hessen (Vol. 52). *Hessisches Landesamt für Bodenforschung*.

Behrensmeyer A.K., Badgley C., Barry J.C., Morgan M., & Raza S.M. (2005). The paleoenvironmental context of Siwalik Miocene vertebrate localities. In *Interpreting the Past* (pp. 47-62). Brill.

Bickelmann C. & Klein N. (2009). The late Pleistocene horned crocodile *Voay robustus* (Grandidier & Vaillant, 1872) from Madagascar in the museum für Naturkunde Berlin. *Fossil Record*, 12(1), 13–21.

Bona P. (2007). Una nueva especie de *Eocaiman* Simpson (Crocodylia, Alligatoridae) del Paleoceno Inferior de Patagonia. *Ameghiniana*, 44(2), 435–445.

Bona P., Blanco M.V.F., Scheyer T.M., & Both C. (2017). Shedding light on the taxonomic diversity of the South American Miocene caimans: the status of *Melanosuchus fisheri* (Crocodylia, Alligatoroidea). *Ameghiniana*, *54*(6), 681-687.

Bona P., Ezcurra M.D., Barrios F., & Fernandez Blanco, M.V. (2018). A new Palaeocene crocodylian from southern Argentina sheds light on the early history of caimanines. *Proceedings of the Royal Society B: Biological Sciences*, 285(1885), 20180843.

Brochu C.A. (1997). A review of ”*Leidyosuchus*” (Crocodyliformes, Eusuchia) from the Cretaceous through Eocene of North America. *Journal of Vertebrate Paleontology*, 17(4), 679–697.

Brochu C.A. (1999). Phylogenetics, taxonomy, and historical biogeography of Alligatoroidea. *Journal of Vertebrate Paleontology*, 19(S2), 9-100.

Brochu C.A. (2004a). A new Late Cretaceous gavialoid crocodylian from eastern North America and the phylogenetic relationships of thoracosaurs. *Journal of Vertebrate Paleontology*, 24(3), 610–633.

Brochu C.A. (2004b). Alligatorine phylogeny and the status of *Allognathosuchus* Mook, 1921. *Journal of Vertebrate Paleontology*, 24(4), 857–873.

Brochu C.A. (2006). Osteology and phylogenetic significance of *Eosuchus minor* (Marsh, 1870) new combination, a longirostrine crocodylian from the late Paleocene of North America. *Journal of Paleontology*, 80(1), 162–186.

Brochu C.A. (2007). Systematics and taxonomy of Eocene tomistomine crocodylians from Britain and Northern Europe. *Palaeontology*, 50(4), 917–928.

Brochu C.A. (2010). A new alligatorid from the lower Eocene Green River Formation of Wyoming and the origin of caimans. *Journal of Vertebrate Paleontology*, 30(4), 1109–1126.

Brochu C.A. (2011). Phylogenetic relationships of *Necrosuchus ionensis* Simpson, 1937 and the early history of caimanines. *Zoological Journal of the Linnean Society*, 163, S228–S256.

Brochu C.A. (2012). Phylogenetic relationships of Palaeogene ziphodont eusuchians and the status of *Pristichampsus* Gervais, 1853. *Earth and Environmental Science Transactions of the Royal Society of Edinburgh*, 103(3-4), 521-550.

Brochu C.A. (2020). Pliocene crocodiles from Kanapoi, Turkana Basin, Kenya. *Journal of Human Evolution*, 140, 102410.

Brochu C.A., & Gingerich P.D. (2000). New tomistomine crocodylian from the middle Eocene (Bartonian) of Wadi Hitan, Fayum Province, Egypt. *Contributions from the Museum of Paleontology*, The University of Michigan, 30(10), 251–268.

Brochu C.A., & Storrs G.W. (2012). A giant crocodile from the Plio-Pleistocene of Kenya, the phylogenetic relationships of Neogene African crocodylines, and the antiquity of *Crocodylus* in Africa. *Journal of Vertebrate Paleontology*, 32(3), 587–602.

Buffetaut E. (1979). Présence du crocodilien *Phosphatosaurus* (Mesosuchia, Dyrosauridae) dans le paléocène du Niger et du Mali. *Paläontologische Zeitschrift*, 53:323–333.

Buffetaut E., & Ingavat R. (1980). A new crocodilian from the Jurassic of Thailand, *Sunosuchus thailandicus* n. sp.(Mesosuchia, Goniopholididae)), and the palaeogeographical history of South-East Asia in the Mesozoic. *Geobios*, 13(6), 879-889.

Buscalioni A.D., & Sanz J.L. (1990). *Montsecosuchus depereti* (Crocodylomorpha, Atoposauridae), new denomination for *Alligatorium depereti* Vidal, 1915 (Early Cretaceous, Spain): redescription and phylogenetic relationships. *Journal of Vertebrate Paleontology*, 10(2), 244-254.

Buscalioni A.D., Sanz J.L., & Casanovas M.L. (1992). A new species of the eusuchian crocodile *Diplocynodon* from the Eocene of Spain. *Neues Jahrbuch für Geologie und Paläontologie Abhandlungen*,187, 1–29.

Buscalioni A.D., Piras P., Vullo R., Signore M., & Barbera C. (2011). Early eusuchia crocodylomorpha from the vertebrate-rich Plattenkalk of Pietraroia (Lower Albian, southern Apennines, Italy). *Zoological Journal of the Linnean Society*. zoj_718 199.. 227.

Carpenter K., & Lindsey D. (1980). The dentary of *Brachychampsa montana* Gilmore (Alligatorinae; Crocodylidae), a Late Cretaceous turtle-eating alligator. *Journal of Paleontology*, 1213-1217.

Di Celma C., Malinverno E., Gariboldi K., Gioncada A., Rustichelli A., Pierantoni P.P., ... & Bianucci G. (2016). Stratigraphic framework of the late Miocene to Pliocene Pisco Formation at Cerro Colorado (Ica Desert, Peru). *Journal of Maps*, *12*(3), 515-529.

Cidade G.M., Fortier D., & Hsiou A.S. (2019a). Taxonomic and phylogenetic review of *Necrosuchus ionensis* (Alligatoroidea: Caimaninae) and the early evolution and radiation of caimanines. *Zoological Journal of the Linnean Society*. https://doi.org/https://doi.org/10.1093/zoolinnean/zlz051

Clark J.M., & Norell M. (1992). The Early Cretaceous crocodylomorph *Hylaeochampsa vectiana* from the wealden of the Isle of Wight. *American Museum Novitates*, 3032, 1–19.

Conrad J.L., Jenkins K., Lehmann T., Manthi F.K., Peppe D.J., Nightingale S., Cossette A., Dunsworth H.M., Harcourt-Smith W.E., & Mcnulty K.P. (2013). New specimens of ‘*Crocodylus*’ *pigotti* (Crocodylidae) from Rusinga Island, Kenya, and generic reallocation of the species. *Journal of Vertebrate Paleontology*, 33(3), 629–646. 11

Collareta A., Di Celma C., Bosio G., Pierantoni P.P., Malinverno E., Lambert O., ... & Bianucci G. (2021). Distribution and paleoenvironmental framework of middle Miocene marine vertebrates along the western side of the lower Ica Valley (East Pisco Basin, Peru). *Journal of Maps*, 17(2), 7-17.

Cossette A.P., & Brochu C.A. (2018). A new specimen of the alligatoroid *Bottosaurus harlani* and the early history of character evolution in alligatorids. *Journal of Vertebrate Paleontology*, 38(4), (1)–(22).

Cossette A.P., & Brochu C.A. (2020). A systematic review of the giant alligatoroid *Deinosuchus* from the Campanian of North America and its implications for the relationships at the root of Crocodylia. *Journal of Vertebrate Paleontology*, 40(1), e1767638.

Cossette A.P. (2021). A new species of *Bottosaurus* (Alligatoroidea: Caimaninae) from the Black Peaks Formation (Palaeocene) of Texas indicates an early radiation of North American caimanines. *Zoological Journal of the Linnean Society*, 191(1), 276-301.

Delfino M., Codrea V., Folie A., Dica P., Godefroit P., & Smith T. (2008). A complete skull of *Allodaposuchus precedens* Nopcsa, 1928 (Eusuchia) and a reassessment of the morphology of the taxon based on the Romanian remains. *Journal of Vertebrate Paleontology*, 28(1), 111–122.

Delfino M., Piras P., & Smith T. (2005). Anatomy and phylogeny of the gavialoid crocodylian *Eosuchus lerichei* from the Paleogene of Europe. *Acta Palaeontologica Polonica*, 50(3), 565–580.

Delfino M., & Smith T. (2009). A reassessment of the morphology and taxonomic status of ‘*Crocodylus*’ *depressifrons* (Crocodylia, Crocodyloidea) based on

the Early Eocene remains from Belgium. *Zoological Journal of the Linnean Society*, 156(1), 140–167.

Delfino M., & Smith T. (2012). Reappraisal of the morphology and phylogenetic relationships of the middle Eocene alligatoroid *Diplocynodon deponiae* (Frey, Laemmert, and Riess, 1987) based on a three-dimensional specimen. *Journal of Vertebrate Paleontology*, 32(6), 1358–1369.

Delfino M., & De Vos J. (2014). A giant crocodile in the Dubois Collection from the Pleistocene of Kali Gedeh (Java). *Integrative zoology*, 9:141–147.

Delfino M., Martin J.E., De Broin F.D.L., & Smith T. (2017). Evidence for a pre-PETM dispersal of the earliest European crocodyloids. *Historical Biology*, 31(7), 845-852.

Dridi J. (2018). New fossils of the giant pholidosaurid genus *Sarcosuchus* from the Early Cretaceous of Tunisia. *Journal of African Earth Sciences*, 147, 268-280.

Erickson B.R. 1972. *Albertochampsa langstoni*, gen. et sp. nov., a new alligator from the Cretaceous of Alberta. *Scientific Publications of the Science Museum of Minnesota*, New Series 2, 1–13.

Erickson B.R. (1976). Osteology of the early eusuchian crocodile *Leidyosuchus formidabilis*, sp. nov. *Monographs of the Science Museum of Minnesota (Paleontology*), (2).

Erickson B.R. (1982). *Wannaganosuchus*, a new alligator from the Paleocene of North America. *Journal of Paleontology*, 492–506.

Erickson G.M., & Brochu C.A. (1999). How the ‘terror crocodile grew so big. *Nature*, 398:205.

Fortier D.C., De Souza-Filho J.P., Guilherme E., Maciente A.A., & Schultz C.L. (2014). A new specimen of *Caiman brevirostris* (Crocodylia, Alligatoridae) from the Late Miocene of Brazil. *Journal of Vertebrate Paleontology*, 34(4), 820–834.

Gilmore C.W. (1910). *Leidyosuchus sternbergii*, a new species of crocodile from the Ceratops Beds of Wyoming. Proceedings of the United States National Museum, 38(1762), 485–502.

Gilmore C.W. (1911). A new fossil alligator from the Hell Creek beds of Montana. *Proceedings of the United States National Museum* 41(1860), 297–-302

Gilmore C.W. (1946). A new crocodilian from the Eocene of Utah. *Journal of Paleontology*, 20, 62–67.

Ginsburg L., & Buffetaut E. (1978). *Euthecodon arambourgi* n. sp., et l'évolution du genre *Euthecodon*, Crocodilien du Néogène d'Afrique. *Géologie méditerranéenne*, 5(2), 291–301.

Godoy P.L., Cidade G.M., Montefeltro F.C., Langer M.C., & Norell M.A. (2021). Redescription and phylogenetic affinities of the caimanine *Eocaiman cavernensis* (Crocodylia, Alligatoroidea) from the Eocene of Argentina. *Papers in Palaeontology*, 7(3), 1205-1231.

Hastings A.K., Reisser M., & Scheyer T.M. (2016). Character evolution and the origin of Caimaninae (Crocodylia) in the New World Tropics: new evidence from the Miocene of Panama and Venezuela. *Journal of Paleontology*, 90(2), 317–332.

Head J.J. 2001. Systematics and body size of the gigantic, enigmatic crocodyloid *Rhamphosuchus crassidens*, and the faunal history of Siwalik Group (Miocene) crocodylians. *Journal of Vertebrate Paleontology*, 21:A59.

Hester D.A. (2018). *A review of the Paleogene eusuchian crocodyliform Borealosuchus wilsoni (Mook, 1959) from western North America* (Doctoral dissertation, The University of Iowa).

Iijima M., Takahashi K., & Kobayashi Y. (2016). The oldest record of *Alligator sinensis* from the Late Pliocene of Western Japan, and its biogeographic implication. *Journal of Asian Earth Sciences*, *124*, 94-101.

Kälin J.A. 1939. Ein extrem kurzschnauziger Crocodilide aus den Phosphoriten des Quercy, *Arambourgia* (nov. gen.) *gaudryi* de Stefano. *Abhandlungen der Schweizerischen Palaeontologischen Gesellschaft*, 62, 1-18.

Kern A.K., Gross M., Galeazzi C.P., Pupim F.N., Sawakuchi A.O., Almeida R.P., ... & Basei M.A. (2020). Re-investigating Miocene age control and paleoenvironmental reconstructions in western Amazonia (northwestern Solimões Basin, Brazil). *Palaeogeography, Palaeoclimatology, Palaeoecology*, 545, 109652.

Khalifa M.A., & Catuneanu O. (2008). Sedimentology of the fluvial and fluvio-marine facies of the Bahariya Formation (early Cenomanian), Bahariya Oasis, Western Desert, Egypt. *Journal of African Earth Sciences*, 51(2), 89-103.

Kobayashi Y., Tomida Y., Kamei T., & Eguchi T. (2006). Anatomy of a Japanese tomistomine crocodylian, *Toyotamaphimeia machikanensis* (Kamei et Matsumoto, 1965), from the middle Pleistocene of Osaka Prefecture: the reassessment of its

phylogenetic status within Crocodylia. *National Science Museum Monographs*, 35, 1–121.

Koken E. (1888). *Thoracosaurus macrorhynchus* Bl. aus der Tuffkreide von Maastricht. *Zeitschrift der Deutschen Geologischen Gesellschaft*, 754–773.

Kraus R. (1998). The cranium of *Piscogavialis jugaliperforatus* n. gen., n. sp. (Gavialidae, Crocodylia) from the Miocene of Peru. *Paläontologische Zeitschrift*, 72(3–4), 389–405.

Langston W. (1965). Fossil crocodilians from Colombia and the Cenozoic history of the Crocodilia in South America. *University of California Publications in Geological Sciences*, 52.

Langston Jr W. (1975). The ceratopsian dinosaurs and associated lower vertebrates from the St. Mary River Formation (Maestrichtian) at Scabby Butte, southern Alberta. *Canadian Journal of Earth Sciences*, 12(9), 1576–1608.

Li C., Wu X.-C., & Rufolo S.J. (2019). A new crocodyloid (Eusuchia: Crocodylia) from the Upper Cretaceous of China. *Cretaceous Research*, 94, 25–39.

Lindblad K.T., Moreno-Bernal J.W., McKellar R.C., & Velez M.I. (2022). The Northern Crocodile: first report of *Borealosuchus* (Eusuchia; Crocodylia) from Saskatchewan's lower Ravenscrag Formation (earliest Paleocene) with implications for biogeography. *Canadian Journal of Earth Sciences*, 59(9), 623–638.

Lucas S.G., & Estep J.W. (2000). Osteology of *Allognathosuchus mooki* Simpson, a Paleocene crocodilian from the San Juan Basin, New Mexico, and the monophyly of *Allognathosuchus*. *New Mexico Museum of Natural History and Science Bulletin*, 16, 155–168.

Ludwig R. (1877). Fossile Crocodiliden aus der Terti¨arformation des mainzer Beckens (Vol. 3). *Paleontographica Supplement*.

Lull R.S. (1944). Fossil gavials from north India. *American Journal of Science*, 242(8), 417–430.

Martin J.E. (2007). New material of the Late Cretaceous globidontan *Acynodon iberoccitanus* (Crocodylia) from southern France. *Journal of Vertebrate Paleontology*, 27(2), 362–372.

Martin J.E. (2019). The taxonomic content of the genus Gavialis from the Siwalik Hills of India and Pakistan. *Papers in Palaeontology*, 5(3), 483-497.

Martin J.E., & Lauprasert K. (2010). A new primitive alligatorine from the Eocene of Thailand: relevance of Asiatic members to the radiation of the group. *Zoological Journal of the Linnean Society*, 158(3), 608-628.

Martin J.E., Delfino M., Garcia G., Godefroit P., Berton S., & Valentin X. (2016). New specimens of *Allodaposuchus precedens* from France: intraspecific variability and the diversity of European Late Cretaceous eusuchians. *Zoological Journal of the Linnean Society*, 176(3), 607–631.

Martin J.E., Lauprasert K., Buffetaut E., Liard R., & Suteethorn V. (2014a). A large pholidosaurid in the Phu Kradung Formation of north-eastern Thailand. *Palaeontology*, 57:757-769.

Martin J.E., Smith T., de Lapparent de Broin F., Escuillié F., & Delfino M. (2014b). Late Palaeocene eusuchian remains from Mont de Berru, France, and the origin of the alligatoroid *Diplocynodon*. *Zoological Journal of the Linnean Society*, 172(4), 867–891.

Martin J.E., Antoine P-O., Perrier V., Welcomme J-L., Metais G., et al. (2019). A large crocodyloid from the Oligocene of the Bugti Hills, Pakistan. *Journal of Vertebrate Paleontology*, 39 (4).

Martin J.E., Smith T., Salaviale C., Adrien J., & Delfino M. (2020). Virtual reconstruction of the skull of *Bernissartia fagesii* and current understanding of the neosuchian–eusuchian transition. *Journal of Systematic Palaeontology*, 18(13), 1079-1101.

Massonne T., Vasilyan D., Rabi M., & Böhme M. (2019). A new alligatoroid from the Eocene of Vietnam highlights an extinct Asian clade independent from extant *Alligator sinensis*. *PeerJ*, 7, e7562.

Medina, C.J. (1976). Crocodilian from the Late Tertiary of Northwestern Venezuela: *Melanosuchus fisheri* sp. nov. *Breviora,* 438: 1–14.

Molnar, R. E. (1981). Pleistocene ziphodont crocodilians of Queensland. *Records of the Australian Museum*, 33(19), 803–834.

Mook C.C., & Thomson A. (1923). A new species of Alligator from the Snake Creek beds. By order of the Trustees of The American Museum of Natural History.

Mook C.C. (1941a). A new crocodilian, *Hassiacosuchus kayi*, from the Bridger Eocene beds of Wyoming. *Annals of Carnegie Museum*, 28, 207–-220.

Mook C.C. (1941b). A new crocodilian from the Lance Formation. *American Museum Novitates*, 1128, 1–5.

Mook C.C. (1959). A new species of fossil crocodile of the genus *Leidyosuchus* from the Green River beds. *American Museum Novitates*, 1933, 1–6.

Mook C.C. (1961). Notes on the skull characters of *Allognathosuchus polyodon*. *American Museum Novitates*, 2072, 1–5.

Mook C.C., & Thomson, A. (1962). A new species of *Brachyuranochampsa* (Crocodilia) from the Bridger beds of Wyoming. *American Museum novitates*; no. 2079.

More S., Paruya D.K., Taral S., Chakraborty T., & Bera S. (2016). Depositional environment of Mio-Pliocene Siwalik sedimentary strata from the Darjeeling Himalayan Foothills, India: a palynological approach. *PLoS One*, 11(3), e0150168.

Müller L. (1927). Ergebnisse der Forschungsreisen Prof. E. Stromers in den Wüsten Ägypteus. Abhandlungen Bayerisch Akademie der Wissenschaften. *Mathematisch-Naturwissenschaftelischt Abtelund*, 31, 1–97.

Myrick A.C. (2001). *Thecachampsa antiqua* (Leidy, 1852) (Crocodylidae, Thoracosaurinae) from fossil marine deposits at Lee Creek Mine, Aurora, North Carolina, USA. *Smithsonian Contributions to Paleobiology*, 90, 219-225.

Narvaez I., Brochu C.A., Escaso F., Perez-Garcia A., & Ortega F. (2015). New crocodyliforms from southwestern Europe and definition of a diverse clade of European Late Cretaceous basal eusuchians. *PLoS One*, 10(11), e0140679.

Nicholl C.S., Rio J.P., Mannion P.D., & Delfino M. (2020). A re-examination of the anatomy and systematics of the tomistomine crocodylians from the Miocene of Italy and Malta. *Journal of Systematic Palaeontology*, *18*(22), 1853-1889.

Norell M., Clark J.M., & Hutchison J.H. (1994). The Late Cretaceous alligatoroid *Brachychampsa montana* (Crocodylia): new material and putative relationships. *American Museum Novitates*, 3116, 1–26.

Nutz A., Schuster M., Barboni D., Gassier G., Van Bocxlaer B., Robin C., ... & Rubino J.L. (2020). Plio-Pleistocene sedimentation in West Turkana (Turkana depression, Kenya, East African rift system): paleolake fluctuations, paleolandscapes and controlling factors. *Earth-Science Reviews*, 211, 103415.

Ösi A. (2008). Cranial osteology of *Iharkutosuchus makadii*, a Late Cretaceous basal eusuchian crocodyliform from Hungary. *Neues Jahrbuch für Geologie und Paläontologie-Abhandlungen*, 248(3), 279–299. 14

Ösi, A., & Weishampel, D. B. (2009). Jaw mechanism and dental function in the Late Cretaceous basal eusuchian *Iharkutosuchus*. *Journal of Morphology*, 270(8), 903–920.

Paiva A.L.S., Godoy P.L., Souza R.B., Klein W., & Hsiou A.S. (2022). Body size estimation of Caimaninae specimens from the Miocene of South America. *Journal of South American Earth Sciences*, 118, 103970.

Pinheiro A.E., Fortier D.C., Pol D., Campos D.A., & Bergqvist L.P. (2013). A new *Eocaiman* (Alligatoridae, Crocodylia) from the Itaboraí Basin, Paleogene of Rio de Janeiro, Brazil. *Historical Biology*, 25(3), 327-337.

Piras P., & Buscalioni A.D. (2006). *Diplocynodon muelleri* comb. nov., an Oligocene diplocynodontine alligatoroid from Catalonia (Ebro Basin, Lleida province, Spain). *Journal of Vertebrate Paleontology*, 26(3), 608–620.

Pol D., Turner A.H., & Norell M.A. (2009). Morphology of the Late Cretaceous crocodylomorph Shamosuchus djadochtaensis and a discussion of neosuchian phylogeny as related to the origin of Eusuchia. *Bulletin of the American Museum of Natural History*, 324, 1–104.

Puértolas E., Canudo J.I., & Cruzado-Caballero P. (2011). A new crocodylian from the Late Maastrichtian of Spain: implications for the initial radiation of crocodyloids. *PLoS One*, *6*(6), e20011.

Puértolas-Pascual E., Canudo J.I., & Moreno-Azanza M. (2014). The eusuchian crocodylomorph *Allodaposuchus subjuniperus* sp. nov., a new species from the latest Cretaceous (upper Maastrichtian) of Spain. *Historical Biology*, *26*(1), 91-109.

Riff D., & Aguilera O.A. (2008). The world's largest gharials *Gryposuchus*: description of *G. croizati* n. sp. (Crocodylia, Gavialidae) from the Upper Miocene Urumaco Formation, Venezuela. *Paläontologische Zeitschrift*. 82:178–195.

Rio J.P., Mannion P.D., Tschopp E., Martin J.E., & Delfino M. (2020). Reappraisal of the morphologyand phylogenetic relationships of the alligatoroid crocodylian *Diplocynodon hantoniensis* from the late Eocene of the United Kingdom. *Zoological Journal of the Linnean Society*, 188(2),579–629.

Rio J.P., & Mannion P.D. 2021. Phylogenetic analysis of a new morphological dataset elucidates the evolutionary history of Crocodylia and resolves the long-standing gharial problem. *PeerJ*, 9, e12094.

Ristevski J., Price G.J., Weisbecker V., & Salisbury S.W. (2021). First record of a tomistomine crocodylian from Australia. *Scientific reports*, 11(1), 1-14.

Rogers J.V. (2003). *Pachycheilosuchus trinquei*, a new procoelous crocodyliform from the Lower Cretaceous (Albian) Glen Rose Formation of Texas. *Journal of Vertebrate Paleontology*, 23(1), 128-145.

Rossmann T. (1998). Studien an känozoischen Krokodilen: 2. Taxonomische Revision der Familie Pristichampsidae Efimov (Crocodilia: Eusuchia). *Neues Jahrbuch für Geologie und Paläontologie Abhandlungen*, 210(1), 85–128.

Salas-Gismondi R., Flynn J.J., Baby P., Tejada-Lara J.V., Wesselingh F.P., & Antoine P.-O. (2015). A Miocene hyperdiverse crocodylian community reveals peculiar trophic dynamics in proto- Amazonian mega-wetlands. *Proceedings of the Royal Society B: Biological Sciences*, 282(1804), 20142490.

Salas-Gismondi R., Flynn J.J., Baby P., Tejada-Lara J.V., Claude J., & Antoine P.-O. (2016). A new 13 million year old gavialoid crocodylian from proto-Amazonian mega-wetlands reveals parallel evolutionary trends in skull shape linked to longirostry. *PloS One*, 11(4), e0152453.

Salisbury S.W., & Willis P.M.A. (1996). A new crocodylian from the early Eocene of south-eastern Queensland and a preliminary investigation of the phylogenetic relationships of crocodyloids. *Alcheringa*, 20(3), 179–226.

Shan, H. Y., Wu, X. C., Sato, T., Cheng, Y. N., & Rufolo, S. (2021). A new alligatoroid (Eusuchia, Crocodylia) from the Eocene of China and its implications for the relationships of Orientalosuchina. *Journal of Paleontology*, *95*(6), 1321-1339.

Scheyer T.M., Aguilera O.A., Delfino M., Fortier D.C., Carlini A.A., Sánchez R., ... & Sánchez-Villagra M.R. (2013). Crocodylian diversity peak and extinction in the late Cenozoic of the northern Neotropics. *Nature communications*, 4(1), 1907.

Scheyer T.M., & Delfino M. (2016). The late Miocene caimanine fauna (Crocodylia: Alligatoroidea) of the Urumaco Formation, Venezuela. *Palaeontologia Electronica*, 19(3), 1–57. 15

Scheyer T.M., Hutchinson J.R., Strauss O., Delfino M., Carrillo-Briceño J.D., Sánchez R., & Sánchez-Villagra M.R. (2019). Giant extinct caiman breaks constraint on the axial skeleton of extant crocodylians. *Elife*, 8, e49972.

Schmidt K.P. (1941). A new fossil alligator from Nebraska. *Fieldiana*, 8, 27–32.

Sémah A.M., Sémah F., Djubiantono T., & Brasseur B. (2010). Landscapes and Hominids' environments: changes between the Lower and the Early Middle Pleistocene in Java (Indonesia). *Quaternary International*, 223, 451-454.

Sémah A.M., Sémah F., Moigne A.M., Ingicco T., Purnomo A., Simanjuntak T., & Widianto H. (2016). The palaeoenvironmental context of the Palaeolithic of Java: A brief review. *Quaternary International*, 416, 38-45.

Sereno P.C., Larsson H.C., Sidor C.A., & Gado B. (2001). The giant crocodyliform *Sarcosuchus* from the Cretaceous of Africa. *Science* 294:1516-1519.

Shan H.-Y., Cheng Y.-N. & Wu X.-C. (2013). The first fossil skull of Alligator sinensis from the Pleistocene, Taiwan, with a paleogeographic implication of the species. *Journal of Asian Earth Sciences*, 69, 17-25.

Shan H.-Y., Wu X.-C., Cheng Y.-N., & Sato T. (2017). *Maomingosuchus petrolica*, a restudy of 'Tomistoma' petrolica Yeh, 1958. *Palaeoworld*, 26(4), 672–690.

Shan H.Y., Wu, X.-C., Sato T., Cheng Y.N., & Rufolo S. (2021). A new alligatoroid (Eusuchia, Crocodylia) from the Eocene of China and its implications for the relationships of Orientalosuchina. *Journal of Paleontology*, 95(6), 1321-1339.

Simpson G.G. (1933). A new crocodilian from the *Notostylops* beds of Patagonia. *American Museum Novitates*, 623, 1–9.

Smith J.B., Lamanna M.C., Lacovara K.J., Dodson P., Smith J.R., Poole J.C., ... & Attia Y. (2001). A giant sauropod dinosaur from an Upper Cretaceous mangrove deposit in Egypt. *Science*, 292(5522), 1704-1706.

Sookias R.B. (2020). Exploring the effects of character construction and choice, outgroups and analytical method on phylogenetic inference from discrete characters in extant crocodilians. *Zoological Journal of the Linnean Society*, 189(2), 670-699.

Sternberg C.M. (1932). A new fossil crocodile from Saskatchewan. *The Canadian Field-Naturalist*, 44, 128–133.

Stocker M.R., Brochu C.A., & Kirk E.C. (2021). A new caimanine alligatorid from the Middle Eocene of Southwest Texas and implications for spatial and temporal shifts in Paleogene crocodyliform diversity. *PeerJ*, 9, e10665.

Storrs G.W. (2003). Late Miocene–Early Pliocene Crocodilian Fauna of Lothagam, Southwest Turkana Basin, Kenya. In *Lothagam: the dawn of humanity in Eastern Africa* . Columbia University Press. 137–160.

Stromer E. (1925). Ergebnisse der Forschungsreisen Prof. E. Stromers in den Wüsten Ägyptens. II. Wirbeltier-Reste der Baharije-Stufe (unterstes Cenoman). 7. Stomatosuchus inermis Stromer, ein schwach bezahnter Krokodilier und 8. Ein Skelettrest des Pristiden Onchopristis numidus Haug sp. Abhandlungen der Bayerischen Akademie der Wissenschaften, Mathematisch-naturwissenschaftliche Abteilung. 30:1–22.

Sullivan R.M., & Lucas S.G. (1986). Annotated list of lower vertebrates from the Paleocene Nacimiento Formation (Puercan-Torrejonian), San Juan Basin, New Mexico. *Journal of Herpetology*, 20(2), 202-209.

Sullivan R.M., & Lucas S.G. (2003). *Brachychampsa montana* Gilmore (Crocodylia, Alligatoroidea) from the Kirtland Formation (Upper Campanian), San Juan Basin, New Mexico. *Journal of Vertebrate Paleontology*, 23(4), 832-841.

Toula F., & Kail J.A. (1885). Über einen Krokodil-Schädel aus den Tertiär Ablagerungen von Eggenburg in Niederösterreich: eine paläontologische Studie. *Denkschriften der Keiserlichen Akademie der Wissenschaften, Mathematisch-Naturwissenschatflliche Klasse*, 50.

Turner A.H. (2015). A review of *Shamosuchus* and *Paralligator* (Crocodyliformes, Neosuchia) from the Cretaceous of Asia. *PLoS One*, 10(2), e0118116.

Vasse D. (1992). Un crâne d'*Asiatosuchus germanicus* du Lutétien d'Issel (Aude). Bilan sur le genre *Asiatosuchus* en Europe. *Geobios*, *25*(2), 293-304.

Vieira L.G., Santos A.L.Q., Lima F.C., de Mendonça S.H.S.T., Menezes L.T., & Sebben A. (2016). Ontogeny of the appendicular skeleton in *Melanosuchus niger* (Crocodylia: Alligatoridae). *Zoological Science*, *33*(4), 372-383.

Voiculescu-Holvad C. (2022). Historical material of cf. *Thoracosaurus* from the Maastrichtian of Denmark provides new insight into the K/Pg distribution of Crocodylia. *Cretaceous Research*, *139*, 105309.

Walter J., Darlim G., Massonne T., Aase A., Frey E., & Rabi M. (2022). On the origin of Caimaninae: insights from new fossils of *Tsoabichi greenriverensis* and a review of the evidence. *Historical Biology*, 34(4), 580-595.

Wang Y.-Y., Sullivan C., & Liu J. (2016). Taxonomic revision of *Eoalligator* (Crocodylia, Brevirostres) and the paleogeographic origins of the Chinese alligatoroids. *PeerJ*, 4, e2356.

Wassersug R.J., & Hecht M.K. (1967). The status of the crocodylid genera *Procaimanoidea* and *Hassiacosuchus* in the New World. *Herpetologica*, *23*(1), 30-34.

Weems R E. (2018). Crocodilians of the Calvert Cliffs. In S. J. Godfrey (Ed.), The Geology and Vertebrate Paleontology of Calvert Cliffs, Maryland, USA . *Smithsonian Institution Scholarly Press.* 213–240

White T.E. (1942). A new alligator from the Miocene of Florida. *Copeia*, 1942(1), 3–7.

Williamson T.E. 1996. ?*Brachychampsa sealeyi*, sp. nov., (Crocodylia, Alligatoroidea) from the Upper Cretaceous (lower Campanian) Menefee Formation, northwestern New Mexico. *Journal of Vertebrate Paleontology*, 16, 421–-431.

Willis P.M.A., & Molnar R.E. (1991). A new middle Tertiary crocodile from Lake Palankarinna, South Australia. *Records of the South Australian Museum*, 25(1), 39–55.

Willis P.M. (1993). *Trilophosuchus rackhami* gen. et sp. nov., a new crocodilian from the early Miocene limestones of Riversleigh, northwestern Queensland. *Journal of Vertebrate Paleontology*, 13(1), 90–98.

Wu X.-C., Brinkman D.B., & Russell A.P. (1996). A new alligator from the Upper Cretaceaous of Canada and the relationship of early eusuchians. *Palaeontology*, 39, 351-376.

Wu X.-C., Brinkman D.B., & Fox R.C. (2001a). A new crocodylian (Archosauria) from the basal Paleocene of the Red Deer River Valley, southern Alberta. *Canadian Journal of Earth Sciences*, 38(12), 1689-1704.

Wu X.-C., Russell A.P., & Brinkman D.B. (2001b). A review of *Leidyosuchus canadensis* Lambe, 1907 (Archosauria: Crocodylia) and an assessment of cranial variation based upon new material. *Canadian Journal of Earth Sciences*, 38(12), 1665–1687.

Wu X.-C., Li C., Wang Y.-Y. (2018). Taxonomic reassessment and phylogenetic test of *Asiatosuchus nanlingensis* Young, 1964 and *Eoalligator chunyii* Young, 1964. *Vertebrata PalAsiatica*, 56(2): 137–146

Zangerl R. (1944). *Brachyuranochampsa eversolei*, gen. et sp. nov., a new crocodilian from the Washakie Eocene of Wyoming. *Annals of the Carnegie Museum*, 30, 77–84.
