# Supplementary material for: Expanded phylogeny elucidates Deinosuchus relationships, crocodylian osmoregulation and body-size evolution
Source: Commun Biol. 2025 Apr 23;8:611. doi: 10.1038/s42003-025-07653-4 (PMC12018936; doi:10.1038/s42003-025-07653-4)
Supplement: Supplementary file 2 — Description of Additional Supplementary Materials [file 42003_2025_7653_MOESM2_ESM.pdf]

Description of Additional Supplementary Files

File name: Supplementary Data 1

Description: The folder 'Supplementary Data 1' contains the character-taxon dataset used for the phylogenetic analyses as well as the TNT files. These are the source data behind the graphs in Figure 1, 2 and 4B.

File name: Supplementary Data 2

Description: The folder 'Supplementary Data 2' contains all the primary data, code and files used to determine the body size estimates (folders 1. and 2.), along with the raw data and results (folder 3.). These are the source data behind the graphs in Figure 3.
