# Supplementary material for: Expanded phylogeny elucidates Deinosuchus relationships, crocodylian osmoregulation and body-size evolution
Source: Commun Biol. 2025 Apr 23;8:611. doi: 10.1038/s42003-025-07653-4 (PMC12018936; doi:10.1038/s42003-025-07653-4)
Supplement: Supplementary file 4 — Supplementary Data 2 [file 42003_2025_7653_MOESM4_ESM.zip › Supplementary Data 2/2. R Code - body size/outputBayesianModel.pdf]

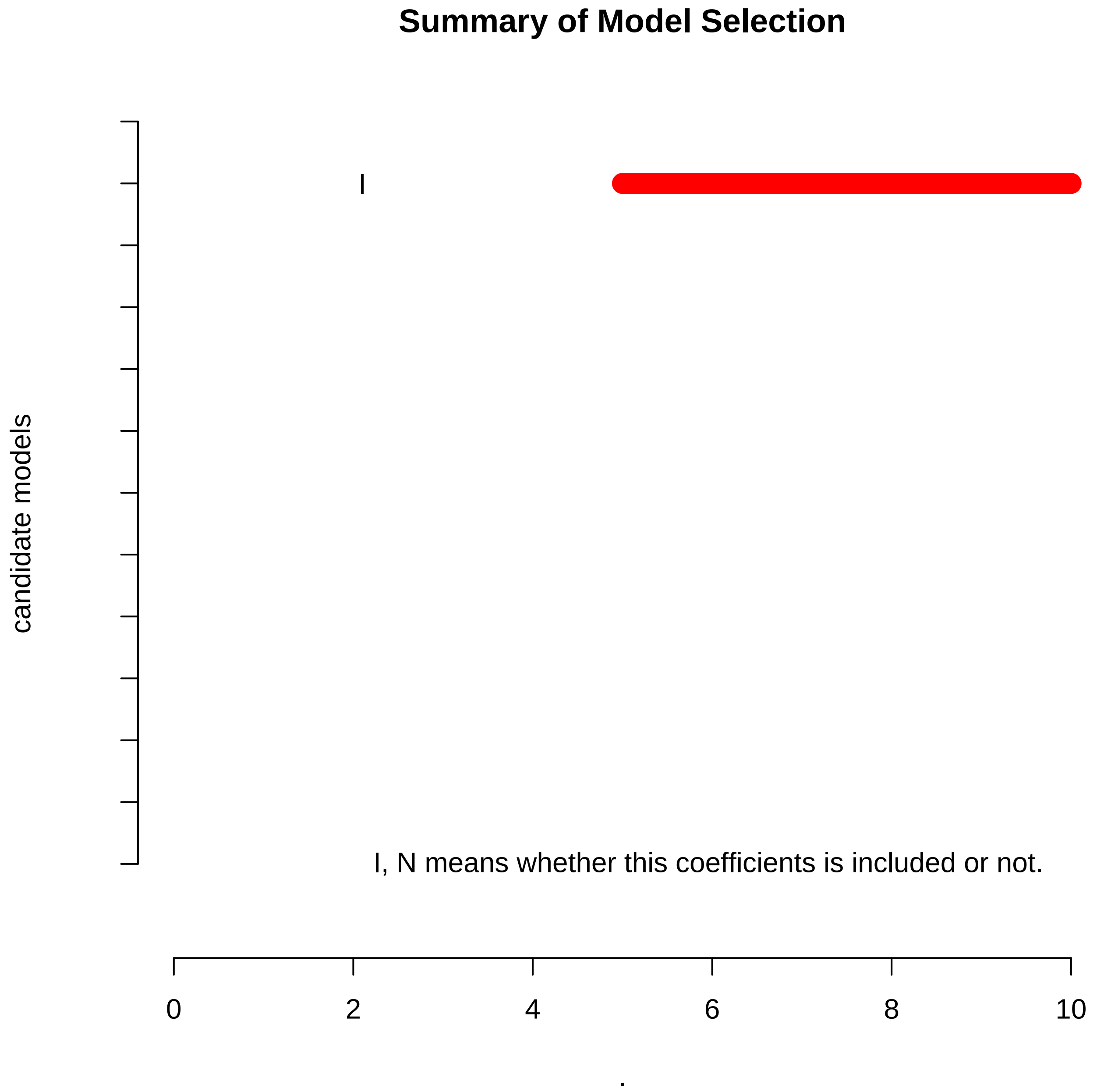

Summary of Model Selection
I
I, N means whether this coefficients is included or not.
candidate models
0
2
4
6
8
10
.

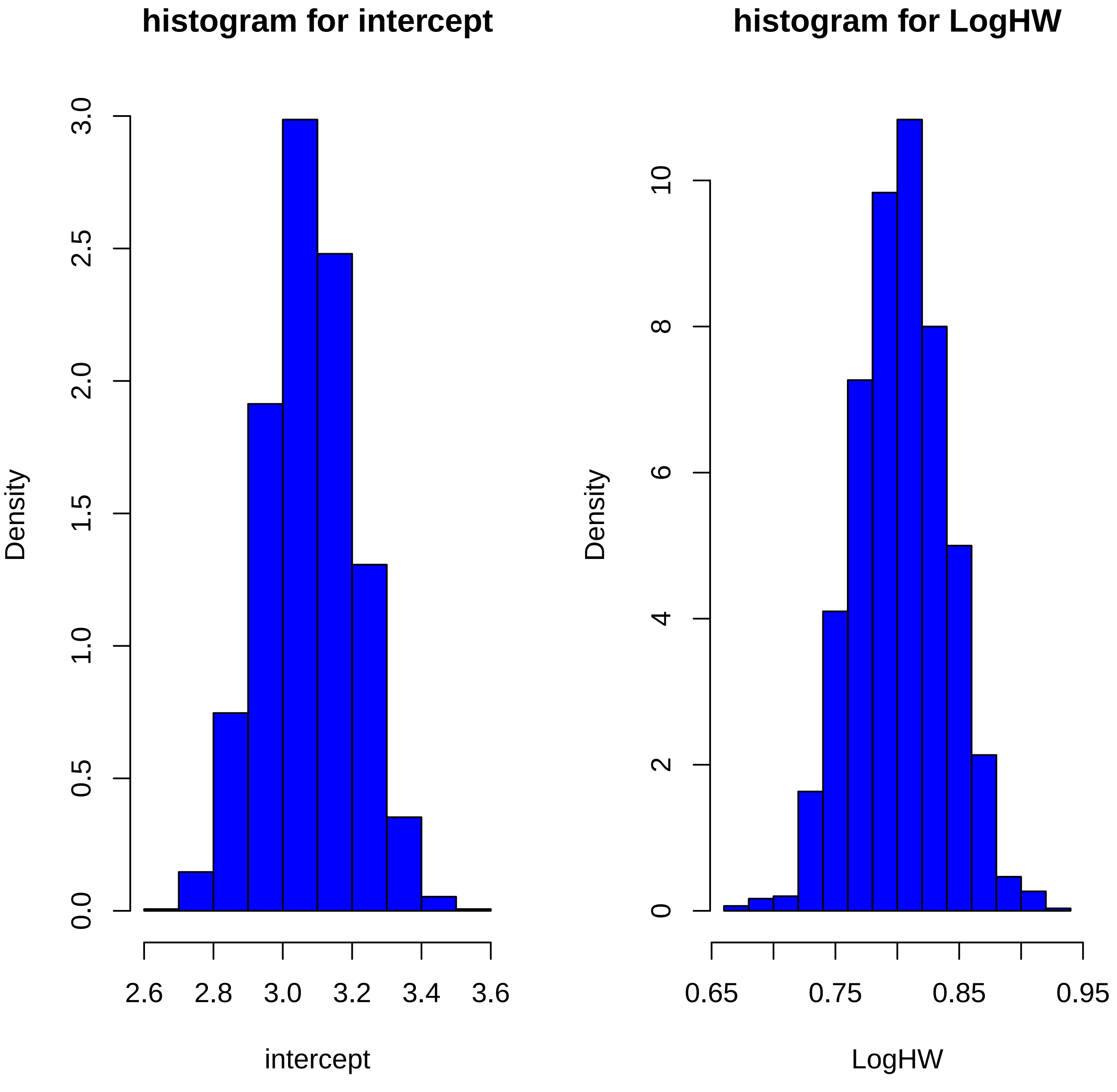

histogram for intercept
Density
0.0
0.5
1.0
1.5
2.0
2.5
3.0
2.6
2.8
3.0
3.2
3.4
3.6
intercept
histogram for LogHW
Density
0
2
4
6
8
10
0.65
0.75
0.85
0.95
LogHW

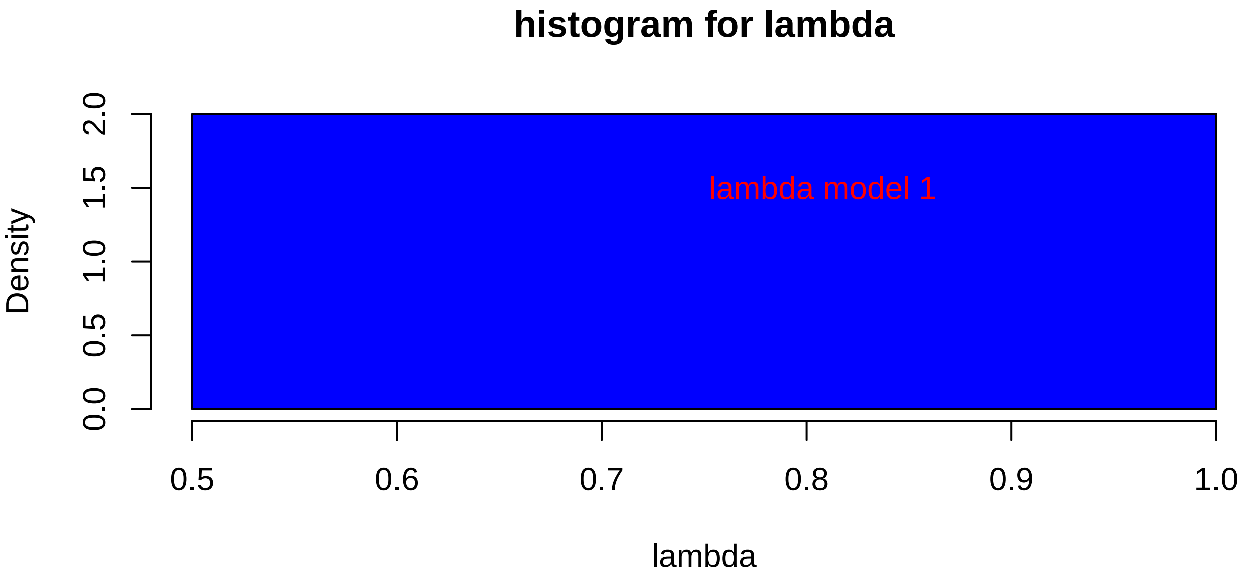

histogram for lambda
lambda model 1
Density
0.0
0.5
1.0
1.5
2.0
0.5
0.6
0.7
0.8
0.9
1.0
lambda

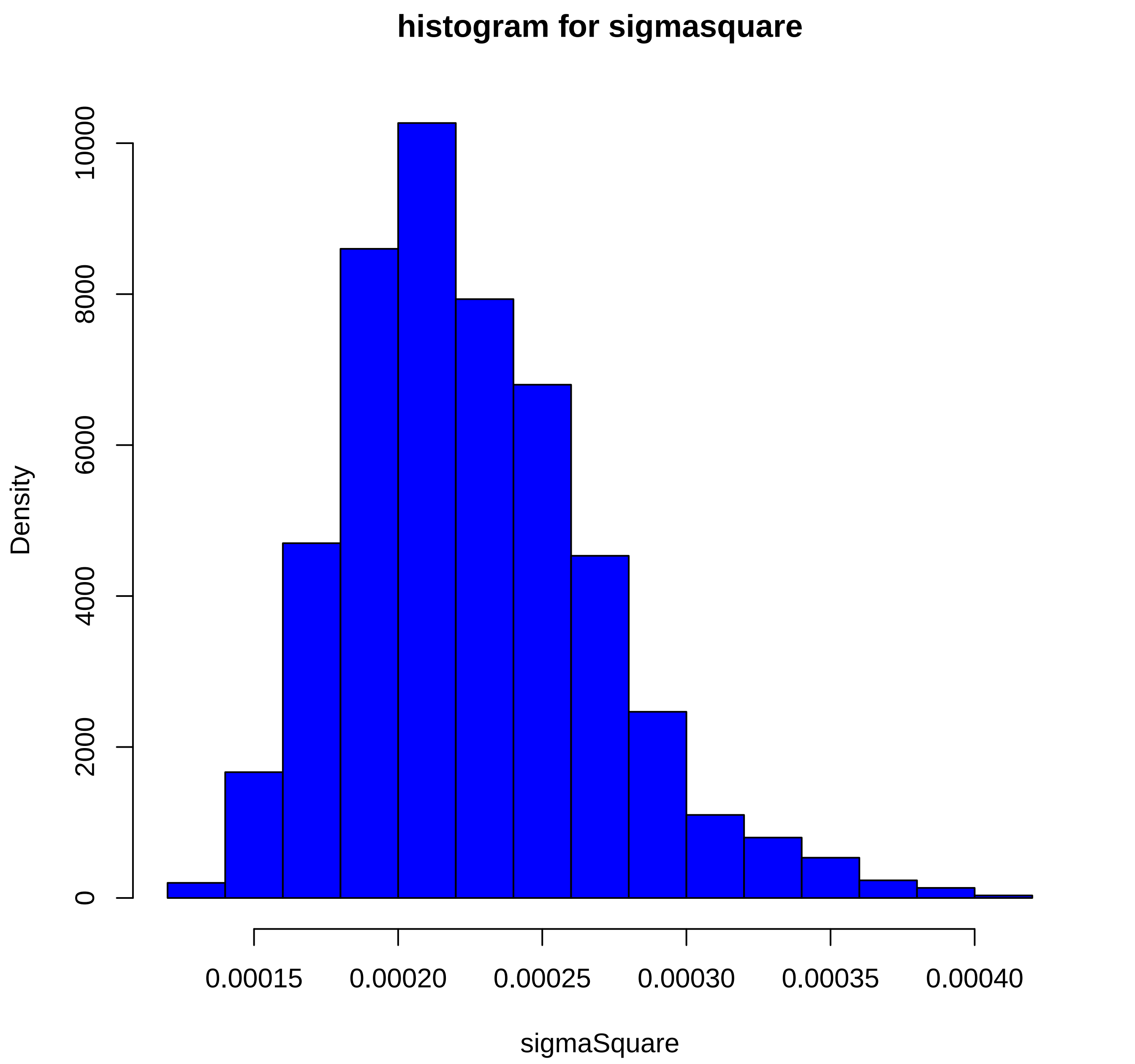

histogram for sigmasquare
Density
0
2000
4000
6000
8000
10000
0.00015
0.00020
0.00025
0.00030
0.00035
0.00040
sigmaSquare

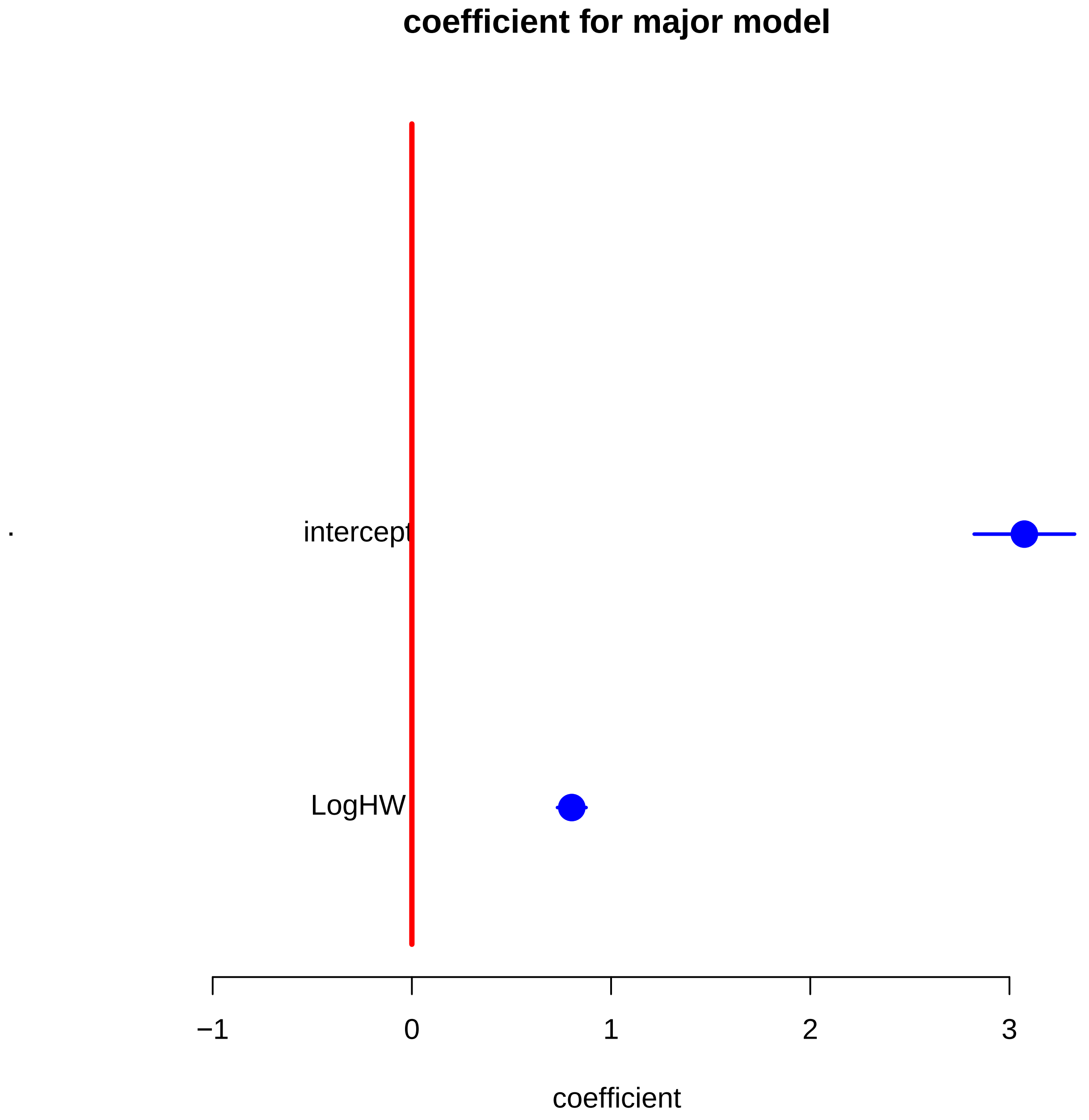

coefficient for major model
intercept
LogHW
−1
0
1
2
3
coefficient

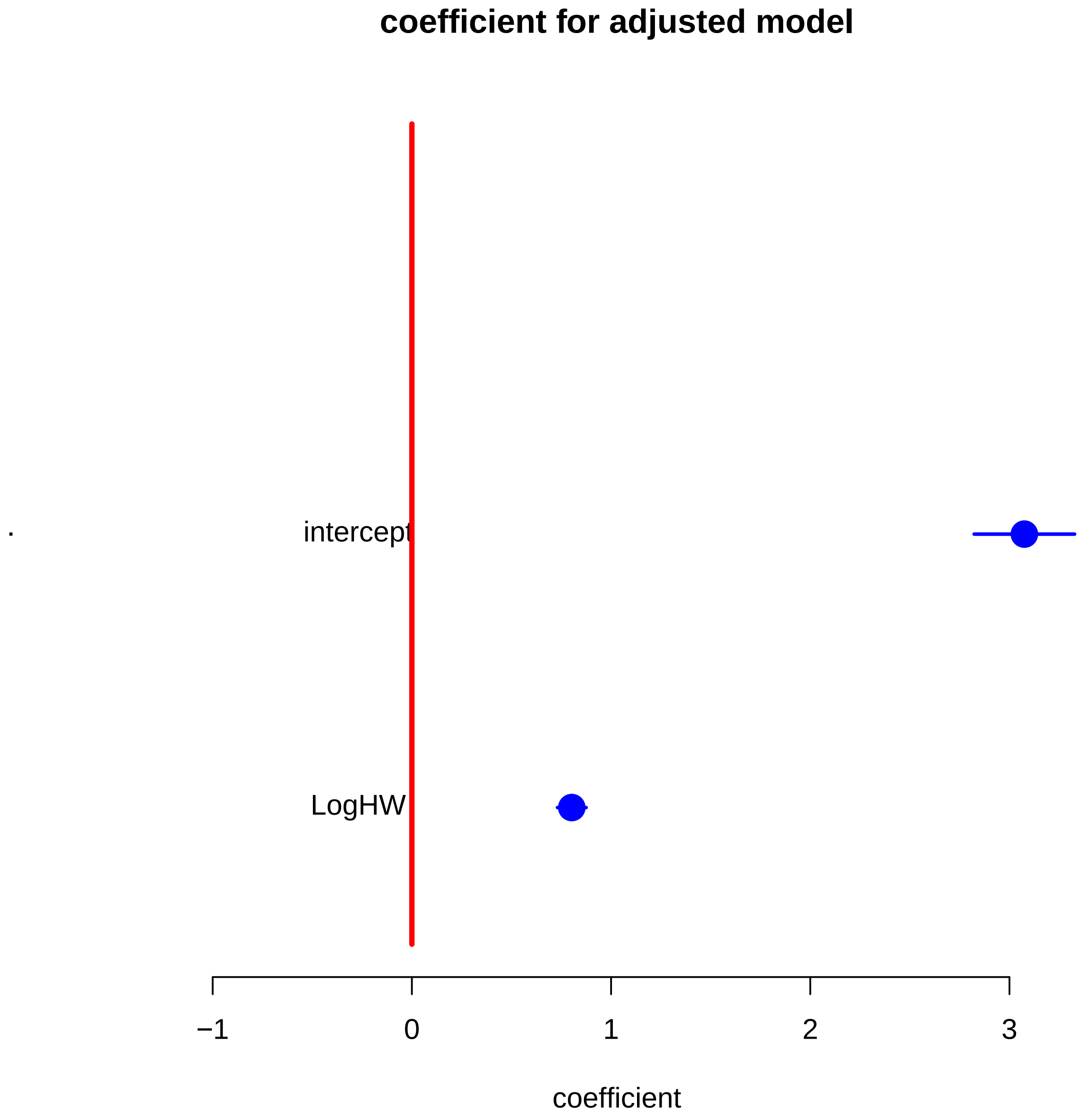
coefficient for adjusted model
intercept
LogHW
−1
0
1
2
3
coefficient

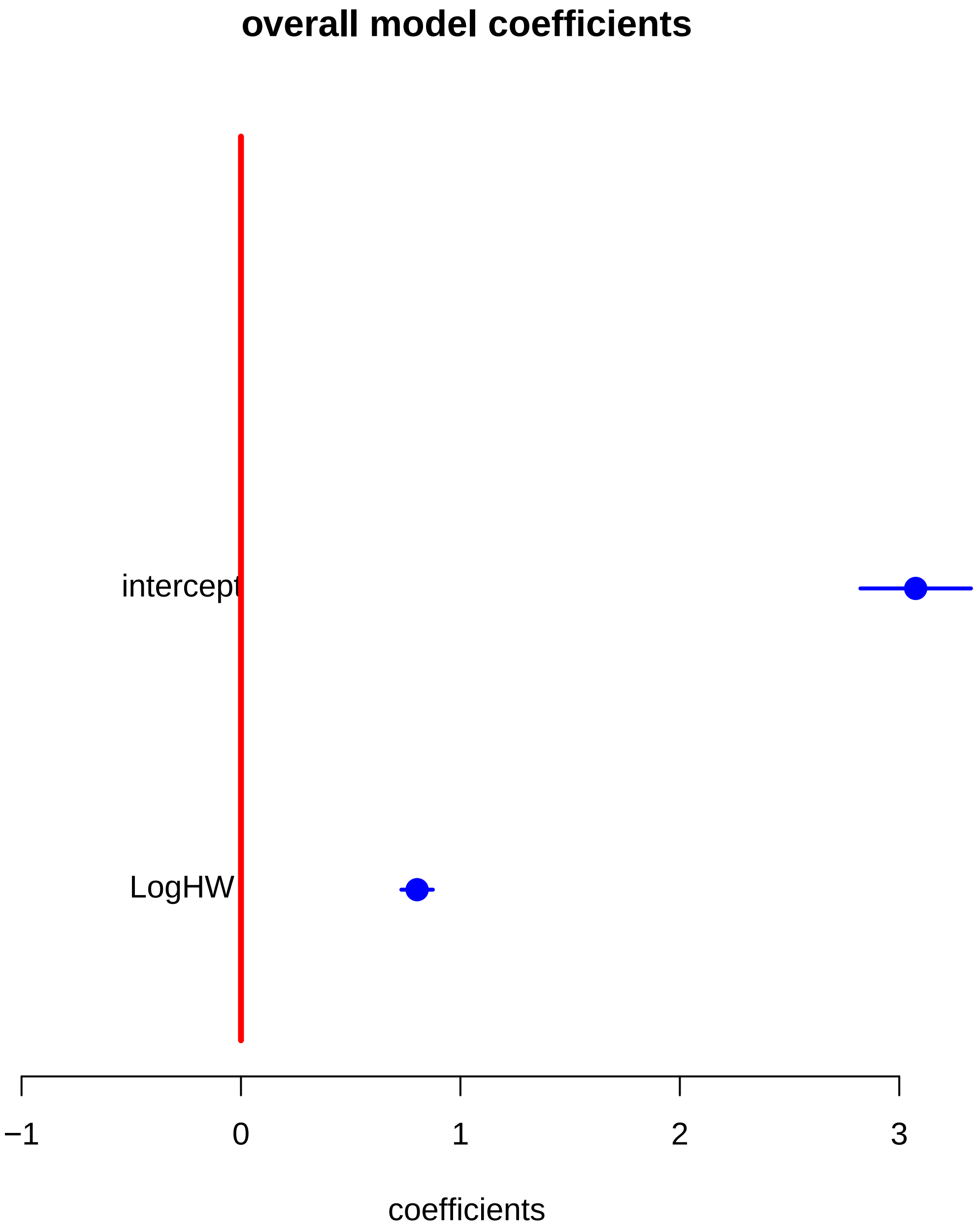
overall model coefficients
intercept
LogHW
−1
0
1
2
3
coefficients
