# Supplementary figures and images for: Expanded phylogeny elucidates Deinosuchus relationships, crocodylian osmoregulation and body-size evolution
Source: Commun Biol. 2025 Apr 23;8:611. doi: 10.1038/s42003-025-07653-4 (PMC12018936; doi:10.1038/s42003-025-07653-4)

# model checking for var

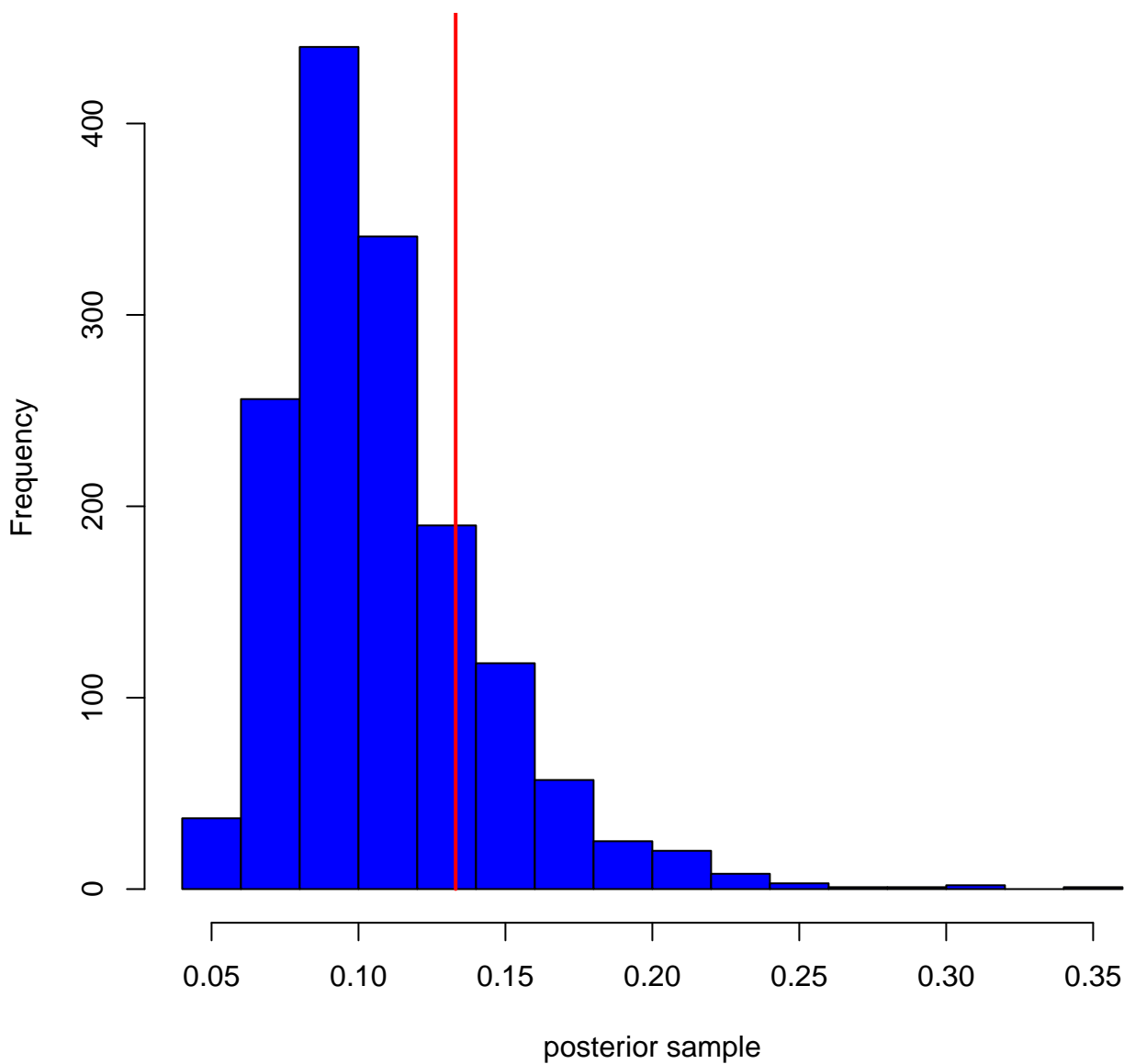

# model checking for mean

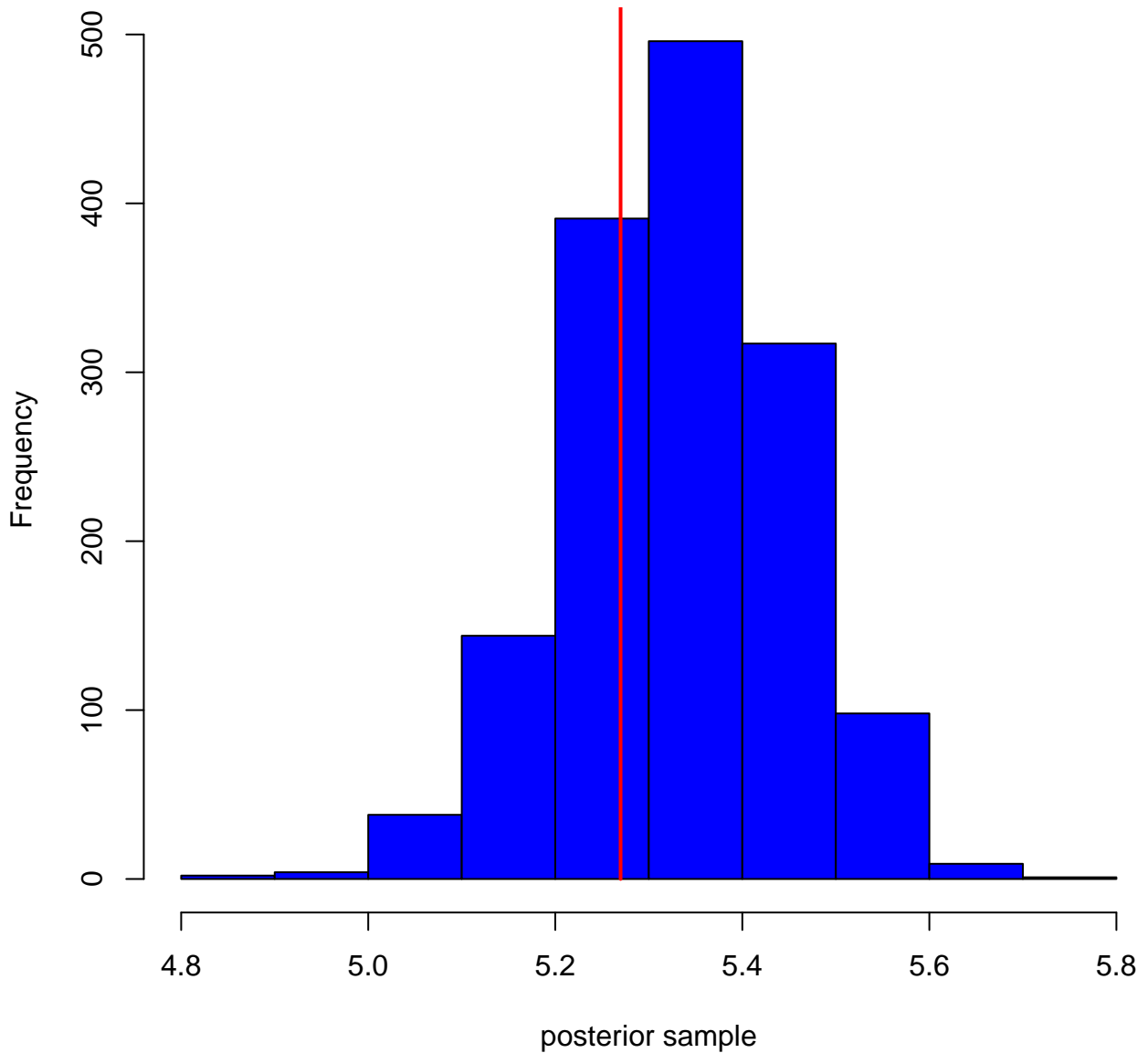

# model checking for min

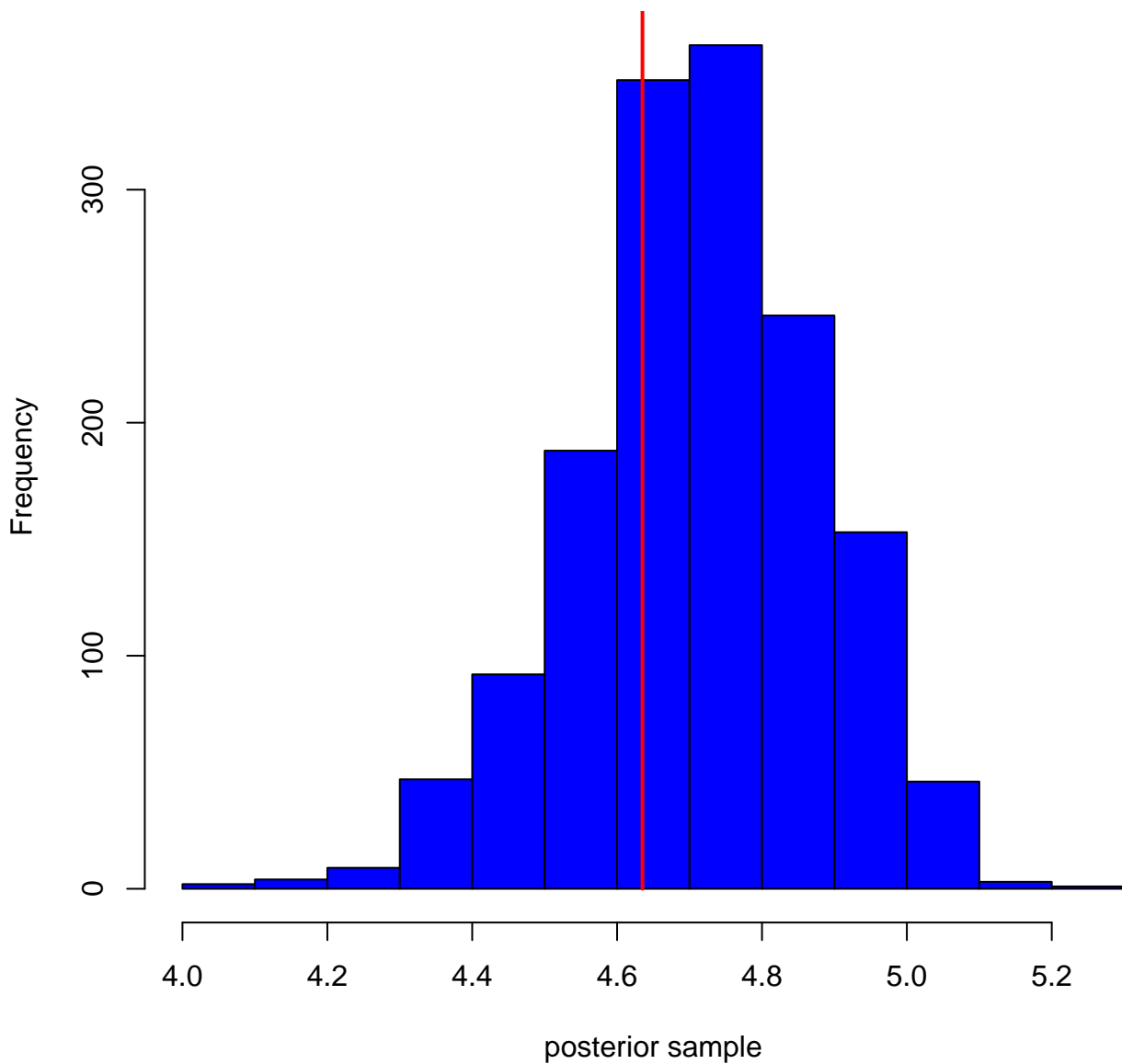

# model checking for max

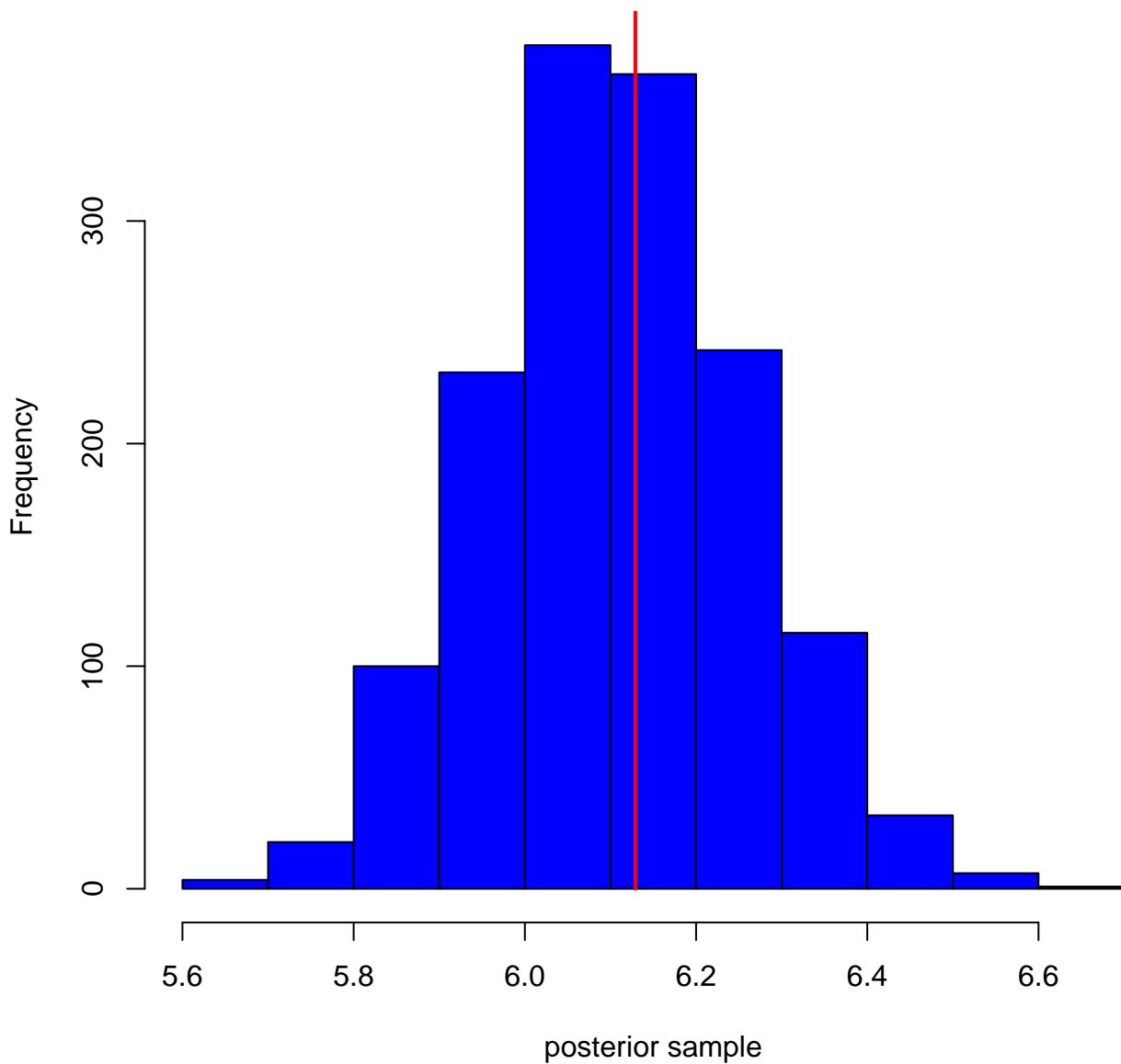

Supplement: Supplementary file 4 — Supplementary Data 2 [file 42003_2025_7653_MOESM4_ESM.zip › Supplementary Data 2/2. R Code - body size/modelChecking.pdf]
