# Supplementary material for: Expanded phylogeny elucidates Deinosuchus relationships, crocodylian osmoregulation and body-size evolution
Source: Commun Biol. 2025 Apr 23;8:611. doi: 10.1038/s42003-025-07653-4 (PMC12018936; doi:10.1038/s42003-025-07653-4)
Supplement: Supplementary file 5 — Reporting Summary [file 42003_2025_7653_MOESM5_ESM.pdf]

nature portfolio

Corresponding author(s): Jules D. WALTER
Marton RABI

Last updated by author(s): Nov 7, 2024

Reporting Summary

Nature Portfolio wishes to improve the reproducibility of the work that we publish. This form provides structure for consistency and transparency in reporting. For further information on Nature Portfolio policies, see our Editorial Policies and the Editorial Policy Checklist.

Statistics

For all statistical analyses, confirm that the following items are present in the figure legend, table legend, main text, or Methods section.

n/a	Confirmed	
☐	☒	The exact sample size (*n*) for each experimental group/condition, given as a discrete number and unit of measurement
☐	☒	A statement on whether measurements were taken from distinct samples or whether the same sample was measured repeatedly
☒	☐	The statistical test(s) used AND whether they are one- or two-sided<br>*Only common tests should be described solely by name; describe more complex techniques in the Methods section.*
☒	☐	A description of all covariates tested
☒	☐	A description of any assumptions or corrections, such as tests of normality and adjustment for multiple comparisons
☐	☒	A full description of the statistical parameters including central tendency (e.g. means) or other basic estimates (e.g. regression coefficient) AND variation (e.g. standard deviation) or associated estimates of uncertainty (e.g. confidence intervals)
☐	☒	For null hypothesis testing, the test statistic (e.g. *F*, *t*, *r*) with confidence intervals, effect sizes, degrees of freedom and *P* value noted<br>*Give P values as exact values whenever suitable.*
☐	☒	For Bayesian analysis, information on the choice of priors and Markov chain Monte Carlo settings
☒	☐	For hierarchical and complex designs, identification of the appropriate level for tests and full reporting of outcomes
☒	☐	Estimates of effect sizes (e.g. Cohen's *d*, Pearson's *r*), indicating how they were calculated

Our web collection on statistics for biologists contains articles on many of the points above.

Software and code

Policy information about availability of computer code

Data collection	Mesquite (version 3.70); ImageJ (version 1.54)
Data analysis	TNT (version 1.6); R (version 4.2.1); Mesquite (version 3.70)

For manuscripts utilizing custom algorithms or software that are central to the research but not yet described in published literature, software must be made available to editors and reviewers. We strongly encourage code deposition in a community repository (e.g. GitHub). See the Nature Portfolio guidelines for submitting code & software for further information.

Data

Policy information about availability of data

All manuscripts must include a data availability statement. This statement should provide the following information, where applicable:

- Accession codes, unique identifiers, or web links for publicly available datasets
- A description of any restrictions on data availability
- For clinical datasets or third party data, please ensure that the statement adheres to our policy

All data and codes are available in Supplementary information and Supplementary material. Accession codes in an publicly available repository will be provided prior to publication.

Research involving human participants, their data, or biological material

Policy information about studies with human participants or human data. See also policy information about sex, gender (identity/presentation), and sexual orientation and race, ethnicity and racism.

Reporting on sex and gender	No human participants were involved in the study.
Reporting on race, ethnicity, or other socially relevant groupings	No human participants were involved in the study.
Population characteristics	No human participants were involved in the study.
Recruitment	No human participants were involved in the study.
Ethics oversight	No human participants were involved in the study.

Note that full information on the approval of the study protocol must also be provided in the manuscript.

Field-specific reporting

Please select the one below that is the best fit for your research. If you are not sure, read the appropriate sections before making your selection.

☐ Life sciences ☐ Behavioural & social sciences ☒ Ecological, evolutionary & environmental sciences

For a reference copy of the document with all sections, see nature.com/documents/nr-reporting-summary-flat.pdf

Ecological, evolutionary & environmental sciences study design

All studies must disclose on these points even when the disclosure is negative.

Study description	Analysis of the origin of alligatoroids, through a novel topology obtained from the phylogenetic analysis of Eusuchia, using an expanded dataset of 128 crocodylian taxa and 219 morphological characters. The resulting evolution of body-length is also explored through a phylogenetically inferred body-size analysis.
Research sample	The research sample consists of 128 crocodylian taxa (including 16 extant), scored for 219 discrete morphological characters. The sample used for the phylogenetic body-size analysis consists in 165 specimens, including 91 fossils and 74 extant.
Sampling strategy	Sample is based on the existing character-taxon dataset of Massonne et al. (2019), further expanded by adding 23 new taxa, 20 characters, and updating over 150 character scores (using the studies of Gismondi et al. (2015), Bona et al. (2018), Cossette (2021), Stocker et al. (2021), and Walter et al. (2021)).
Data collection	JDW, TM and MR expanded the character-taxon dataset: updated the character scores and increased taxa sample. ALSP collected the cranial head width measurements for the phylogenetically inferred body-size estimates. All specimens referred in the study were sampled from literature search and personal observations of accessible material deposited in recognised museum collections.
Timing and spatial scale	Timing of data collection does not apply to the study, as it does not influence the results. The taxa sample has a global geographical distribution, from the Cretaceous to present time.
Data exclusions	No data was excluded from the analyses.
Reproducibility	All attempts to repeat the analyses were sucessful. All data and methods are made available in supplementary material for reproducibility.
Randomization	This section does not apply to the study. Randomization was not part of any of the methods used for the analyses performed in the study.
Blinding	This section does not apply to the study. Blinding was not part of any of the analyses performed in the study as no living participants were involved.

Did the study involve field work? ☐ Yes ☒ No

Reporting for specific materials, systems and methods

We require information from authors about some types of materials, experimental systems and methods used in many studies. Here, indicate whether each material, system or method listed is relevant to your study. If you are not sure if a list item applies to your research, read the appropriate section before selecting a response.

Materials & experimental systems

n/a	Involved in the study
☒	☐ Antibodies
☒	☐ Eukaryotic cell lines
☐	☒ Palaeontology and archaeology
☒	☐ Animals and other organisms
☒	☐ Clinical data
☒	☐ Dual use research of concern
☒	☐ Plants

Methods

n/a	Involved in the study
☒	☐ ChIP-seq
☒	☐ Flow cytometry
☒	☐ MRI-based neuroimaging

Palaeontology and Archaeology

Specimen provenance: N/A

Specimen deposition: N/A

Dating methods: N/A

☐ Tick this box to confirm that the raw and calibrated dates are available in the paper or in Supplementary Information.

Ethics oversight: N/A

Note that full information on the approval of the study protocol must also be provided in the manuscript.

Plants

Seed stocks: No plants material was involved in the study.

Novel plant genotypes: No plants material was involved in the study.

Authentication: No plants material was involved in the study.
